# Use of mHealth tools to register birth outcomes in low-income and middle-income countries: a scoping review

**DOI:** 10.1136/bmjopen-2022-063886

**Published:** 2022-10-12

**Authors:** Lottie Grace Cansdale, Gabriella Kelly, Ali Khashan, Address Malata, Fannie Kachale, David Lissauer, Simeon Yosefe, James Roberts, Simon Woodworth, Blandina Mmbaga, Christopher Redman, Jane Elizabeth Hirst

**Affiliations:** 1 University of Oxford Medical Sciences Division, Oxford, UK; 2 University College Cork School of Public Health, Cork, Ireland; 3 INFANT Research Centre, University College Cork, Cork, Ireland; 4 Malawi University of Science and Technology, Limbe, Malawi; 5 Malawi Ministry of Health, Lilongwe, Malawi; 6 Malawi-Liverpool-Wellcome Trust Clinical Research Programme, Blantyre, Malawi; 7 Institute of Life Course and Medical Sciences, University of Liverpool, Liverpool, UK; 8 Central Monitoring and Evaluation Division, Malawi Ministry of Health, Lilongwe, Malawi; 9 Magee-Women's Research Institute, Pittsburgh, Pennsylvania, USA; 10 University College Cork Business School, Cork, Ireland; 11 Kilimanjaro Clinical Research Institute, Kilimanjaro Christian Medical University College, Moshi, United Republic of Tanzania; 12 Nuffield Department of Women’s & Reproductive Health, University of Oxford, Oxford, UK

**Keywords:** obstetrics, information technology, health informatics

## Abstract

**Objective:**

Accurate reporting of birth outcomes in low-income and middle-income countries (LMICs) is essential. Mobile health (mHealth) tools have been proposed as a replacement for conventional paper-based registers. mHealth could provide timely data for individual facilities and health departments, as well as capture deliveries outside facilities. This scoping review evaluates which mHealth tools have been reported to birth outcomes in the delivering room in LMICs and documents their reported advantages and drawbacks.

**Design:**

A scoping review following Preferred Reporting Items for Systematic Reviews and Meta-Analyses and Joanna Briggs Institute guidelines for scoping reviews and the mHealth evidence reporting and assessment checklist for evaluating mHealth interventions.

**Data sources:**

PubMed, CINAHL and Global Health were searched for records until 3 February 2022 with no earliest date limit.

**Eligibility criteria:**

Studies were included where healthcare workers used mHealth tools in LMICs to record birth outcomes. Exclusion criteria included mHealth not being used at the point of delivery, non-peer reviewed literature and studies not written in English.

**Data extraction and synthesis:**

Two independent reviewers screened studies and extracted data. Common themes among studies were identified.

**Results:**

640 records were screened, 21 of which met the inclusion criteria, describing 15 different mHealth tools. We identified six themes: (1) digital tools for labour monitoring (8 studies); (2) digital data collection of specific birth outcomes (3 studies); (3) digital technologies used in community settings (6 studies); (4) attitudes of healthcare workers (10 studies); (5) paper versus electronic data collection (3 studies) and (6) infrastructure, interoperability and sustainability (8 studies).

**Conclusion:**

Several mHealth technologies are reported to have the capability to record birth outcomes at delivery, but none were identified that were designed solely for that purpose. Use of digital delivery registers appears feasible and acceptable to healthcare workers, but definitive evaluations are lacking. Further assessment of the sustainability of technologies and their ability to integrate with existing health information systems is needed.

**Strengths and limitations of this study**

- We have used a comprehensive search strategy to explore the literature regarding mobile health (mHealth) and digital birth registers in low-income and middle-income countries.
- We have reported data items using the mHealth evidence reporting and assessment checklist, which was developed by WHO to improve reporting of mHealth technologies.
- Only peer-reviewed articles have been included, meaning relevant data in the grey literature or technologies not reported in peer-reviewed articles may have been omitted.

## Introduction

Documentation of birth and perinatal events in low-income and middle-income countries (LMICs) and obtaining accurate statistics relating to mortality and morbidity can be challenging. Accurate data are needed to monitor health outcomes, to identify problems and to track the impact of interventions, as well as to understand the effects of health system shocks such as COVID-19. While electronic medical records can enable rapid monitoring of trends within units, regions and countries, such hospital-based electronic record systems are expensive, requiring large investments in computer hardware, networks and ongoing technical and security maintenance and support, all of which can be barriers for implementation in LMICs.

Traditionally, births in LMICs are recorded in paper-based delivery registers. These are usually large books, completed by birth attendants soon after the birth. While their content and quality can be variable, they are often the only source of data for collation of perinatal health statistics. Paper-based registers have a short memory and are difficult to store due to their large size. The information in delivery registers is difficult to access when it is needed for patient care at a later date, for comparing data between institutions, or for monitoring trends in care and outcomes over time. Furthermore, non-institutional births may not be recorded at all. LMICs have a high proportion of non-facility births, for example, only 50% of births in Ethiopia, 59% in Bangladesh and 70% in Guatemala take place in healthcare facilities.^1^ LMICs are also the countries with the highest maternal and neonatal mortality rates; maternal deaths per 100 000 are estimated at 375 in Uganda, 342 in Kenya and 177 in Indonesia.^2^ In other words, those most at risk and in need are those least likely to be documented.

The use of mobile phones is increasing rapidly. One report suggested ownership rates of 80% in ‘emerging economies’, of which 47% are smartphones.^3^ Given the increasing ubiquity of smartphones, their use could improve reporting of delivery and birth outcomes. This application is termed mobile health (mHealth), and is defined by WHO as ‘the use of mobile wireless technologies for health’.^4^ mHealth is already being used in the field of maternal health, with one systematic review identifying 19 different studies describing 15 mHealth interventions that addressed maternal health.^5^ This review found mHealth being used for a variety of purposes, including appointment reminders, health promotion, provider-to-provider communication and data collection. However, at present it is unclear to what extent mHealth is used to capture birth data at the point of delivery, whether this approach is acceptable to healthcare workers and whether it improves recording of perinatal outcomes. Hence, the purpose of this scoping review is to understand what mHealth technologies have been evaluated to record birth outcomes at the time and place of delivery in LMICs.

## Methods

This review was developed in line with the Preferred Reporting Items for Systematic Reviews and Meta-Analyses and Joanna Briggs Institute guidelines for scoping reviews,^6 7^ using the PCC mnemonic—population, concept, context. A scoping review gives an overview of the evidence, maps the literature and identifies and analyses current gaps in knowledge. A review protocol was not published for this study.

### Population, concept, context

#### Population

The population of this review comprises healthcare workers recording delivery outcomes, namely midwives, community healthcare workers (CHWs), doctors and other cadres of skilled birth attendants in LMICs. LMICs were defined according to the World Bank categorisation of low, lower-middle and upper-middle in 2021.^8^

#### Concept

Our focus is the use of mHealth to capture data at birth, and specifically those technologies present at the place of delivery. Of these many are expected to have wider applications, such as recording of the outcomes of antenatal and postnatal appointments, or progress in labour via an e-partogram. For this scoping review, we have included any technology if outcomes at birth were recorded by the birth attendant or CHW, at the time of, or shortly after, delivery.

#### Context

The context of the review are the places where births occur in LMICs. This includes home and community deliveries, as well as institutional births in health centres, nursing homes and government or private hospitals.

### Search strategy

To identify relevant sources, three databases were searched: PubMed, CINAHL and Global Health. The search strategy was created with an experienced librarian and further refined by the authors, including key search terms such as ‘childbirth’, ‘mobile applications’ and ‘midwives’. The final search strategies for each database can be found in the online supplemental appendix 1. The databases were searched for relevant articles up to 3 February 2022, with a lower date limit not specified as the term ‘mHealth’ was not widely used prior to 2000. The final searches were uploaded into EndNote and duplicates were removed by their duplicate detection software.

10.1136/bmjopen-2022-063886.supp1 Supplementary data

### Screening and selection of relevant studies

Once the citations had been extracted to EndNote, they were uploaded on to Colandr (colandrapp.com), a software that manages citation and full-text screening for reviews. Texts were first screened by abstract, and those that met the inclusion criteria then went on to the full-text screen. Two reviewers (LGC and GK) did both citation and full-text screening of articles, and any disputes between reviewers were resolved by a third author (JEH).

References of studies included for full-text screening were hand-searched to identify for further relevant studies. These hand-picked citations were uploaded to EndNote and then included in citation screening. Once imported into EndNote duplicates were removed.

### Inclusion and exclusion criteria

Eligible studies were conducted in LMICs (defined by the World Bank list of LMICs).^8^ Within each study, the mHealth tool needed to be capable of collecting data regarding birth outcomes at the place of delivery. We considered studies where the data were collected by nurses/midwives/birth attendants. We limited the search to English language publications only.

We excluded studies where no birth outcomes were reported in the publication, where data were collected using hospital electronic health record (EHR) systems on desktop computers, where birth registration data were collected after the delivery or for the purpose of vital registration only, and where data were collected by people other than nurses/midwives/birth attendants. As this was intended as a scoping review of published literature, we excluded non-peer-reviewed reports of technologies, such as reports, web pages and media releases.

### Data extraction and synthesis

An Excel spreadsheet was used for data extraction, with variables to extract agreed through discussion between the authors. The two reviewers (LGC and GK) independently charted data and discussed results.

We abstracted data on article characteristics, including country of origin, funder, form of mHealth, technology user, reported birth outcomes and technology feasibility and acceptability.

We conducted a narrative synthesis based on our findings. We first mapped studies by geographic location, technology type, primary intended use of technology, accessibility outside the programme described (ie, whether the technology was Open Access or not) and intended user group (hospital/clinic staff or in the community). We then identified the main thematic areas that have been evaluated to date, with the purpose of summarising the current state of the academic literature in this field and identifying gaps for further research. We made particular use of mHealth evidence reporting and assessment (mERA), a checklist which was developed by WHO to standardise mHealth evidence reporting.^9^ Given this was a scoping review to map the quantity and domains of evidence on digital birth registers, the quality of studies was not assessed.

### Patient and public involvement

Patients and/or the public were not involved in the design, or conduct, or reporting, or dissemination plans of this research.

## Results

### Identification of eligible studies for review

**Figure 1:**
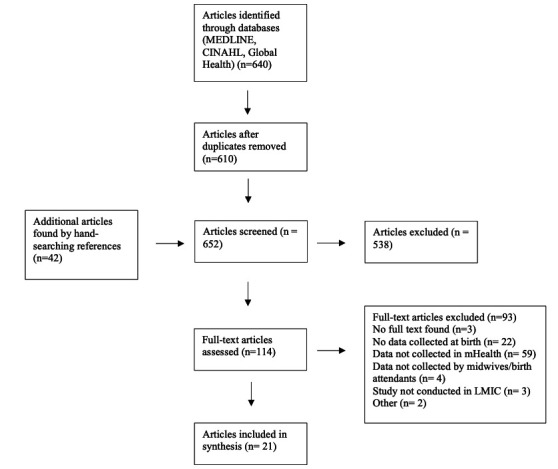
Screening and selection of articles. MEDLINE, CINAHL and Global Health were searched for relevant references. These were first screened by citation then full text. Articles selected for inclusion were then hand-searched for relevant references. LMIC, low-income and middle-income country.

The search strategy is outlined in figure 1. The initial search was carried out on 31 July 2021 and updated on 3 February 2022, yielding 567 and 640 records, respectively. Of the records identified, 578 were from PubMed, 34 from CINAHL, 28 from Global Health.

### Study characteristics and themes

The characteristics of each study are described in table 1, together with the six specific themes we identified. The themes are elaborated in more detail in the results below. Table 2 describes the technical aspects and content of interventions.

**Table 1:** Study characteristics and themes

Technology, Study	Country	Study design,* N participants	Labour†	Outcomes‡	Comm§	Attitudes¶	Paper versus elect**	Sustain††
1. ePartogram								
Litwin *et al*^10^	Tanzania	Feasibility and acceptability 23 skilled birth attendants	●	●	●	●	●	●
Sanghvi *et al*^11^	Kenya	Mixed method, quasi-experimental 110 skilled birth attendants	●	●	●	●	●	●
2. E-partograph								
Rahman *et al*^12^	Bangladesh	Prospective crossover study Nurse-midwives (NNS)	●	●	●	●	●	●
Tadesse *et al*^13^	Ethiopia	Cross-sectional study with questionnaire 462 obstetric care providers	●	●	●	●	●	●
3. mLabour								
Schweers *et al*^20^	India	Design and preliminary evaluation 12 nurses	●	●	●	●	●	●
4. prasavGraph								
Singh *et al*^15^	India	Development and pilot Doctors and student nurses (NNS)	●	●	●	●	●	●
5. ASMAN								
Usmanova *et al*^21^	India	Qualitative study 44 stakeholders	●	●	●	●	●	●
Usmanova *et al*^18^	India	Secondary analysis of programme data (NNS)	●	●	●	●	●	●
6. PPH reporting								
Andreatta *et al*^22^	Ghana	Evaluation of technology 10 birth attendants	●	●	●	●	●	●
7. Mobile Delivery Timer								
Somannavar *et al*^23^	India	Development and evaluation 46 birth attendants	●	●	●	●	●	●
8. Birth defects surveillance system								
Mumpe-Mwanja *et al*^19^	Uganda	Surveillance study Midwives (NNS)	●	●	●	●	●	●
9. RapidSMS-MCH								
Ngabo *et al*^24^	Rwanda	Design and implementation 432 community healthcare workers	●	●	●	●	●	●
Musabyimana *et al*^25^	Rwanda	Qualitative study 93 participants including community healthcare workers	●	●	●	●	●	●
10. Data collection								
Little *et al*^26^	Ethiopia	Development and evaluation 12 midwives, 20 health extension workers	●	●	●	●	●	●
Medhanyie *et al*^27^	Ethiopia	Semi-structured questionnaire 23 midwives and health extension workers	●	●	●	●	●	●
Medhanyie *et al*^30^	Ethiopia	Comparative cross-sectional study 25 midwives and health extension workers	●	●	●	●	●	●
11. Perinatal monitoring app								
Stroux *et al*^16^	Guatemala	Prototype testing 9 healthcare workers	●	●	●	●	●	●
Martinez *et al*^29^	Guatemala	Agile development 25 midwives	●	●	●	●	●	●
Martinez *et al*^14^	Guatemala	Randomised control trial 44 traditional birth attendants	●	●	●	●	●	●
12. Hayat								
Zaidi *et al* 28	Pakistan and Afghanistan	Qualitative exploratory study 54 female healthcare workers, 42 community healthcare workers	●	●	●	●	●	●
13. Maternal referral mobile system								
Indriani *et al*^17^	Indonesia	Development of technology Midwives (NNS)	●	●	●	●	●	●

Key: ●=not described, ●=described, ●=partially described.

*Study design taken from description in paper.

†Digital tools for labour monitoring.

‡Digital collection for specific birth outcomes.

§Digital tools for use in the community.

¶Attitudes of healthcare workers towards digital collection of birth outcomes.

**Comparison of paper and electronic data tools.

††Infrastructure, interoperability and sustainability.

NNS, number not stated.

**Table 2:** Technical aspect and intervention content

Name	Platform	Interoperability/HIS context	Intervention content
1. ePartogram	Android tablet app	Data accessible at referral facilities	Mobile partograph with automatic graphing of data, clinical decision support and auditory alerts
2. E-partograph	Android smartphone or tablet app	Partograph data monitored remotely	Mobile partograph with automatic graphing of labour progress and alerts for abnormalities
3. mLabour	Mobile app	NS	Mobile partograph and labour management tool to register patients, record measurements and record delivery
4. prasavGraph	Android smartphone or tablet app	Birth registration with municipal authorities	Mobile partograph with digital record keeping and notifications for abnormalities
5. ASMAN	Tablet based	Integrated into government databases	Platform with digital case sheets, dashboards, E-learning content and remote support centre
6. PPH monitoring	SMS based	NS	Numeric SMS protocol to report maternal demographics and birth outcomes
7. Mobile Delivery Timer	Android smartphone app	NS	Application with voice recording to verbally mark crowning, birth, crying or bag mask ventilation of baby
8. Birth defect surveillance	Android tablet app	NS	Application to document birth defects and record birth outcomes
9. RapidSMS	Open-source app	Automatic notification to ambulance service	SMS protocols to record pregnancy, maternal and child outcomes, allows communication between CHWs and health facilities
10. Data collection tool	Smartphone app	Local health authority	Application with maternal healthcare forms and analytics dashboard
11. Perinatal monitoring system	Android smartphone app	Open MRS	Tools to record maternal vital signs, clinical decision support tool, supports communication between birth attendants and medical team
12. Hayat	Smartphone app	Local health authority	Mobile application to digitalise data entry, and dashboard to visualise health reports
13. Maternal referral mobile system	Smartphone app	Data available at referral centre	Application to communicate maternal referrals between primary healthcare and referral hospital

CHW, community healthcare worker; HIS, health information system; NS, not stated.

We identified 15 different mHealth technologies, across 12 different countries: 7 in Africa, 7 in Asia and 1 in South America. A variety of health workers were end-users of the technologies, including midwives, nurses, traditional and skilled birth attendants (TBAs and SBAs) and CHWs.

Six technologies were described in more than one paper. The ePartogram and E-partograph were studied in two different settings: Tanzania and Kenya for the ePartogram,^10 11^ and Bangladesh and Ethiopia for the E-partograph.^12 13^ The further studies on the other four technologies (RapidSMS, the data collection tool, perinatal monitoring app and ASMAN) were conducted in the same setting as the first published paper.

A variety of methods were used to assess the technologies, with the majority being early phase development and pilot work. Only one study reported results of a randomised controlled trial,^14^ while three studies were preclinical.^15–17^ Sample sizes also varied between studies: the largest was 462^13^ and smallest 9.^16^ Five studies did not document the number of healthcare workers using the technology.^12 15 17–19^

### Roles for the digital tools

The six themes identified were split into roles for technologies and focus of studies. The three main roles for digital tools at birth were: (1) labour monitoring; (2) recording of a specific birth outcome; (3) tools for use in the community, and studies were mainly concerned with: (1) attitudes of healthcare workers to digital technologies; (2) comparison of paper and digital data collection and (3) sustainability, interoperability and infrastructure for mHealth interventions.

### Digital tools for labour monitoring

Eight of the studies described tools that could monitor labour, all based on WHO partograph^10–13 15 18 20 21^ and are shown in table 3. While the primary purpose of the partograph is to monitor labour, these technologies had additional capabilities to record birth data, often to assess the impact of labour monitoring on birth outcomes.

**Table 3:** Digital tools for labour monitoring

Technology	Studies
ePartogram	10 11
E-partograph	12 13
mLabour	20
prasavGraph	15
ASMAN	18 21

Several electronic partographs have been described. The ePartogram was developed by John Hopkins University and their NGO arm, Jhpiego. Study of its use in Tanzania showed SBAs were comfortable registering clients and entering data into the application and had a preference for the ePartogram over paper versions.^10^ This was replicated in a study in Kenya, where it was also found to improve birth outcomes compared with paper partograph use.^11^ Improvements in birth outcomes was also seen in studies investigating the E-partograph in Bangladesh.^12^ However, a separate study of the E-partograph in Ethiopia found only 46% of care providers would be willing to use a mobile-based E-partograph, despite 65.8% of caregivers reporting they wished to use a partograph routinely.^13^

Three other technologies were identified that had a digital partograph as part of their workflow. prasavGraph is an app incorporating a partograph with other data collection, including the baby’s sex, weight and condition at birth, reported in preclinical phase in New Delhi.^15^ mLabour also incorporated a partograph into their labour management tool; nurses using it reported being more punctual with data entry and found the tablet form lighter and easier to record data as compared with multiple paper registers.^20^ This feedback was echoed with the ASMAN application, which consists of a digital case sheet for patients, partograph, dashboard for health facilities and E-learning content, and was used in both district hospitals and community health centres.^21^ Analysis of application data showed high rates of data collection for delivery in the app, at 93.7%.^18^

### Digital collection for specific birth outcomes

Three technologies were designed to report a specific outcome, and recorded delivery details as part of the workflow. A system for community midwives to report postpartum haemorrhage (PPH) was designed in Rwanda, using mobile phone handsets and SMS protocols.^22^ In addition to PPH, data were collected on maternal and neonatal deaths. Ten TBAs used the technology and successfully reported 425 births and 13 incidents of PPH.

The other two studies collected data collected on tablets. The Mobile Delivery Timer was designed to measure the time between delivery and either the baby crying or initiation of bag mask ventilation, as part of a larger ‘Helping Babies Breathe’ study.^23^ This recorded the time of delivery as well as the woman’s ID and status of the newborn. Ninety per cent of birth attendants found recording data in the mobile phone either ‘moderately easy to use’ or ‘very easy to use’. Android tablets were used in a tool to detect birth defect prevalence in Uganda.^19^ All births were examined by a midwife as soon as feasible and data collected on forms designed using Open Data Kit, including both pictures of any major defects as well as quantitative data documenting maternal age, birth outcome, newborn sex and mode of delivery. The system was able to detect a birth defect prevalence of 66 per 10 000 births, but the authors noted there were no population-based data for comparison.

### Digital tools for use in the community

Six of the tools were designed specifically to be used in the community, either by trained midwives or lay or volunteer healthcare workers.

Two technologies used mobile phones and SMS protocols to capture community data. The PPH reporting system is one example,^22^ as is the RapidSMS-MCH system in Rwanda.^24^ In Rwanda, volunteer CHWs were given mobile phones with eight SMS forms for reporting data, including one for delivery. The SMS was sent to a central server and could alert an ambulance in case of emergency. The technology was scaled up nationwide in 2013, and a further study found it was well accepted by both healthcare workers and the local community.^25^

A further three technologies used smartphones to record data collected by CHWs. An app was designed for data collection by midwives and health extension workers (HEWs) in Ethiopia, containing eight data collection forms for different perinatal events.^26^ It also contained a scorecard and analytics dashboard to allow data monitoring by local health bureaus. The app was adapted for use in the community by using the local calendar and local language. Analysis of the records submitted found that most delivery protocols were submitted by midwives rather than HEWs, reflecting local guidance that HEWs should not assist with deliveries.^27^ A similar format was seen with the Hayat app in Afghanistan and Pakistan; it was designed to digitalise data entry, and also contained a dashboard to visualise reports generated by patient encounters.^28^ CHWs were able to successfully use the app but highlighted issues such as the app not using the local language. The perinatal monitoring tool used in Guatemala was adapted for community use by having audio and visual instructions and data collection through audio recordings, to be inclusive of illiterate and technology-naïve end-users.^16 29^ Similarly, to the other technologies, it collects data based on antenatal, postnatal and intrapartum encounters, combined with recording of vital signs. Once assessment is complete, the data are uploaded to the patient’s OpenMRS electronic medical record. An initial feasibility study showed use was feasible among traditional birth attendants, and later randomised control testing showed that use of the app was associated with improved detection of pregnancy complications and a higher rate of referrals to medical facilities.^14^

Several of the tools were designed to aid communication between health professionals. A mobile referral system was designed in Indonesia, allowing midwives to relay information about patient condition and neonatal outcomes to the referral hospital.^17^ This was a feature of other platforms: the perinatal monitoring system alerted the local health facility if a complication was detected,^14^ and RapidSMS could automatically forward ambulance requests in an emergency.^24^ ASMAN also facilitated communication, through access to a remote support centre staffed by health personnel.^21^

### Attitudes of healthcare workers towards digital collection of birth outcomes

Ten studies explored the attitudes of healthcare workers towards digital tools. Seven used focus groups or in-depth interviews^10 11 20 21 25 26 28^ and three used questionnaires.^13 23 27^ All but one of the studies reported that the technology was well received and preferred to conventional methods of data collection.

Four studies were specifically designed to explore the preferences of healthcare workers.^13 21 25 27^ Generally they showed positive attitudes towards digital data collection; one study in Ethiopia found 87% of healthcare workers perceived it to be useful,^27^ and ownership of the mobile phones was a motivating factor for using the technology for 91.3%. The RapidSMS project also gave mobiles to healthcare workers, and while the technology was perceived to improve birth outcomes, the increased reporting requirement reduced the motivation of the volunteer CHWs, leaving some wanting financial compensation for the increased workload.^25^ Only one study used a validated tool to study health worker preferences; the Technology Acceptance Model, which explores perceived ease of use and usefulness of a technology, was used to evaluate the ASMAN application.^21^ The application was reported to be easy to use and less error prone compared with paper, and improved clinical documentation by prompting users to ask for additional information as necessary.

Only one study showed a negative attitude towards a digital technology.^13^ Analysis of attitudes towards the E-partograph showed that while 99.6% of workers owned a mobile phone, only 46% were willing to use a mobile-based E-partograph; however, this did not specify whether they were unwilling to use the app on their personal phone or unwilling to use the E-partograph in general. It was noted that 58.9% of those surveyed had a favourable attitude towards the partograph, and this was associated with willingness to use the E-partograph, so the authors concluded that the results may be due to negative attitudes towards the partograph in general rather than an electronic tool specifically.

In six studies, the time taken to use digital technologies was mentioned by healthcare workers. In three, digital data collection was perceived to be a timesaver.^10 21 28^ However, those using the ePartogram were concerned about achieving timely data entry in high volume sites or with multiple clients,^11^ and 78.3% of those using the Ethiopian data collection tool cited time taken as a reason for not using forms all the time.^27^ With ASMAN, while facilities using the application alone to collect data found it reduced reporting time, some facilities required data collection electronically and on paper for verification, audit or in case of paper issues, which increased workload temporarily.^21^

### Comparisons of paper and electronic tools

mHealth tools were formally compared with the current standard of reporting, paper records, in three of the studies.^11 12 30^ This was either comparing preferences for paper or electronic data collection or comparing completeness of records.

The current WHO partograph is a paper version, so studies using an electronic version often used this as a comparison. One crossover study comparing the E-partograph with the paper version found higher user rates during the electronic phase in both sites.^12^ Another study found that all measurements except fetal heart rate and contractions were recorded more frequently in the ePartogram compared with paper.^11^ However, SBAs found that it was more difficult to correct errors in data entry using the ePartogram, which was not an issue with the paper charts.

Similar findings were seen with the data collection tool in Ethiopia.^30^ Comparing the completeness and accuracy of patient data collection on electronic forms with paper, they found overall completeness was 8% higher in electronic records compared with paper. While there was no difference for parameters such as patient name or age, for newborn weight recording was 20.5% higher in electronic forms. The authors speculated this could be because the electronic forms required a response to be accepted. However, they also found some workers lacked the apparatus to measure some parameters, such as scales for recording newborn weight, and had thus been discouraged from using electronic forms to record data due to mandatory reporting requirements, meaning they were more likely to use paper and reduce the completeness of paper records. The study reported that missing data on paper forms was also due to lack of physical space on paper sheets.

### Infrastructure, interoperability and sustainability

Few studies included information about the infrastructure necessary for mHealth technology. Some described the hardware given to study participants, for example, in Ethiopia workers were given secondhand HTC Hero smartphones, solar lamp and phone chargers.^26^ They reported that of the 23 health posts in the area, only 48.9% had GPRS at the time, so only chose health posts with connectivity for the study. Network connectivity varied across the studies, with some reporting 3G being available in all facilities,^10^ whereas those using the Hayat app cited a lack of connectivity as a barrier to uploading data.^28^ The Hayat app had also GPS capabilities, so that healthcare workers could be tracked during outreach visits; however, workers had to travel to the central offices once per week to upload data to the central server, which was also the case with the perinatal monitoring system in Guatemala.^16^

Four studies reported capability to communicate with pre-existing health information systems (HIS) or upload data to EHR and electronic medical records (EMR).^16 21 26 28^ The perinatal monitoring app in Guatemala has been used in the community and is able to upload data to the OpenMRS EMR once assessment is complete.^16^ However, in the randomised controlled trial of the application, this feature was not mentioned.^14^ The data collection project in Ethiopia discussed uploading their data to EMRs but stated that improvements in patient identification would be needed for this to be successful.^26^ However, the Ethiopian data collection app, the Hayat app^28^ and the ASMAN package^21^ all created a dashboard from data collection reports submitted, which allowed local health bureaus to view the data. Beside connecting to a HIS, prasavGraph reported that their data could be used to record births with municipal authorities, but this has not progressed beyond prototype testing.^15^

Table 4 contains a summary of user feedback, contextual adaptability and limitations for delivery at scale. Not all the studies considered sustainability of the intervention, or the feasibility of scaling technology up. Scaling-up was discussed with regard to ASMAN, detailing a transition package to move past the pilot phase involving transferring the application to the states’ servers and integrating ASMAN data fields into government bases.^21^ However at the time of writing, only RapidSMS-MCH had been scaled up, to involve all CHWs across Rwanda.^25^

**Table 4:** Limitations and contextual adaptability

Name	User feedback	Limitations for delivery at scale	Contextual adaptability
1. ePartogram	Improved decision making Data entry simple and user-friendly	Concerns about time for data entry in high-volume labour wards	Feedback session with SBAs done as part of development process
2. E-partograph	44% were willing to use 58.9% had favourable attitudes towards the partograph	Obstetric care providers less willing to use	NS
3. mLabour	Users preferred tablet format to paper Requests for additional functionality, for example, tracking medications	NS	Some features of app customised for context of specific hospital, not wider use
4. prasavGraph	NS	NS	Designed for use in settings with poor network connection
5. ASMAN	Technology easy to use Perceived improvement in job performance	Technology and internet issues High caseloads prevented all fields being completed	E-learning written in English and Hindi with audio, visual and readable formats
6. PPH monitoring	NS	Poor network and power outages Lack of remuneration	Training given in English and native language
7. Mobile Delivery Timer	90% found application easy to use 70% felt app was beneficial	NS	Android operating system chosen as functions on low-cost hardware
8. Birth defect surveillance	NS	System does not capture babies born outside of urban facilities	NS
9. RapidSMS	Workers reported being more pro-active in finding pregnant women Requests for financial compensation	High initial cost Lack of equipment prevented all data being collected	Mobile phones provided to CHWs to reduce their costs
10. Data collection tool	52.2% said forms were comprehensive 87% found electronic forms helpful Preferred electronic forms to paper	High cost of covering phone airtime	Provided with solar lamp to charge smartphone
11. Perinatal monitoring system	System was easy to operate with potential benefit	Unfamiliarity with technology Low-quality ultrasound recordings	Audio and visual instructions used to adapt user interface for illiterate users
12. Hayat	App use feasible and improved efficiency Frustrated at inability to edit entries	CHWs needed to travel long distances each week to sync app data with central server	NS
13. Maternal referral mobile system	NS	Bugs in application	Focus group interviews conducted prior to development

CHW, community healthcare worker; NS, not stated; SBA, skilled birth attendant.

Most of the technologies used Android hardware, which is widely available in LMICs and is relatively low cost; one study cited its use as a way to increase sustainability.^23^ The sustainability of the perinatal monitoring system in Guatemala was assessed in relation to its cost which was <US$50 for the equipment, while its software was open source.^29^ The US engineers involved in the development of the app also spent time training engineers in Guatemala to be able to deal with software and hardware issues relating to the technology.^29^ Issues in scaling up mHealth use were highlighted in the Ethiopian study where workers were given cell phones; they said that the cost of airtime was high owing to workers using phones for personal and study purposes. They suggested that the intervention would be more sustainable if a mechanism could be found either to provide free airtime for uploading protocols or to restrict airtime to study protocols.^27^

## Discussion

We identified 21 studies describing 15 mHealth technologies that could collect data in the delivery room. Studies showed that data collection at delivery is both feasible and acceptable to healthcare providers. Of the six themes we identified, three were the primary purposes technologies were designed for: labour monitoring, collection of a specific birth outcome and data collection in the community. While no technology was specifically designed to record birth outcomes in the delivering room, our findings suggest that use of such a tool would be feasible and acceptable.

All but one of the studies found that digital data collection was preferred to paper methods. Digital technologies were reported to be easy to use,^21 23^ user-friendly^11^ and perceived to improve worker efficiency^28^ and birth outcomes.^25^ Healthcare workers also preferred the digital format compared with paper methods, noting them to be less bulky to carry and easier to access.^20 28^ It was also reported that digital collection could improve the number of data items reported, by incorporating mandatory data entry fields.^21 25^

We found that the time taken to input data can be a barrier. In some studies, this study design requiring paper and electronic entry rather than issues with the tool itself.^21 27^ However, we also found evidence that healthcare workers were concerned about time to input data,^26^ particularly in facilities caring for large numbers of patients.^11^ Digital tools can mandate reporting of more items, and while this is useful for data collection and research, it can be time-consuming for healthcare workers and reduce motivation to record data.^25^ An mHealth application that did not meet criteria for inclusion, mClinic, sought end-user feedback on items for data reporting, finding that users only wanted items that would be included in reports and did not want free-text for reporting of subjective data.^31^ Clearly, end-user involvement in designing data collection protocols is important to ensure that each data item is perceived to be useful for health worker’s practice.

We found that there are several gaps in reporting of interventions in the published literature. One of the items in the mERA checklist, developed by WHO, is ‘interoperability/HIS context’, which involves ‘describing how mHealth intervention can integrate into existing HIS’.^9^ Our review found few studies describing how the mHealth tool would integrate with the existing HIS; only the perinatal monitoring app appeared to upload data directly to patient’s medical records app.^16^ Another item in the mERA checklist is ‘cost assessment’, and again only the perinatal monitoring app mentioned the cost of the hardware required to run the technology.^16^ Consideration of both cost and interoperability are crucial for the sustainability,^32^ so the lack of reporting in the literature highlights the need for both standardised reporting of interventions and for authors to consider these factors when designing and testing new technology.

Few tools were identified with the capability to record births in both facilities and in the community. While several of the community-based technologies could communicate data to facilities, only the Ethiopian data collection tool was used to collect data by both healthcare workers in the community and midwives in health centres.^26^ Since the proportion of non-facility births in many LMICs is high,^1^ a digital birth register would need to be designed for use in both the community and facilities to ensure maximum coverage of births.

To our knowledge, no other review has considered the use of mHealth specifically in the delivering room, or its potential to replace paper-based delivery registers. However, our review is in agreement with other reviews that there is a lack of high-quality evidence and robust study design to determine the efficacy and effectiveness of mHealth interventions in maternal healthcare.^5 33 34^ Of the studies we identified, only 1 was a randomised controlled trial, and of the 15 technologies only 6 were evaluated by more than 1 study. With regard to the purpose of evaluation, only two studies formally assessed the accuracy of digital data collection compared with paper methods,^11 30^ and only six studies explored the time needed for digital input relative to paper recording.^10 11 21 27 28^ The lack of quality evidence creates a barrier to scaling up technologies and emphasises the need for pilot studies to be followed-up with studies that investigate scalability.^32^

Our own review has its strengths and limitations. While we used a comprehensive search strategy, we only included peer-reviewed articles, omitting potentially relevant data published in the grey literature. We were also limited to studies published in English. Publication bias was also a limitation—while we included all available studies that were captured in our search, there may be other relevant technologies that have not been described in peer-reviewed articles.

In conclusion, this review identified several mHealth technologies capable of recording birth outcomes in the delivering room. While none of the technologies were specifically designed for this purpose, the evidence suggests use of digital delivery registers would be both feasible and acceptable to healthcare workers. Few of the tools identified had adequate reporting of interoperability and sustainability, and more research is needed to determine the efficacy and effectiveness of their use.

## Supplementary Material

Reviewer comments

Author's manuscript

## Data Availability

All data relevant to the study are included in the article or uploaded as supplementary information.
