# Supplementary material for: Use of mHealth tools to register birth outcomes in low-income and middle-income countries: a scoping review
Source: BMJ Open. 2022 Oct 12;12(10):e063886. doi: 10.1136/bmjopen-2022-063886 (PMC9562304; doi:10.1136/bmjopen-2022-063886)
Supplement: Supplementary data [file bmjopen-2022-063886supp001.pdf]

APPENDIX

Search strategies:

PubMed:

((parturition* OR childbirth* OR labour*) OR ("Parturition"[Mesh])) AND (((((("Electronic Health Records"[Mesh]) OR ("Mobile Applications"[Mesh] OR "Cell Phone"[Mesh])) OR ("Cell Phone Use"[Mesh] OR "Computers, Handheld"[Mesh])) OR (mobile phone* or mobile* or mhealth* or electronic* or smartphone* or iPhone* or iPad* or tablet computer* or cellphone* or m-health* or ehealth* or e-health* or cell phone* or handheld computer*)) AND ((Africa or Caribbean or "West Indies" or "South America" or "Latin America" or "Central America" or Afghanistan or Albania or Algeria or Angola or "American Samoa" or Armenia or Armenian or Azerbaijan or Bangladesh or Benin or Byelarus or Byelorussian or Belarus or Belorussian or Belorussia or Belize or Bhutan or Bolivia or Bosnia or Herzegovina or Hercegovina or Botswana or Brazil or Brasil or Bulgaria or "Burkina Faso" or "Burkina Fasso" or "Upper Volta" or Burundi or Urundi or Cambodia or "Khmer Republic" or Kampuchea or Cameroon or Cameroons or Cameron or Camerons or "Cape Verde" or "Central African Republic" or Chad or China or Colombia or Comoros or "Comoro Islands" or Comores or Mayotte or Congo or Zaire or "Costa Rica" or "Cote d'Ivoire" or "Ivory Coast" or Cuba or Djibouti or "French Somaliland" or Dominica or "Dominican Republic" or "East Timor" or "East Timur" or "Timor Leste" or Ecuador or Egypt or "United Arab Republic" or "El Salvador" or Eritrea or Ethiopia or Fiji or Gabon or "Gabonese Republic" or Gambia or Gaza or "Georgia Republic" or "Georgian Republic" or Ghana or "Gold Coast" or Grenada or Guatemala or Guinea or Guinea-Bissau or Guam or Guiana or Guyana or Haiti or Honduras or India or Maldives or Indonesia or Iran or Iraq or Jamaica or Jordan or Kazakhstan or Kazakh or Kenya or Kiribati or Korea or Kosovo or Kyrgyzstan or Kirghizia or "Kyrgyz Republic" or Kirghiz or Kirgizstan or "Lao PDR" or Laos or Lebanon or Lesotho or Basutoland or Liberia or Libya or Macedonia or Madagascar or "Malagasy Republic" or Malaysia or Malaya or Malay or Sabah or Sarawak or Malawi or Nyasaland or Mali or "Marshall Islands" or Mauritania or Mauritius or "Agalega Islands" or Mexico or Micronesia or "Middle East" or Moldova or Moldovia or Moldovian or Mongolia or Montenegro or Morocco or Ifni or Mozambique or Myanmar or Myanma or Burma or Namibia or Nepal or "Netherlands Antilles" or Nicaragua or Niger or Nigeria or Pakistan or Palau or Palestine or Panama or "Papua New Guinea" or Paraguay or Peru or Philippines or Philipines or Phillipines or Phillippines or Romania or Rumania or Roumania or Rwanda or Ruanda or "Saint Lucia" or "St Lucia" or "Saint Vincent" or "St Vincent" or Grenadines or Samoa or "Samoan Islands" or "Navigator Island" or "Navigator Islands" or "Sao Tome" or Senegal or Serbia or "Sierra Leone" or Sri Lanka or Ceylon or "Solomon Islands" or Somalia or Sudan or Suriname or Surinam or Swaziland or Syria or Principe or Tajikistan or Tadzhikistan or Tadjikistan or Tadzhik or Tanzania or Thailand or Timor-Leste or Togo or "Togolese Republic" or Tonga or Tunisia or Turkey or Turkmenistan or Turkmen or Tuvalu or Uganda or Ukraine or Uzbekistan or Uzbek or Vanuatu or "New Hebrides" or Vietnam or "Viet Nam" or "West Bank" or Yemen or Zambia or Zimbabwe or Rhodesia or developing countr* or less developed countr* or under developed countr* or underdeveloped countr* or middle income countr* or low income countr* or deprived countr* or underserved countr* or under served countr* or poor countr* or developing nation* or less developed nation* or under developed nation* or underdeveloped nation* or middle income

nation* or low income nation* or deprived nation* or underserved nation* or under served nation* or poor nation* or LMIC or lmics or third world* or lami countr* or transitional countr* or "global health") OR ("Developing Countries"[Mesh]))) AND (("Midwifery"[Mesh]) OR (midwife* or midwives or birth attendant* or doula* or childbirth assistant*)))

Global Health:

((mobile phone* or mobile* or mhealth* or electronic* or smartphone* or iPhone* or iPad* or tablet computer* or cellphone* or m-health* or ehealth* or e-health* or cell phone* or handheld computer).mp. [mp=abstract, title, original title, broad terms, heading words, identifiers, cabicodes] OR (mobile telephones/ OR mobile equipment/)) AND ((midwife* or midwives or birth attendant* or doula* or childbirth assistant*).mp. [mp=abstract, title, original title, broad terms, heading words, identifiers, cabicodes] OR ((traditional birth attendants or midwives).sh)) AND ((childbirth/) OR ((childbirth* or labour*).mp. [mp=abstract, title, original title, broad terms, heading words, identifiers, cabicodes]) AND ((Developing Countries/) OR (Africa or Caribbean or "West Indies" or "South America" or "Latin America" or "Central America" or Afghanistan or Albania or Algeria or Angola or "American Samoa" or Armenia or Armenian or Azerbaijan or Bangladesh or Benin or Byelarus or Byelorussian or Belarus or Belorussian or Belorussia or Belize or Bhutan or Bolivia or Bosnia or Herzegovina or Hercegovina or Botswana or Brazil or Brasil or Bulgaria or "Burkina Faso" or "Burkina Fasso" or "Upper Volta" or Burundi or Urundi or Cambodia or "Khmer Republic" or Kampuchea or Cameroon or Cameroons or Cameron or Camerons or "Cape Verde" or "Central African Republic" or Chad or China or Colombia or Comoros or "Comoro Islands" or Comores or Mayotte or Congo or Zaire or "Costa Rica" or "Cote d'Ivoire" or "Ivory Coast" or Cuba or Djibouti or "French Somaliland" or Dominica or "Dominican Republic" or "East Timor" or "East Timur" or "Timor Leste" or Ecuador or Egypt or "United Arab Republic" or "El Salvador" or Eritrea or Ethiopia or Fiji or Gabon or "Gabonese Republic" or Gambia or Gaza or "Georgia Republic" or "Georgian Republic" or Ghana or "Gold Coast" or Grenada or Guatemala or Guinea or Guinea-Bissau or Guam or Guiana or Guyana or Haiti or Honduras or India or Maldives or Indonesia or Iran or Iraq or Jamaica or Jordan or Kazakhstan or Kazakh or Kenya or Kiribati or Korea or Kosovo or Kyrgyzstan or Kirghizia or "Kyrgyz Republic" or Kirghiz or Kirgizstan or "Lao PDR" or Laos or Lebanon or Lesotho or Basutoland or Liberia or Libya or Macedonia or Madagascar or "Malagasy Republic" or Malaysia or Malaya or Malay or Sabah or Sarawak or Malawi or Nyasaland or Mali or "Marshall Islands" or Mauritania or Mauritius or "Agalega Islands" or Mexico or Micronesia or "Middle East" or Moldova or Moldovia or Moldovian or Mongolia or Montenegro or Morocco or Ifni or Mozambique or Myanmar or Myanma or Burma or Namibia or Nepal or "Netherlands Antilles" or Nicaragua or Niger or Nigeria or Pakistan or Palau or Palestine or Panama or "Papua New Guinea" or Paraguay or Peru or Philippines or Philipines or Phillipines or Phillippines or Romania or Rumania or Roumania or Rwanda or Ruanda or "Saint Lucia" or "St Lucia" or "Saint Vincent" or "St Vincent" or Grenadines or Samoa or "Samoan Islands" or "Navigator Island" or "Navigator Islands" or "Sao Tome" or Senegal or Serbia or "Sierra Leone" or Sri Lanka or Ceylon or "Solomon Islands" or Somalia or Sudan or Suriname or Surinam or Swaziland or Syria or Principe or Tajikistan or Tadzhikistan or Tadjikistan or Tadzhik or Tanzania or Thailand or Timor-Leste or Togo or "Togolese Republic" or Tonga or Tunisia or Turkey or Turkmenistan or Turkmen or Tuvalu or Uganda or Ukraine or Uzbekistan or Uzbek or Vanuatu or "New Hebrides" or Vietnam or "Viet Nam" or "West Bank" or Yemen or Zambia or

Zimbabwe or Rhodesia or developing countr* or less developed countr* or under developed countr* or underdeveloped countr* or middle income countr* or low income countr* or deprived countr* or underserved countr* or under served countr* or poor countr* or developing nation* or less developed nation* or under developed nation* or underdeveloped nation* or middle income nation* or low income nation* or deprived nation* or underserved nation* or under served nation* or poor nation* or LMIC or lmics or third world* or lami countr* or transitional countr* or "global health").mp. [mp=abstract, title, original title, broad terms, heading words, identifiers, cabicodes])

CINAHL:
(mh:("Midwifery") OR (midwife* or midwives or birth attendant* or doula* or childbirth assistant*)) AND (mh:(“Labour”) OR (childbirth* OR labour*)) AND (mh:(“Cellular Phone” OR “Mobile Applications” OR “Electronic Health Records” OR “Computers, Hand-Held” OR “Telemedicine”) OR (mobile phone* or mobile* or mhealth* or electronic* or smartphone* or iPhone* or iPad* or tablet computer* or cellphone* or m-health* or ehealth* or e-health* or cell phone* or handheld computer*)) AND (mh:(“Developing Countries”) OR (Africa or Caribbean or "West Indies" or "South America" or "Latin America" or "Central America" or Afghanistan or Albania or Algeria or Angola or "American Samoa" or Armenia or Armenian or Azerbaijan or Bangladesh or Benin or Byelarus or Byelorussian or Belarus or Belorussian or Belorussia or Belize or Bhutan or Bolivia or Bosnia or Herzegovina or Hercegovina or Botswana or Brazil or Brasil or Bulgaria or "Burkina Faso" or "Burkina Fasso" or "Upper Volta" or Burundi or Urundi or Cambodia or "Khmer Republic" or Kampuchea or Cameroon or Cameroons or Cameron or Camerons or "Cape Verde" or "Central African Republic" or Chad or China or Colombia or Comoros or "Comoro Islands" or Comores or Mayotte or Congo or Zaire or "Costa Rica" or "Cote d'Ivoire" or "Ivory Coast" or Cuba or Djibouti or "French Somaliland" or Dominica or "Dominican Republic" or "East Timor" or "East Timur" or "Timor Leste" or Ecuador or Egypt or "United Arab Republic" or "El Salvador" or Eritrea or Ethiopia or Fiji or Gabon or "Gabonese Republic" or Gambia or Gaza or "Georgia Republic" or "Georgian Republic" or Ghana or "Gold Coast" or Grenada or Guatemala or Guinea or Guinea-Bissau or Guam or Guiana or Guyana or Haiti or Honduras or India or Maldives or Indonesia or Iran or Iraq or Jamaica or Jordan or Kazakhstan or Kazakh or Kenya or Kiribati or Korea or Kosovo or Kyrgyzstan or Kirghizia or "Kyrgyz Republic" or Kirghiz or Kirgizstan or "Lao PDR" or Laos or Lebanon or Lesotho or Basutoland or Liberia or Libya or Macedonia or Madagascar or "Malagasy Republic" or Malaysia or Malaya or Malay or Sabah or Sarawak or Malawi or Nyasaland or Mali or "Marshall Islands" or Mauritania or Mauritius or "Agalega Islands" or Mexico or Micronesia or "Middle East" or Moldova or Moldovia or Moldovian or Mongolia or Montenegro or Morocco or Ifni or Mozambique or Myanmar or Myanma or Burma or Namibia or Nepal or "Netherlands Antilles" or Nicaragua or Niger or Nigeria or Pakistan or Palau or Palestine or Panama or "Papua New Guinea" or Paraguay or Peru or Philippines or Philipines or Phillipines or Phillippines or Romania or Rumania or Roumania or Rwanda or Ruanda or "Saint Lucia" or "St Lucia" or "Saint Vincent" or "St Vincent" or Grenadines or Samoa or "Samoan Islands" or "Navigator Island" or "Navigator Islands" or "Sao Tome" or Senegal or Serbia or "Sierra Leone" or Sri Lanka or Ceylon or "Solomon Islands" or Somalia or Sudan or Suriname or Surinam or Swaziland or Syria or Principe or Tajikistan or Tadzhikistan or Tadjikistan or Tadzhik or Tanzania or Thailand or Timor-Leste or Togo or "Togolese Republic" or Tonga or Tunisia or Turkey or Turkmenistan or Turkmen or Tuvalu or Uganda or Ukraine or Uzbekistan or Uzbek or Vanuatu or "New Hebrides" or Vietnam or "Viet Nam" or "West Bank" or

Yemen or Zambia or Zimbabwe or Rhodesia or developing countr* or less developed countr* or under developed countr* or underdeveloped countr* or middle income countr* or low income countr* or deprived countr* or underserved countr* or under served countr* or poor countr* or developing nation* or less developed nation* or under developed nation* or underdeveloped nation* or middle income nation* or low income nation* or deprived nation* or underserved nation* or under served nation* or poor nation* or LMIC or lmics or third world* or lami countr* or transitional countr* or "global health"))
