# Supplementary material for: Use of mHealth tools to register birth outcomes in low-income and middle-income countries: a scoping review
Source: BMJ Open. 2022 Oct 12;12(10):e063886. doi: 10.1136/bmjopen-2022-063886 (PMC9562304; doi:10.1136/bmjopen-2022-063886)
Supplement: Author's manuscript [file bmjopen-2022-063886.draft_revisions.pdf]

BMJ Open

BMJ Open is committed to open peer review. As part of this commitment we make the peer review history of every article we publish publicly available.

When an article is published we post the peer reviewers' comments and the authors' responses online. We also post the versions of the paper that were used during peer review. These are the versions that the peer review comments apply to.

The versions of the paper that follow are the versions that were submitted during the peer review process. They are not the versions of record or the final published versions. They should not be cited or distributed as the published version of this manuscript.

BMJ Open is an open access journal and the full, final, typeset and author-corrected version of record of the manuscript is available on our site with no access controls, subscription charges or pay-per-view fees (http://bmjopen.bmj.com).

If you have any questions on BMJ Open's open peer review process please email info.bmjopen@bmj.com

BMJ Open

# A scoping review that explores the use and evidence for digital birth registers in Low- and Middle-Income settings



| | |
|---|---|
| Journal: | *BMJ Open* |
| Manuscript ID | bmjopen-2022-063886 |
| Article Type: | Original research |
| Date Submitted by the Author: | 28-Apr-2022 |



| | |
|---|---|
| Complete List of Authors: | Cansdale, Lottie; University of Oxford Medical Sciences Division,<br>Kelly, Gabriella; University of Oxford Medical Sciences Division<br>Khashan, Ali; University College Cork School of Public Health<br>Malata, Address; Malawi University of Science and Technology<br>Kachale, Fannie; Malawi Ministry of Health<br>Lissauer, David ; Malawi-Liverpool-Wellcome Trust Clinical Research Programme; University of Liverpool, Institute of Life Course and Medical Sciences<br>Yosefe, Simeon; Malawi Ministry of Health, Central Monitoring and Evaluation Division<br>Roberts, James; Magee-Women's Research Institute<br>Woodworth, Simon; University College Cork Business School<br>mmbaga, blandina; Kilimanjaro Christian Medical University College, Kilimanjaro Clinical Research Institute<br>Redman, Christopher ; University of Oxford, Nuffield Department of Women’s & Reproductive Health<br>Hirst, Jane Elizabeth; University of Oxford, Nuffield Department of Women's and Reproductive Health |



| | |
|---|---|
| Keywords: | OBSTETRICS, Information technology < BIOTECHNOLOGY & BIOINFORMATICS, Health informatics < BIOTECHNOLOGY & BIOINFORMATICS |
| | |


SCHOLARONE™
Manuscripts

*I, the Submitting Author has the right to grant and does grant on behalf of all authors of the Work (as defined in the below author licence), an exclusive licence and/or a non-exclusive licence for contributions from authors who are: i) UK Crown employees; ii) where BMJ has agreed a CC-BY licence shall apply, and/or iii) in accordance with the terms applicable for US Federal Government officers or employees acting as part of their official duties; on a worldwide, perpetual, irrevocable, royalty-free basis to BMJ Publishing Group Ltd ("BMJ") its licensees and where the relevant Journal is co-owned by BMJ to the co-owners of the Journal, to publish the Work in this journal and any other BMJ products and to exploit all rights, as set out in our licence.*

*The Submitting Author accepts and understands that any supply made under these terms is made by BMJ to the Submitting Author unless you are acting as an employee on behalf of your employer or a postgraduate student of an affiliated institution which is paying any applicable article publishing charge ("APC") for Open Access articles. Where the Submitting Author wishes to make the Work available on an Open Access basis (and intends to pay the relevant APC), the terms of reuse of such Open Access shall be governed by a Creative Commons licence – details of these licences and which Creative Commons licence will apply to this Work are set out in our licence referred to above.*

*Other than as permitted in any relevant BMJ Author's Self Archiving Policies, I confirm this Work has not been accepted for publication elsewhere, is not being considered for publication elsewhere and does not duplicate material already published. I confirm all authors consent to publication of this Work and authorise the granting of this licence.*

For peer review only

# A scoping review that explores the use and evidence for digital birth registers in Low- and Middle-Income settings

**LG Cansdale**[1], G Kelly[1], AS Khashan[2], A Malata[3], F Kachale[4], D Lissauer[5,6], S Yosefe[4], JM Roberts[7], S Woodworth[8], BT Mmbaga[9], C Redman[10], JE Hirst[10]

1. University of Oxford Medical School, UK
2. School of Public Health, University College Cork, Ireland
3. Malawi University of Science and Technology, Malawi
4. Ministry of Health, Malawi
5. Malawi-Liverpool Wellcome Research Programme, Blantyre, Malawi
6. Institute of Life Course and Medical Sciences, University of Liverpool, UK
7. Magee Women's Research Institute, Department of Obstetrics and Gynecology, Epidemiology and Clinical and Translational Research, University of Pittsburgh, USA
8. Cork University Business School, University College Cork, Ireland
9. Kilimanjaro Clinical Research Institute, Kilimanjaro Christian Medical Centre and Kilimanjaro Christian Medical University College, Moshi, Tanzania
10. Nuffield Department of Women's & Reproductive Health, University of Oxford, UK

Corresponding author: LG Cansdale, University of Oxford Medical School, UK. Email: lottie.cansdale@st-hildas.ox.ac.uk.

For peer review only

ABSTRACT

**Objective:** Accurate reporting of birth outcomes in low- and middle-income countries (LMICs) is essential. It has been proposed that conventional paper-based registers could be replaced by mobile (mHealth) tools. mHealth could provide timely data for individual facilities and health departments, as well as capture deliveries occurring outside facilities. This scoping review evaluated what mHealth tools have been reported to record birth outcomes in the delivering room in LMICs and documents their reported advantages and drawbacks.

**Method**: MEDLINE, CINAHL and Global Health were searched for papers where mHealth tools were used by health workers in LMICs to record birth outcomes at delivery. Two independent reviewers screened the papers using pre-determined inclusion and exclusion criteria. Data were extracted, and common themes identified.

**Results**: 640 records were screened, 21 of which met the inclusion criteria, describing 15 different mHealth tools. Studies were conducted in 12 countries in Asia, South America, and Africa. We identified six themes: (1) digital tools for labour monitoring; (2) digital data collection of specific birth outcomes; (3) digital technologies used in community settings; (4) attitudes of health workers; (5) paper vs electronic data collection; and (6) infrastructure, interoperability, and sustainability.

mHealth strategies were acceptable to health workers and enabled greater data completeness compared to paper reporting. Community studies showed data collection using mHealth is feasible. However, in three studies, workers were concerned about the time needed to complete electronic forms, and only three papers considered interoperability with pre-existing health systems.

**Conclusion**: Several mHealth technologies have been reported to collect data at birth, but none are purposed specifically to record birth outcomes at delivery. Use of digital delivery registers appears feasible and acceptable to health workers, but definitive evaluations are lacking. Further research is needed to determine the sustainability of technologies and integration with existing health information systems.


**What is already known on this topic?** Paper-based delivery registers are used to record births across many low- and middle-income countries. These are difficult to store and harder to access for both health professionals and those carrying out data monitoring. With advances in mHealth and digital data collection, we aimed to determine if use of digital births records using mHealth technologies would be both feasible and acceptable.

**What this study adds:** We identified 15 digital technologies capable of recording delivery data at the time of birth, used across both hospital and community settings. Healthcare workers favoured use of mHealth over conventional reporting methods and digital records were more complete compared to paper. However, there were some issues

For peer review only

regarding time taken to input data. Few papers reported the infrastructure required for digital technologies, or considered the sustainability of interventions and interoperability with existing health systems.

**How this study might affect research, practice or policy:** Use of digital births registers in the delivering room is both feasible and would be acceptable to health workers, but there is currently no tool specifically designed for this purpose. Future research needs to consider and report on the sustainability and interoperability of digital technologies.

**Strengths and limitations of this study:**

1. We have used a comprehensive search strategy to explore the literature regarding mHealth and digital birth registers in LMICs.

2. To our knowledge, this is the first review to examine the use of mHealth specifically in the delivering room.

3. We have reported data items using the mERA checklist, which was developed by the WHO to improve reporting of mHealth technologies.

4. Only peer-reviewed articles have been included, meaning relevant data in the grey literature or technologies not reported in peer-reviewed articles may have been omitted.

For peer review only



For peer review only - http://bmjopen.bmj.com/site/about/guidelines.xhtml

# *A scoping review that explores the use and evidence for digital birth registers in Low- and Middle-Income settings*

## *Introduction*

Documentation of birth and perinatal events in low- and middle-income countries (LMICs) and obtaining accurate statistics relating to mortality and morbidity can be challenging. Accurate data are needed for health outcomes monitoring, to identify problems and to track the impact of interventions, as well as to understand the effects of health system shocks, such as COVID-19. In most high-income countries, electronic medical records are used to capture information at birth. This enables rapid monitoring of trends within units, regions and countries, and forms the basis for national surveillance programmes aimed at reducing perinatal and maternal deaths. However, hospital-based electronic record systems are expensive, requiring large investments in computer hardware, networks and ongoing technical and security maintenance and support.

Traditionally, births are recorded around the world in paper-based delivery registers. These are usually large books, completed by birth attendants soon after the birth. Whilst their content and quality can be variable, they are often the only source of data for collation of perinatal health statistics. Paper-based registers have a short memory and, being large, are difficult to store. The information in delivery registers is difficult to access when it is needed for patient care at a later date, for comparing data between institutions or for monitoring trends in care and outcomes over time. Furthermore, non-institutional births may not be recorded at all. LMICs have a high proportion of non-facility births; for example, only 48% of births in Ethiopia, 53% in Bangladesh and 65% in Guatemala take place in healthcare facilities [1]. LMICs are also the countries with the highest maternal and neonatal mortality rates; maternal deaths per 100,000 are estimated at 375 in Uganda, 342 in Kenya and 177 in Indonesia [2]. In other words, those most at risk and in need are those least likely to be documented.

Use of mobile phones is increasing rapidly. One report suggested ownership rates of 80% in 'emerging economies', of which 47% are smartphones [3]. Given the increasing ubiquity of smartphones, their use could

For peer review only



For peer review only - http://bmjopen.bmj.com/site/about/guidelines.xhtml

improve reporting of delivery and birth outcomes. This application is termed mHealth, and is defined by the WHO as 'the use of mobile wireless technologies for health' [4]. mHealth is already being used in low-income settings to register pregnancy and to send reminders of antenatal appointments. One systematic review identified 19 different papers describing 15 mHealth technologies used to document maternal health interventions in LMICs [5]. However, it is unclear at present to what extent mHealth is used to capture birth data at the place of delivery, and whether this approach improves the recording of perinatal outcomes. Hence the purpose of this scoping review is to understand what mobile digital data collection technologies have been evaluated to record births outcomes at the time and place of delivery in LMICs.

## *Methods*

This review was developed in line with the PRISMA and JBI (Joanna Briggs Institute) guidelines for scoping reviews [6, 7], using the PCC mnemonic – population, concept, context. A scoping review gives an overview of the evidence, maps the literature and identifies and analyses current gaps in knowledge. We did not publish a review protocol.

*Population*

LMICs, as defined by the WHO/World Bank[8]. The population of this review comprises healthcare workers recording delivery outcomes, namely midwives, community health workers, doctors and other cadre of skilled birth attendants LMICs.

*Concept*

Our focus is the use of mHealth to capture data at birth, and specifically those technologies present in the place of delivery. Of these many are expected to have wider applications, such as recording of the outcomes of antenatal and postnatal appointments, or progress in labour via an e-partogram. For this scoping review, we have included


For peer review only - http://bmjopen.bmj.com/site/about/guidelines.xhtml

any technology if outcomes at birth were recorded by the birth attendant or community health worker, at the time of, or shortly after, delivery.

*Context*

The context of the review are the places where births occur in LMICs. This includes home and community deliveries, as well as institutional births in health centres, nursing homes, government or private hospitals.

***Search Strategy***

To identify relevant sources, three databases were searched: MEDLINE, CINAHL and GLOBAL HEALTH. The search strategy was created with an experienced librarian and further refined by the authors. The final search strategies for each database can be found in the appendix. The databases were searched for relevant articles up to August 2021. A lower limit for the date was not specified because it was determined by the fact the term 'mHealth' was not widely used before 2000. The final searches were uploaded into EndNote and duplicates were removed by their duplicate detection software.

***Screening and selection of relevant papers.***

Once the citations had been extracted to EndNote, they were uploaded on to Colandr (colandrapp.com), a software that manages citation and full-text screening for reviews. Texts were first screened by abstract, and those that met the inclusion criteria then went on to the full-text screen. Two reviewers (LC and GK) did both citation and full-text screening of articles, and any disputes between reviewers were resolved by a third author (JH).

References of papers included for full-text screening were hand-searched to look for further relevant papers. These hand-picked citations were uploaded to EndNote ad then included in citation screening. Once imported into EndNote duplicates were removed.

***Inclusion and exclusion Criteria***

Eligible studies were conducted in LMICs (defined by the World Bank list of LMICs) [8]. Within each study, the mHealth tool needed to be capable of collecting data regarding birth outcomes at the place of delivery. We

For peer review only

considered studies where the data was collected by nurses/midwives/birth attendants. We limited the search to English language publications only.

We excluded studies where no birth outcomes were reported in the publication; where data were collected using hospital EHR systems on desktop computers; where birth registration data was collected after the delivery or for the purpose of vital registration only; and where data were collected by people other than nurses/midwives/birth attendants. As this was intended as a scoping review of published literature, we excluded non peer-reviewed reports of technologies, such as reports, web pages and media releases.

***Data extraction and synthesis***

An Excel spreadsheet was used for data-extraction, with variables to extract agreed through discussion between the authors. The two reviews (LC and GK) independently charted the data and discussed the results.

We abstracted data on article characteristics, including country of origin, funder, form of mHealth, technology user, reported birth outcomes and technology feasibility and acceptability.

We conducted a narrative synthesis based on our findings. We first mapped studies by geographic location, technology type, primary intended use of technology, accessibility outside the program described (i.e., whether the technology was Open Access or not), and intended user group (hospital/clinic staff or in the community). We then identified the main thematic areas that have been evaluated to date, with the purpose of summarising the current state of the academic literature in this field and identifying gaps for further research. We made particular use of mERA (mHealth Evidence Reporting and Assessment), a checklist which was developed by the WHO to standardise mHealth evidence reporting [9].

***Patient and public involvement***

Neither patients nor public were involved in any aspect of this review.



For peer review only - http://bmjopen.bmj.com/site/about/guidelines.xhtml

## *Results*

### 1. Identification of eligible studies for review

The search strategy is outlined in Figure 1. The search, carried out on 31/07/2021 and updated on 03/02/2022, yielded 640 records– 578 from PubMed, 34 from CINAHL, 28 from Global Health.

*Figure 1: screening and selection of articles*

For peer review only

For peer review only

## 2. Study Characteristics and Themes

The characteristics of each study, in terms of how it was designed, are described in Table 1, together with the six

| Name | Author/s, year/s | Country | Study design[1], N participants | Parto[a] | Outcomes[b] | Comm[c] | Attitudes[d] | paper vs elect[e] | Sustain[f] |
|---|---|---|---|---|---|---|---|---|---|
| 1. ePartogram | | | | | | | | | |
| | Litwin et al, 2018 [10] | Tanzania | Feasibility and acceptability, 23 SBAs | ● (green) | ● (red) | ● (red) | ● (green) | ● (yellow) | ● (yellow) |
| | Sanghvi et al, 2019 [11] | Kenya | Mixed method, quasi-experimental, 110 SBAs | ● (green) | ● (red) | ● (red) | ● (green) | ● (green) | ● (red) |
| 2. E-partograph | | | | | | | | | |
| | Rahman et al, 2019 [12] | Bangladesh | Prospective cross-over study Nurse-midwives (NNS*) | ● (green) | ● (red) | ● (red) | ● (green) | ● (yellow) | ● (yellow) |
| | Tadesse et al, 2019 [13] | Ethiopia | Cross-sectional study with questionnaire 462 obstetric care providers | ● (green) | ● (red) | ● (red) | ● (green) | ● (yellow) | ● (yellow) |
| 3. mLabour | | | | | | | | | |
| | Schweers et al, 2016 [14] | India | Design and preliminary evaluation 12 nurses | ● (green) | ● (red) | ● (red) | ● (green) | ● (yellow) | ● (red) |
| 4. prasavGraph | | | | | | | | | |
| | Singh et al, 2016 [15] | India | Development and pilot Doctors and student nurses (NNS) | ● (green) | ● (red) | ● (red) | ● (red) | ● (red) | ● (yellow) |
| 5. ASMAN | | | | | | | | | |
| | Usmanova et al 2020 [16] | India | Qualitative study 44 stakeholders | ● (green) | ● (red) | ● (yellow) | ● (green) | ● (yellow) | ● (green) |
| | Usmanova et al 2021 [17] | India | Secondary analysis of programme data (NNS) | ● (green) | ● (red) | ● (red) | ● (red) | ● (red) | ● (red) |

specific themes we identified. The themes are elaborated in more detail in sections 3-6 below. Table 2 describes the technical aspects and content of interventions.

Table 1: study characteristics and themes

For peer review only

| | | | | | | | | |
|---|---|---|---|---|---|---|---|---|
| **6. PPH reporting** | | | | | | | | |
| Andreatta et al, 2011 [18] | Ghana | Evaluation of technology<br>10 BAs | 🔴 | 🟢 | 🟢 | 🔴 | 🔴 | 🔴 |
| **7. Mobile Delivery Timer** | | | | | | | | |
| Somannavar et al, 2015 [19] | India | Development and evaluation<br>46 BAs | 🔴 | 🟢 | 🔴 | 🟢 | 🔴 | 🟡 |
| **8. Birth defects surveillance system** | | | | | | | | |
| Mumpe-Mwanja et al, 2019 [20] | Uganda | Surveillance study<br>Midwives (NNS) | 🔴 | 🟢 | 🔴 | 🔴 | 🔴 | 🔴 |
| **9. RapidSMS-MCH** | | | | | | | | |
| Ngabo et al, 2012 [21] | Rwanda | Design and implementation<br>432 CHW | 🔴 | 🔴 | 🟢 | 🔴 | 🔴 | 🟢 |
| Musabyimana et al, 2018 [22] | Rwanda | Qualitative study: IDIs , FGDs<br>93 participants including CHW | 🔴 | 🔴 | 🟢 | 🟢 | 🔴 | 🟢 |
| **10. Data collection** | | | | | | | | |
| Little et al, 2013 [23] | Ethiopia | Development and evaluation<br>12 midwives, 20 health extension workers | 🔴 | 🔴 | 🟢 | 🟢 | 🔴 | 🟢 |
| Medhanyie et al, 2015 [24] | Ethiopia | Semi-structured questionnaire<br>23 midwives and health extension workers | 🔴 | 🔴 | 🟢 | 🟢 | 🟢 | 🟢 |
| Medhanyie et al, 2017 [25] | Ethiopia | Comparative cross-sectional study<br>25 midwives and health extension workers | 🔴 | 🔴 | 🟢 | 🔴 | 🟢 | 🔴 |
| **11. Perinatal Monitoring App** | | | | | | | | |
| Stroux et al 2016 [26] | Guatemala | Prototype testing<br>9 healthcare workers | 🔴 | 🔴 | 🟢 | 🟡 | 🔴 | 🟢 |
| Martinez et al, 2017 [27] | Guatemala | Agile development<br>25 midwives | 🔴 | 🔴 | 🟢 | 🟡 | 🔴 | 🟢 |
| Martinez et al, | Guate | Randomised control trial | 🔴 | 🔴 | 🟢 | 🔴 | 🔴 | 🔴 |

For peer review only

| | | | | | | | | | |
|---|---|---|---|---|---|---|---|---|---|
| | 2018 [28] | mala | 44 TBAs | | | | | | |
| **12. Hayat** | | | | | | | | | |
| | Zaidi et al, 2020 [29] | Pakistan and Afghanistan | Qualitative exploratory study 54 lady health workers, 42 CHW | ● (not described) | ● (not described) | ● (described) | ● (described) | ● (partially described) | ● (described) |
| **13. Maternal referral mobile system** | | | | | | | | | |
| | Indriani et al, 2020 Indonesia [30] | | Development of technology Midwives (NNS) | ● (not described) | ● (not described) | ● (described) | ● (partially described) | ● (partially described) | ● (partially described) |

Key:

● = not described ● = described ● = partially described

[a]Partogram or labour monitoring tool

[b]Other birth outcomes described

[c]Community use

[d]Attitudes of health workers reported

[e]Comparison of paper and electronic data capture reported

[f]Sustainability issues discussed

NNS= number not stated



For peer review only - http://bmjopen.bmj.com/site/about/guidelines.xhtml

For peer review only

| Name | Platform | Interoperability/ HIS context | Intervention content |
|---|---|---|---|
| 1. **ePartogram** | Android tablet App | Data accessible at referral facilities | Clinical decision-support tool, with automatic graphing of data and visual and auditory reminders and alerts |
| 2. **e-partograph** | Android Smartphone or tablet App | Partograph data monitored remotely | Based on WHO partograph<br>Gives alerts to maternal or foetal anomaly |
| 3. **mLabour** | Mobile App | NS | Mobile partograph and labour management tool to register patients, record measurements and record delivery |
| 4. **prasavGraph** | Android smartphone or tablet App | Birth registration with municipal authorities | Partograph with digital record keeping |
| 5. **ASMAN** | NS | Integrated into government databases | Digitised case sheet with e-partograph, dashboards and reports, E-learning content, Safe Delivery App, case-based game, remote support centre |
| 6. **PPH Monitoring** | SMS based | NS | SMS protocol for reporting of postpartum haemorrhage |
| 7. **Mobile Delivery Timer** | Android smartphone App | NS | Application with voice recording to verbally mark crowning, birth, crying or bag mask ventilation of baby |
| 8. **Birth defect surveillance** | Android tablet App | NS | Baby examined, defects photographed, and surveillance data collected in tablet |
| 9. **RapidSMS** | Open-source App | Automatic notification to ambulance service | SMS platform that reminds CHWs of appointments, facilitates communication between CHWs and health facilities and collects data around specific maternal and child health events |
| 10. **Data collection tool** | Smartphone App | Local health authority | Smartphone application with data collection protocols for different maternal health events, including delivery. Dashboard and mobile scorecard to track patients and performance indicators |
| 11. **Perinatal** | Android | Open MRS | Perinatal Monitoring App with peripheral devices, |

| | | | |
|---|---|---|---|
| **Monitoring System** | smartphone App | | collection of patient demographics, decision making support and communication between BAs and medical team |
| **12. Hayat** | Smartphone App | Local health authority | Mobile application to digitalise record keeping |
| **13. Maternal referral Mobile System** | Smartphone App | Data available at referral centre | App to aid referral of maternity patients between primary health centres and the hospital |

Table 2: Technical aspect and intervention content

HIS= Health information system;
NS= Not stated. CHW= Community health worker

For peer review only

We identified 15 different mHealth technologies, across 12 different countries; 7 in Africa, 7 in Asia and 1 in South America. A variety of health workers were end-users of the technologies, including midwives, nurses, traditional and skilled birth attendants (TBAs and SBAs), and community volunteers.

Six technologies were described in more than one paper. The ePartogram and E-partograph were studied in two different settings: Tanzania and Kenya for the ePartogram [10, 11], and Bangladesh and Ethiopia for the E-partograph [12, 13]. The further studies on the other four technologies (RapidSMS, the data collection tool, perinatal monitoring app and ASMAN) were conducted in the same setting as the first published paper.

A variety of methods were used to assess the technologies, with the majority being early phase development and pilot work. Only one study reported results of a randomised control trial [28]. Three studies were preclinical [15, 26, 30]. Sample sizes also varied between studies, with the largest being 462 [13] and smallest 9 [26]. Five studies did not document the number of health workers using the technology [12, 15, 17, 20, 30].

### 3. Roles for the digital tools

We identified three main roles for digital tools at birth: (1) labour monitoring; (2) recording of a specific birth outcome; (3) tools for use in the community. They were mainly concerned with: (1) attitudes of health workers to


For peer review only - http://bmjopen.bmj.com/site/about/guidelines.xhtml

digital technologies; (2) comparison of paper and digital data collection; and (3) sustainability, interoperability, and infrastructure for mHealth interventions.

### 3.1. Digital tools for labour monitoring

Eight of the papers described tools that could monitor labour, all based on the WHO partograph [10-17] and shown in Table 3. Whilst the primary purpose of the partograph is to monitor labour these technologies had additional capabilities to record birth data, often to assess the impact of labour monitoring on birth outcomes.

**Table 3: digital tools for labour monitoring**

| Technology | Papers |
|---|---|
| ePartogram | [10, 11] |
| e-Partograph | [12, 13] |
| mLabour | [14] |
| prasavGraph | [15] |
| ASMAN | [16, 17] |

Several electronic partographs have been described. The ePartogram was developed by John Hopkins University and their NGO arm, Jhpiego. Study of its use in Tanzania showed SBAs were comfortable registering clients and entering data into the application and had a preference for the ePartogram over paper versions[10]. This was replicated in a study in Kenya, where it was also found to improve birth outcomes compared to paper partograph use [11]. Improvements in birth outcomes was also seen in studies investigating the e-Partograph in Bangladesh [12].. However, a separate study of the E-partograph in Ethiopia found only 46% of care providers would be willing to use a mobile-based E-partograph, despite 65.8% of caregivers reporting they wished to use a partograph routinely [13].

Three other technologies were identified that had a digital partograph as part of their workflow. prasavGraph is an app incorporating a partograph with other data collection, including the baby's sex, weight and condition at birth, reported in preclinical phase in New Delhi [15]. mLabour also incorporated a partograph into their labour management tool; nurses using it reported being more punctual with data entry and found the tablet form lighter and easier to record data in compared to multiple paper registers [14]. This feedback was echoed with the ASMAN application, which consists of a digital case sheet for patients, partograph, dashboard for health facilities and E-

For peer review only

learning content, and was used in both district hospitals and community health centres [16]. Analysis of application data showed high rates of data collection for delivery in the app, at 93.7% [17].

### 3.2. Digital collection for specific birth outcomes

Three technologies were designed to report a specific outcome, but included delivery details as part of the workflow. A system for community midwives to report postpartum haemorrhage (PPH) was designed in Rwanda, using mobile phone handsets and text protocols [18]. As well as PPH, data were collected on maternal and neonatal deaths. Ten TBAs used the technology and successfully reported 425 births and 13 incidents of PPH.

The other two studies collected data collected on tablets. The Mobile Delivery Timer was designed to measure the time between delivery and either the baby crying or initiation of bag mask ventilation, as part of a larger 'Helping Babies' Breath' study [19]. This recorded the time of delivery as well as the woman's ID, newborn status and any malformations. 90% of birth attendants found recording data in the mobile phone either 'moderately easy to use' or 'very easy to use'. Android tablets were used in a tool to detect birth defect prevalence in Uganda [20]. All births were examined by a midwife as soon as feasible and data collected on forms designed using Open Data Kit (ODK), including both pictures of any major defects as well as quantitative data documenting maternal age, birth outcome, newborn sex and mode of delivery. The system was able to detect a birth defect prevalence of 66 per 10,000 births, but the authors noted there was no population-based data for comparison.

### 3.3. Digital tools for use in the community

Six of the tools were designed specifically to be used in the community, either by trained midwives or lay or volunteer health workers.

Two technologies used mobile phones and SMS protocols to capture community data. The PPH reporting system is one example [18], as is the RapidSMS-MCH system in Rwanda [21]. In Rwanda, volunteer community health workers were given mobile phones with eight SMS forms for reporting data, including one for delivery. The SMS

For peer review only

was sent to a central server and could alert an ambulance in case of emergency. The technology was scaled up nationwide in 2013, and a further study found it was accepted by both health workers and the local community [22].

A further three technologies used smartphones to record data collected by community health workers. An app was designed for data collection by midwives and health extension workers (HEWs) in Ethiopia, containing eight data collection forms for different perinatal events [23]. It also contained a scorecard and analytics dashboard to allow data monitoring by local health bureaus. The app was adapted for use in the community by using the local calendar and local language. Analysis of the records submitted found that most delivery protocols were submitted by midwives rather than HEWs, reflecting local guidance that HEWs should not assist with deliveries [24]. A similar format was seen with the Hayat app in Afghanistan and Pakistan; it was designed to digitalise data entry, and also contained a dashboard to visualise reports generated by patient encounters [29]. Community health workers were able to successfully use the app but highlighted issues such as the app not using the local language. The perinatal monitoring tool used in Guatemala was adapted for community use by having audio and visual instructions and data collection through audio recordings, to be inclusive of illiterate and technology-naïve end-users [26, 27]. Similarly, to the other technologies, it collects data based on antenatal, postnatal and intrapartum encounters, combined with recording of vital signs. Once assessment is complete, the data is uploaded to the patient's OpenMRS electronic medical record. An initial feasibility study showed use was feasible among traditional birth attendants, and later randomised control testing showed that use of the app was associated with improved detection of pregnancy complications and a higher rate of referrals to medical facilities [28].

Several of the tools were designed to aid communication between health professionals. A mobile referral system was designed in Indonesia, allowing midwives to relay information about patient condition and neonatal outcomes to the referral hospital [30]. This was a feature of other platforms: the perinatal monitoring system alerted the local health facility if a complication was detected [28], and RapidSMS could automatically forward ambulance requests in an emergency [21]. ASMAN also facilitated communication, through access to a remote support centre staffed by health personnel [16].

For peer review only

### 4. Attitudes of health workers towards digital collection of birth outcomes

Ten studies explored the attitudes of health workers towards digital tools. Seven used focus groups or in-depth interviews [10, 11, 14, 16, 22, 23, 29] and three used questionnaires [13, 19, 24]. All but one of the studies reported that the technology was well accepted and preferred to conventional methods of data collection.

Four studies were specifically designed to explore the preferences of health workers [13, 16, 22, 24]. Generally they showed positive attitudes towards digital data collection; one study in Ethiopia found 87% of health workers perceived it to be useful [24], and that ownership of the mobile phones was a motivating factor for using the technology for 91.3%. Mobile phones were also given to health workers as part of the RapidSMS project [22]. Whilst the technology was perceived to improve birth outcomes and was well accepted by both health workers and the community, the increased reporting requirement created by the technology had reduced motivation of the volunteer CHWs, and some wanted financial compensation for the extra work. Only one study used a validated tool to study health worker preferences; the Technology Acceptance Model, which explores perceived ease of use and usefulness of a technology, was used to evaluate the ASMAN application [16]. The application was reported to be easy to use and less error prone compared to paper, and improved clinical documentation by prompting users to ask for additional information as necessary.

Only one study showed a negative attitude towards a digital technology [13]. Analysis of attitudes towards the e-Partograph showed that whilst 99.6% of workers owned a mobile phone, only 46% were willing to use a mobile-based e-Partograph; however, this did not specify whether they were unwilling to use the app on their personal phone or unwilling to use the e-Partograph in general. 58.9% of those surveyed had a favourable attitude towards the partograph, and this was associated with willingness to use the e-Partograph, so the authors concluded that the results may be due to negative attitudes towards the partograph in general rather than an electronic tool specifically.

In six studies the time taken to use digital technologies was mentioned by health workers. In three, digital data collection was perceived to be a timesaver [10, 16, 29]. However, those using the ePartogram were concerned

For peer review only

about achieving timely data entry in high volume sites or with multiple clients [11], and 78.3% of those using the Ethiopian data collection tool cited time taken as a reason for not using forms all the time [24]. With ASMAN, whilst facilities using the application alone to collect data found it reduced reporting time, some facilities required data collection electronically and on paper for verification, audit or in case of paper issues, which increased workload temporarily [16].

## 5. Comparisons of paper and electronic tools

mHealth tools were formally compared to the current standard of reporting, paper records, in three of the studies [11, 12, 25]. This was either comparing preferences for paper or electronic data collection or comparing completeness of records.

The current WHO partograph is a paper version, so studies using an electronic version often used this as a comparison. One cross-over study comparing the E-partograph to the paper version found higher user rates during the electronic phase in both sites [12]. Another study found that all measurements except foetal heart rate and contractions were recorded more frequently in the ePartogram compared to paper [11]. However, SBAs found that it was more difficult to correct errors in data entry using the ePartogram, which was not an issue with the paper charts.

Similar findings were seen with the  data collection tool in Ethiopia [25]. Comparing the completeness and accuracy of patient data collection on electronic forms compared to paper, they found overall completeness was 8% higher in electronic records compared to paper. Whilst there was no difference for parameters such as patient name or age, for newborn weight recording was 20.5% higher in electronic forms. The authors speculated this could be because the electronic forms required a response to be accepted. However, they also found some workers lacked the apparatus to measure some parameters, such as scales for recording newborn weight, and had thus been discouraged from using electronic forms to record data due to mandatory reporting requirements, meaning they were more likely to use paper and reduce the completeness of paper records. The study reported that missing data on paper forms was also due lack of physical space on paper sheets. Both formats had a small error rate (paper



For peer review only - http://bmjopen.bmj.com/site/about/guidelines.xhtml

1.1% and electronic 2.8%), and the majority of errors in electronic forms were in spelling of the patient's name, which was attributed to using the smartphone keyboard.

### 6. Infrastructure, interoperability and sustainability

Few papers included information about the infrastructure, necessary for mHealth technology. Some described the hardware given to study participants – in Ethiopia, workers were given second-hand HTC Hero smartphones, solar lamp and phone chargers [23]. They reported that of the 23 health posts in the area, only 48.9% had GPRS at the time, with only health posts in areas of connectivity being chosen for the project. They also monitored monthly phone expenditure of HEWs on mobile cards, finding that workers were using their phones for personal use as well as for submitting protocol forms. Network connectivity varied across the studies, with some reporting 3G being available in all facilities [10] , whereas those using the Hayat app cited a lack of connectivity as a barrier to uploading data [29]. The Hayat app had also GPS capabilities, so that health workers could be tracked during outreach visits; however, workers had to travel to the central offices once per week to upload data to the central server, which was also the case with the perinatal monitoring system in Guatemala [26].

Four papers reported capability to communicate with pre-existing health information systems (HIS) or upload data to electronic health/medical records (EHR/EMR) [16, 23, 26, 29] The perinatal monitoring app in Guatemala has been used in the community and is able to upload data to the OpenMRS EMR once assessment is complete [26]. However, in the randomised control trial of the application, this feature was not mentioned [28]. The data collection project in Ethiopia discussed uploading their data to EMRs but stated that improvements in patient identification would be needed for this to be successful [23]. However, the Ethiopian data collection app, the Hayat app [29], and the ASMAN package [16] all created a dashboard from data collection reports submitted, which allowed local health bureaus to view the data. Beside connecting to a HIS, prasavGraph reported that their data could be used to record births with municipal authorities, but this has not progressed beyond prototype testing [15].

Table 4 contains a summary of user feedback, contextual adaptability and limitations for delivery at scale. Not all the studies considered sustainability of the intervention, or the feasibility of scaling technology up. One exception


For peer review only - http://bmjopen.bmj.com/site/about/guidelines.xhtml

is RapidSMS-MCH, which, following the initial study, was scaled up across Rwanda to all CHWs. Sustainability was also discussed in a description of the ASMAN package [16]. They detailed a transition plan to move past the pilot phase, which comprised master trainers to those who instructed front line users of the technology, transfer of the application to states' servers and integration of the ASMAN data fields into existing government databases.

Table 4: Limitations and contextual adaptability

For peer review only



For peer review only - http://bmjopen.bmj.com/site/about/guidelines.xhtml

| Name | User feedback | Limitations for delivery at scale | Contextual adaptability |
|---|---|---|---|
| **1. ePartogram** | | | |
| | • Improved decision making and actions<br>• Better organisation of patient records<br>• Data entry was simple and user-friendly<br>• Improved real-time data recording | • Concerns about time for data entry in high-volume labour wards | Feedback session with SBAs done as part of development process |
| **2. e-partograph** | | | |
| | • 44% willing to use<br>• 55% had good knowledge of partograph<br>• 58.9% favourable attitudes [13] | • Obstetric care providers less willing to use | NS |
| **3. mLabour** | | | |
| | • Users preferred tablet format to paper and liked automatic graphing<br>• Requests for additional functionality, e.g. tracking medications | NS | Some features of app customised for context of specific hospital, not wider use |
| **4. prasavGraph** | | | |
| | NS | NS | Designed for use in settings with poor network connection |
| **5. ASMAN** | | | |
| | • Technology was easy to use + less time consuming than conventional methods<br>• Perceived improvement in job performance and documentation of outcomes | • Tablets freezing and issues with internet connectivity<br>• Staff shortages and high caseloads prevented all fields being completed [16] | E-learning written in both English and Hindi with both audio, visual and readable formats |
| **6. PPH Monitoring** | | | |
| | NS | • Poor network and power outages<br>• Lack of remuneration | Training given in English and native language |
| **7. Mobile Delivery Timer** | | | |
| | • 70% felt app beneficial<br>• 90% found easy to use | NS | Android operating system chosen as functions on low-cost hardware |
| **8. Birth defect surveillance** | | | |
| | NS | • System does not capture babies born outside of urban facilities | NS |
| **9. RapidSMS** | | | |
| | • CHWs reported being more pro- | • High initial cost | Mobile phones provided to CHWs |



For peer review only - http://bmjopen.bmj.com/site/about/guidelines.xhtml

| | | |
|---|---|---|
| • active in finding and following up pregnant women<br>• Perception helpful to reduce maternal mortality<br>• CHWs feeling financial compensation for work required as data collection increased workload | • Phone charging problems<br>• Lack of medical equipment prevents all data being collected | to reduce their costs |
| **10. Data collection tool** | | |
| • 91.3% used the smartphone provided as their primary phone<br>• 87% found electronic forms helpful; 52.2% thought they were comprehensive<br>• All would use electronic forms if they were to replace paper forms | • Costs of covering health worker's airtime on phones would limit large-scale implementation<br>• Increased error rate in electronic records compared to paper | Health workers were provided with smartphone and given solar lamp to charge, since many had no electricity supply |
| **11. Perinatal Monitoring System** | | |
| • System was easy to operate with potential benefit | • Some staff unfamiliar with the technology<br>• Ultrasound recordings low quality and corrupted when analysed<br>• Loss of visit summary data | User interface used audio and visual instructions and data collection methods instead of written to adapt to illiterate users |
| **12. Hayat** | | |
| • Use of app was feasible<br>• Improved efficiency of data recording and reduced need to carry multiple registers<br>• CHWs frustrated at being unable to edit entries | • CHWs needed to travel long distances each week to sync app data with central server | NS |
| **13. Maternal referral Mobile System** | | |
| NS | • Bugs in application | The authors conducted focus group interviews with stakeholders before designing application |

NS= Not stated

CHW= Community Health Worker

For peer review only

Most of the technologies used Android hardware, which is widely available in LMICs and is relatively low cost; one study cited its use as a way to increase sustainability [19]. The sustainability of the perinatal monitoring system in Guatemala was assessed in relation to its cost which was less than $50 for the equipment, while its software was open source [25]. The US engineers involved in the development of the app also spent time training engineers in Guatemala to be able to deal with software and hardware issues relating to the technology, which also increases sustainability [27]. Issues in scaling up mHealth use were highlighted in the Ethiopian study where workers were given cell phones; they said that the cost of airtime was high owing to workers using phones for personal and study purposes. They suggested that the intervention would be more sustainable if a mechanism could be found either to provide free airtime for uploading protocols or to restrict airtime to study protocols [24]. One further project cited end-user feedback and involvement in the development process as an important part of ensuring sustainability but did not clarify what this involved [29].

## *Discussion*

We identified 20 papers describing 15 mHealth technologies that could collect data in the obstetric delivery room. Studies showed that data collection at delivery is both feasible and acceptable to healthcare providers. Our analysis identified that technologies were designed for three primary purposes: labour monitoring, collection of a specific birth outcome and data collection by community workers. Whilst no technology was specifically designed to record birth outcomes in the delivering room, our findings suggest that use of such a tool would be feasible and acceptable.

All but one of the studies found that digital data collection was preferred to paper methods. Digital technologies were reported to be easy to use [16, 19], user-friendly [11] and perceived to improve worker efficiency [29] and birth outcomes [22]. Health workers also preferred the digital format compared to paper methods, noting them to be less bulky to carry and easier to access [14, 29], suggesting that replacement of paper-based records with digital registers is feasible. It was also reported that digital collection could improve the number of data items reported, by incorporating mandatory data entry fields [16, 22].

For peer review only



For peer review only - http://bmjopen.bmj.com/site/about/guidelines.xhtml

We found that the time taken to input data can be a barrier. In some studies, this was due to double data entry, with both digital and paper collection of data, rather than being due to the tool itself [16, 24]. However, we also found evidence that health workers were concerned about time to input data [23], particularly in facilities caring for large numbers of clients [11]. Digital tools can mandate reporting of more items, and whilst this is useful for data collection and research, it can become more time-consuming for health workers and reduce the motivation [22]. An mHealth application that did not meet criteria for inclusion, mClinic, sought end-user feedback on items for data reporting, finding that users only wanted items that would be included in reports and did not want free-text for reporting of subjective data [31]. Clearly, end-user involvement in designing data collection protocols is important to ensure that each data item is perceived to be useful for health worker's practice.

We found that there are several gaps in reporting of interventions in the published literature. One of the items in the mERA checklist, developed by the WHO, is 'Interoperability/Health information systems (HIS) context', which involves 'describing how mHealth intervention can integrate into existing health information systems' [9]. Our review found few papers describing how the mHealth tool would integrate with the existing HIS; only one appeared to upload data directly to patient's medical records, the perinatal monitoring app [26]. Another item in the mERA checklist is 'Cost Assessment', and again only the perinatal monitoring app mentioned the cost of the hardware required to run the technology [26]. Consideration of both cost and interoperability are crucial for the sustainability [32], so the lack of reporting in the literature highlights the need for both standardised reporting of interventions and for authors to consider these factors when designing and testing new technology.

We also identified few tools capable of recording births in both facilities and in the community. Whilst several of the community-based technologies could communicate data to facilities, only the Ethiopian data collection tool was used to collect data by both health workers in the community and midwives in health centres [23]. Since the proportion of non-facility births in many LMICs is high [1], a digital birth register would need to be designed for use in both the community and facilities to ensure maximum coverage of births.

To our knowledge, no other review has considered the use of mHealth specifically in the delivering room, or its potential to replace paper-based delivery registers. However, our review is in agreement with other reviews that



For peer review only - http://bmjopen.bmj.com/site/about/guidelines.xhtml

there is a lack of high-quality evidence and robust study design to determine the efficacy and effectiveness of mHealth interventions in maternal health care [5, 33, 34]. Of the papers we identified, only one was a randomised-control trial, and of the 15 technologies only six were evaluated by more than one study. With regards to the purpose of evaluation, only two papers formally assessed the accuracy of digital data collection compared to paper methods [11, 25], and only six papers explored the time needed for digital input relative to paper recording [10, 11, 16, 24, 29] . The lack of quality evidence creates a barrier to scaling up technologies – in our review, only RapidSMS was being delivered at scale [22] – and emphasises the need for pilot studies to be followed-up with studies that investigate scalability [32].

Our own review has its strengths and limitations. Whilst we used a comprehensive search strategy, we only included peer-reviewed articles, omitting potentially relevant data published in the grey literature. We were also limited to papers published in English. Publication bias was also a limitation – whilst we included all available studies that were captured in our search, there may be other relevant technologies that have not been described in peer-reviewed articles.

In conclusion, this review identified several mHealth technologies capable of recording birth outcomes in the delivering room. Whilst none of the technologies were specifically designed for this purpose, the evidence suggests use of digital delivery registers would be both feasible and acceptable to health workers. Few of the tools identified had adequate reporting of interoperability and sustainability, and more research is needed to determine the efficacy and effectiveness of their use.

***ACKNOWLEDGEMENTS***

We are grateful to librarian N. Thomas for helping build the initial search strategy.

***CONTRIBUTORSHIP STATEMENT***

JEH, CR, AK, AM, DL, FC conceived the review idea. LGC carried out the search and LGC and GK screened and selected articles. LGC wrote the manuscript with input from all authors. All authors approved the manuscript prior to submission.

For peer review only

### *DATA SHARING STATEMENT*

All data are included in the manuscript.


### *FUNDING*

There was no specific funding for this project. JEH is funded by a UKRI Future Leaders Fellowship.


### *ETHICS APPROVAL*

This study did not involve human or animal subjects. Ethics approval is not applicable for this article.

### *COMPETING INTERESTS STATMEENT*

The authors declare that there were no conflicts of interest.

### *AUTHOR REFLEXIVITY STATEMENT*

The original concept for this systematic review was co-developed equally by the authors from high and low-income country settings to address an identified priority from the authors from Tanzania and Malawi. The protocol was jointly developed and the search strategy and data extraction were performed by HIC researchers, with support from LIC researchers. The LIC researchers contributed to interpretation of themes and ensuring contextual relevance. All authors read and approved the final version of the manuscript. The first (LGC) and last author (JEH) are both HIC authors, as the first author is a student who has been supervised by the senior author.

### *APPENDIX*

The search strategies can be found in the appendix.

### *REFERENCES*


1. UNICEF, *Maternal and Newborn Health Coverage Database* UNICEF, Editor. 2021.


For peer review only



For peer review only - http://bmjopen.bmj.com/site/about/guidelines.xhtml

2. WHO, U., UNFPA, World Bank Group and UNPD *Trends in estimates of maternal mortality ratio (MMR; maternal deaths per 100,000 live births) maternal deaths and lifetime risk of maternal death, 2000-2017* 2021: https://data.unicef.org/topic/maternal-health/maternal-mortality/.
3. Centre, P.R., *Smartphone Ownership Is Growing Rapidly Around the World, but Not Always Equally* 2019.
4. WHO, *mHealth: Use of appropriate digital technologies for public health* 2018.
5. Colaci, D., S. Chaudhri, and A. Vasan, *mHealth Interventions in Low-Income Countries to Address Maternal Health: A Systematic Review.* Ann Glob Health, 2016. **82**(5): p. 922-935.
6. Tricco, A.C.e.a., *PRISMA Extension for Scoping Reviews (PRISMA-ScR): Checklist and Explanation.* Annals of Internal Medicine, 2018. **169**(7): p. 467-473.
7. Peters, M.G., C; et al *Chapter 11: Scoping Reviews* in *JBI Manual for Evidence Synthesis* E.M. Aromataris, Z, Editor. 2020.
8. Bank, T.W. *Low & middle income* 2021 [cited 2021 21/12/2021]; Available from: https://data.worldbank.org/income-level/low-and-middle-income.
9. Agarwal, S., et al., *Guidelines for reporting of health interventions using mobile phones: mobile health (mHealth) evidence reporting and assessment (mERA) checklist.* BMJ, 2016. **352**: p. i1174.
10. Litwin, L.E., et al., *Use of an electronic Partograph: feasibility and acceptability study in Zanzibar, Tanzania.* BMC Pregnancy Childbirth, 2018. **18**(1): p. 147.
11. Sanghvi, H., et al., *Effectiveness of an Electronic Partogram: A Mixed-Method, Quasi-Experimental Study Among Skilled Birth Attendants in Kenya.* Global Health: Science and Practice, 2019. **7**(4): p. 521-539.
12. Rahman, A., et al., *Feasibility and effectiveness of electronic vs. paper partograph on improving birth outcomes: A prospective crossover study design.* PLoS One, 2019. **14**(10): p. e0222314.
13. Tadesse, Y., et al., *Willingness to Use Mobile based e-Partograph and Associated Factors among Care Providers in North Gondar Zone, Northwest Ethiopia.* Online journal of public health informatics, 2019. **11**(2): p. e10-e10.
14. Schweers, J., et al., *mLabour: Design and Evaluation of a Mobile Partograph and Labor Ward Management Application.* Procedia engineering, 2016. **159**: p. 35-43.
15. Singh, S., et al. *prasavGraph: An Android-based e-Partograph.* in *2016 International Conference on Systems in Medicine and Biology (ICSMB).* 2016.
16. Usmanova, G., et al., *Acceptability and Barriers to Use of the ASMAN Provider-Facing Electronic Platform for Peripartum Care in Public Facilities in Madhya Pradesh and Rajasthan, India: A Qualitative Study Using the Technology Acceptance Model-3.* International journal of environmental research and public health, 2020. **17**(22): p. 8333.
17. Usmanova, G., et al., *The role of digital clinical decision support tool in improving quality of intrapartum and postpartum care: experiences from two states of India.* BMC Pregnancy & Childbirth, 2021. **21**(1): p. 1-12.
18. Andreatta, P., et al., *Using cell phones to collect postpartum hemorrhage outcome data in rural Ghana.* Int J Gynaecol Obstet, 2011. **113**(2): p. 148-51.
19. Somannavar, M.S., et al., *Evaluating time between birth to cry or bag and mask ventilation using mobile delivery room timers in India: the NICHD Global Network's Helping Babies Breathe Trial.* BMC Pediatrics, 2015. **15**(93).
20. Mumpe-Mwanja, D., et al., *A hospital-based birth defects surveillance system in Kampala, Uganda.* BMC Pregnancy Childbirth, 2019. **19**(1): p. 372.
21. Ngabo, F., et al., *Designing and Implementing an Innovative SMS-based alert system (RapidSMS-MCH) to monitor pregnancy and reduce maternal and child deaths in Rwanda.* Pan Afr Med J, 2012. **13**: p. 31.
22. Musabyimana, A., et al., *Assessing the perspectives of users and beneficiaries of a community health worker mHealth tracking system for mothers and children in Rwanda.* PLoS One, 2018. **13**(6): p. e0198725.
23. Little, A., et al., *Meeting Community Health Worker Needs for Maternal Health Care Service Delivery Using Appropriate Mobile Technologies in Ethiopia.* PLOS ONE, 2013. **8**(10): p. e77563.
24. Medhanyie, A.A., et al., *Health workers' experiences, barriers, preferences and motivating factors in using mHealth forms in Ethiopia.* Human Resources for Health, 2015. **13**(1): p. 2.
25. Medhanyie, A.A., et al., *Quality of routine health data collected by health workers using smartphone at primary health care in Ethiopia.* International Journal of Medical Informatics, 2017. **101**: p. 9-14.


For peer review only



For peer review only - http://bmjopen.bmj.com/site/about/guidelines.xhtml

26. Stroux, L., et al., *An mHealth monitoring system for traditional birth attendant-led antenatal risk assessment in rural Guatemala.* Journal of medical engineering & technology, 2016. **40**(7-8): p. 356-371.
27. Martinez, B., et al., *Agile Development of a Smartphone App for Perinatal Monitoring in a Resource-Constrained Setting.* J Health Inform Dev Ctries, 2017. **11**(1).
28. Martinez, B., et al., *mHealth intervention to improve the continuum of maternal and perinatal care in rural Guatemala: a pragmatic, randomized controlled feasibility trial.* Reprod Health, 2018. **15**(1): p. 120.
29. Zaidi, S., et al., *Operability, Usefulness, and Task-Technology Fit of an mHealth App for Delivering Primary Health Care Services by Community Health Workers in Underserved Areas of Pakistan and Afghanistan: Qualitative Study.* J Med Internet Res, 2020. **22**(9): p. e18414.
30. Indriani, D., et al., *The maternal referral mobile application system for minimizing the risk of childbirth.* J Public Health Res, 2020. **9**(2): p. 1813.
31. Velez, O., et al., *A usability study of a mobile health application for rural Ghanaian midwives.* J Midwifery Womens Health, 2014. **59**(2): p. 184-91.
32. Tomlinson, M.R.-B., MJ; Swartz, L; Tsai, AC; , *Scaling Up mHealth: Where Is the Evidence?* . PLoS Medicine 2013. **10(2).**
33. Agarwal, S.P., HB; Long, LA; Labrique, AB *Evidence on feasibility and effective use of mHealth strategies by frontline health workers in developing countries: systematic review.* Tropical Medicine and International Health, 2015. **20**(8): p. 1003-1014.
34. Balikuddembe, M.S., et al., *Computerized Childbirth Monitoring Tools for Health Care Providers Managing Labor: A Scoping Review.* JMIR Med Inform, 2017. **5**(2): p. e14.


a



For peer review only - http://bmjopen.bmj.com/site/about/guidelines.xhtml

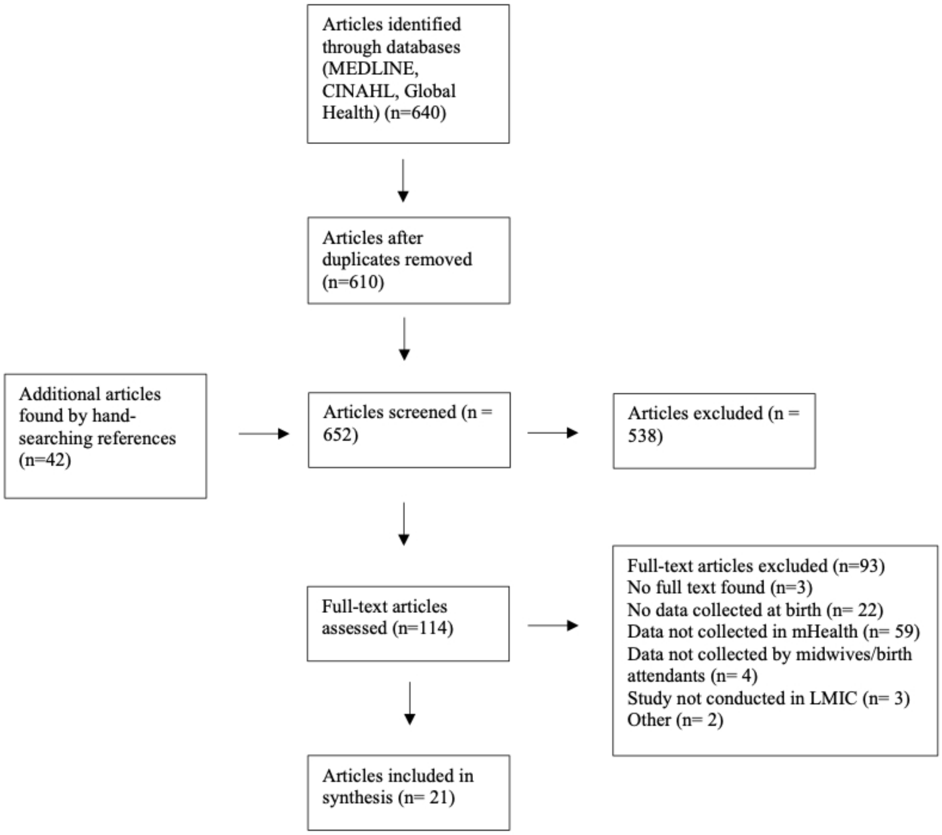


Figure 1: MEDLINE, CINAHL and Global Health were searched for relevant references. These were first screened by citation then full-text. Articles selected for inclusion were then hand-searched for relevant references.

295x248mm (72 x 72 DPI)

***APPENDIX***

Search strategies:

PubMed:

((parturition* OR childbirth* OR labour*) OR ("Parturition"[Mesh])) AND (((((("Electronic Health Records"[Mesh]) OR ( "Mobile Applications"[Mesh] OR "Cell Phone"[Mesh] )) OR ( "Cell Phone Use"[Mesh] OR "Computers, Handheld"[Mesh] )) OR (mobile phone* or mobile* or mhealth* or electronic* or smartphone* or iPhone* or iPad* or tablet computer* or cellphone* or m-health* or ehealth* or e-health* or cell phone* or handheld computer*)) AND ((Africa or Caribbean or "West Indies" or "South America" or "Latin America" or "Central America" or Afghanistan or Albania or Algeria or Angola or "American Samoa" or Armenia or Armenian or Azerbaijan or Bangladesh or Benin or Byelarus or Byelorussian or Belarus or Belorussian or Belorussia or Belize or Bhutan or Bolivia or Bosnia or Herzegovina or Hercegovina or Botswana or Brazil or Brasil or Bulgaria or "Burkina Faso" or "Burkina Fasso" or "Upper Volta" or Burundi or Urundi or Cambodia or "Khmer Republic" or Kampuchea or Cameroon or Cameroons or Cameron or Camerons or "Cape Verde" or "Central African Republic" or Chad or China or Colombia or Comoros or "Comoro Islands" or Comores or Mayotte or Congo or Zaire or "Costa Rica" or "Cote d'Ivoire" or "Ivory Coast" or Cuba or Djibouti or "French Somaliland" or Dominica or "Dominican Republic" or "East Timor" or "East Timur" or "Timor Leste" or Ecuador or Egypt or "United Arab Republic" or "El Salvador" or Eritrea or Ethiopia or Fiji or Gabon or "Gabonese Republic" or Gambia or Gaza or "Georgia Republic" or "Georgian Republic" or Ghana or "Gold Coast" or Grenada or Guatemala or Guinea or Guinea-Bissau or Guam or Guiana or Guyana or Haiti or Honduras or India or Maldives or Indonesia or Iran or Iraq or Jamaica or Jordan or Kazakhstan or Kazakh or Kenya or Kiribati or Korea or Kosovo or Kyrgyzstan or Kirghizia or "Kyrgyz Republic" or Kirghiz or Kirgizstan or "Lao PDR" or Laos or Lebanon or Lesotho or Basutoland or Liberia or Libya or Macedonia or Madagascar or "Malagasy Republic" or Malaysia or Malaya or Malay or Sabah or Sarawak or Malawi or Nyasaland or Mali or "Marshall Islands" or Mauritania or Mauritius or "Agalega Islands" or Mexico or Micronesia or "Middle East" or Moldova or Moldovia or Moldovian or Mongolia or Montenegro or Morocco or Ifni or Mozambique or Myanmar or Myanma or Burma or Namibia or Nepal or "Netherlands Antilles" or Nicaragua or Niger or Nigeria or Pakistan or Palau or Palestine or Panama or "Papua New Guinea" or Paraguay or Peru or Philippines or Philipines or Phillipines or Phillippines or Romania or Rumania or Roumania or Rwanda or Ruanda or "Saint Lucia" or "St Lucia" or "Saint Vincent" or "St Vincent" or Grenadines or Samoa or "Samoan Islands" or "Navigator Island" or "Navigator Islands" or "Sao Tome" or Senegal or Serbia or "Sierra Leone" or Sri Lanka or Ceylon or "Solomon Islands" or Somalia or Sudan or Suriname or Surinam or Swaziland or Syria or Principe or Tajikistan or Tadzhikistan or Tadjikistan or Tadzhik or Tanzania or Thailand or Timor-Leste or Togo or "Togolese Republic" or Tonga or Tunisia or Turkey or Turkmenistan or Turkmen or Tuvalu or Uganda or Ukraine or Uzbekistan or Uzbek or Vanuatu or "New Hebrides" or Vietnam or "Viet Nam" or "West Bank" or Yemen or Zambia or Zimbabwe or Rhodesia or developing countr* or less developed countr* or under developed countr* or underdeveloped countr* or middle income countr* or low income countr* or deprived countr* or underserved countr* or under served countr* or poor countr* or developing nation* or less developed nation* or under developed nation* or underdeveloped nation* or middle income

For peer review only

nation* or low income nation* or deprived nation* or underserved nation* or under served nation* or poor nation* or LMIC or lmics or third world* or lami countr* or transitional countr* or "global health") OR ("Developing Countries"[Mesh]))) AND (("Midwifery"[Mesh]) OR (midwife* or midwives or birth attendant* or doula* or childbirth assistant*)))

Global Health:

((mobile phone* or mobile* or mhealth* or electronic* or smartphone* or iPhone* or iPad* or tablet computer* or cellphone* or m-health* or ehealth* or e-health* or cell phone* or handheld computer).mp. [mp=abstract, title, original title, broad terms, heading words, identifiers, cabicodes] OR (mobile telephones/ OR mobile equipment/)) AND ((midwife* or midwives or birth attendant* or doula* or childbirth assistant*).mp. [mp=abstract, title, original title, broad terms, heading words, identifiers, cabicodes] OR ((traditional birth attendants or midwives).sh)) AND ((childbirth/) OR ((childbirth* or labour*).mp. [mp=abstract, title, original title, broad terms, heading words, identifiers, cabicodes]) AND ((Developing Countries/) OR (Africa or Caribbean or "West Indies" or "South America" or "Latin America" or "Central America" or Afghanistan or Albania or Algeria or Angola or "American Samoa" or Armenia or Armenian or Azerbaijan or Bangladesh or Benin or Byelarus or Byelorussian or Belarus or Belorussian or Belorussia or Belize or Bhutan or Bolivia or Bosnia or Herzegovina or Hercegovina or Botswana or Brazil or Brasil or Bulgaria or "Burkina Faso" or "Burkina Fasso" or "Upper Volta" or Burundi or Urundi or Cambodia or "Khmer Republic" or Kampuchea or Cameroon or Cameroons or Cameron or Camerons or "Cape Verde" or "Central African Republic" or Chad or China or Colombia or Comoros or "Comoro Islands" or Comores or Mayotte or Congo or Zaire or "Costa Rica" or "Cote d'Ivoire" or "Ivory Coast" or Cuba or Djibouti or "French Somaliland" or Dominica or "Dominican Republic" or "East Timor" or "East Timur" or "Timor Leste" or Ecuador or Egypt or "United Arab Republic" or "El Salvador" or Eritrea or Ethiopia or Fiji or Gabon or "Gabonese Republic" or Gambia or Gaza or "Georgia Republic" or "Georgian Republic" or Ghana or "Gold Coast" or Grenada or Guatemala or Guinea or Guinea-Bissau or Guam or Guiana or Guyana or Haiti or Honduras or India or Maldives or Indonesia or Iran or Iraq or Jamaica or Jordan or Kazakhstan or Kazakh or Kenya or Kiribati or Korea or Kosovo or Kyrgyzstan or Kirghizia or "Kyrgyz Republic" or Kirghiz or Kirgizstan or "Lao PDR" or Laos or Lebanon or Lesotho or Basutoland or Liberia or Libya or Macedonia or Madagascar or "Malagasy Republic" or Malaysia or Malaya or Malay or Sabah or Sarawak or Malawi or Nyasaland or Mali or "Marshall Islands" or Mauritania or Mauritius or "Agalega Islands" or Mexico or Micronesia or "Middle East" or Moldova or Moldovia or Moldovian or Mongolia or Montenegro or Morocco or Ifni or Mozambique or Myanmar or Myanma or Burma or Namibia or Nepal or "Netherlands Antilles" or Nicaragua or Niger or Nigeria or Pakistan or Palau or Palestine or Panama or "Papua New Guinea" or Paraguay or Peru or Philippines or Philipines or Phillipines or Phillippines or Romania or Rumania or Roumania or Rwanda or Ruanda or "Saint Lucia" or "St Lucia" or "Saint Vincent" or "St Vincent" or Grenadines or Samoa or "Samoan Islands" or "Navigator Island" or "Navigator Islands" or "Sao Tome" or Senegal or Serbia or "Sierra Leone" or Sri Lanka or Ceylon or "Solomon Islands" or Somalia or Sudan or Suriname or Surinam or Swaziland or Syria or Principe or Tajikistan or Tadzhikistan or Tadjikistan or Tadzhik or Tanzania or Thailand or Timor-Leste or Togo or "Togolese Republic" or Tonga or Tunisia or Turkey or Turkmenistan or Turkmen or Tuvalu or Uganda or Ukraine or Uzbekistan or Uzbek or Vanuatu or "New Hebrides" or Vietnam or "Viet Nam" or "West Bank" or Yemen or Zambia or

For peer review only

Zimbabwe or Rhodesia or developing countr* or less developed countr* or under developed countr* or underdeveloped countr* or middle income countr* or low income countr* or deprived countr* or underserved countr* or under served countr* or poor countr* or developing nation* or less developed nation* or under developed nation* or underdeveloped nation* or middle income nation* or low income nation* or deprived nation* or underserved nation* or under served nation* or poor nation* or LMIC or lmics or third world* or lami countr* or transitional countr* or "global health").mp. [mp=abstract, title, original title, broad terms, heading words, identifiers, cabicodes])

CINAHL:

( mh:("Midwifery") OR (midwife* or midwives or birth attendant* or doula* or childbirth assistant*)) AND (mh:("Labour") OR (childbirth* OR labour*)) AND (mh:("Cellular Phone" OR "Mobile Applications" OR "Electronic Health Records" OR "Computers, Hand-Held" OR "Telemedicine") OR (mobile phone* or mobile* or mhealth* or electronic* or smartphone* or iPhone* or iPad* or tablet computer* or cellphone* or m-health* or ehealth* or e-health* or cell phone* or handheld computer*)) AND (mh:("Developing Countries") OR (Africa or Caribbean or "West Indies" or "South America" or "Latin America" or "Central America" or Afghanistan or Albania or Algeria or Angola or "American Samoa" or Armenia or Armenian or Azerbaijan or Bangladesh or Benin or Byelarus or Byelorussian or Belarus or Belorussian or Belorussia or Belize or Bhutan or Bolivia or Bosnia or Herzegovina or Hercegovina or Botswana or Brazil or Brasil or Bulgaria or "Burkina Faso" or "Burkina Fasso" or "Upper Volta" or Burundi or Urundi or Cambodia or "Khmer Republic" or Kampuchea or Cameroon or Cameroons or Cameron or Camerons or "Cape Verde" or "Central African Republic" or Chad or China or Colombia or Comoros or "Comoro Islands" or Comores or Mayotte or Congo or Zaire or "Costa Rica" or "Cote d'Ivoire" or "Ivory Coast" or Cuba or Djibouti or "French Somaliland" or Dominica or "Dominican Republic" or "East Timor" or "East Timur" or "Timor Leste" or Ecuador or Egypt or "United Arab Republic" or "El Salvador" or Eritrea or Ethiopia or Fiji or Gabon or "Gabonese Republic" or Gambia or Gaza or "Georgia Republic" or "Georgian Republic" or Ghana or "Gold Coast" or Grenada or Guatemala or Guinea or Guinea-Bissau or Guam or Guiana or Guyana or Haiti or Honduras or India or Maldives or Indonesia or Iran or Iraq or Jamaica or Jordan or Kazakhstan or Kazakh or Kenya or Kiribati or Korea or Kosovo or Kyrgyzstan or Kirghizia or "Kyrgyz Republic" or Kirghiz or Kirgizstan or "Lao PDR" or Laos or Lebanon or Lesotho or Basutoland or Liberia or Libya or Macedonia or Madagascar or "Malagasy Republic" or Malaysia or Malaya or Malay or Sabah or Sarawak or Malawi or Nyasaland or Mali or "Marshall Islands" or Mauritania or Mauritius or "Agalega Islands" or Mexico or Micronesia or "Middle East" or Moldova or Moldovia or Moldovian or Mongolia or Montenegro or Morocco or Ifni or Mozambique or Myanmar or Myanma or Burma or Namibia or Nepal or "Netherlands Antilles" or Nicaragua or Niger or Nigeria or Pakistan or Palau or Palestine or Panama or "Papua New Guinea" or Paraguay or Peru or Philippines or Philipines or Phillipines or Phillippines or Romania or Rumania or Roumania or Rwanda or Ruanda or "Saint Lucia" or "St Lucia" or "Saint Vincent" or "St Vincent" or Grenadines or Samoa or "Samoan Islands" or "Navigator Island" or "Navigator Islands" or "Sao Tome" or Senegal or Serbia or "Sierra Leone" or Sri Lanka or Ceylon or "Solomon Islands" or Somalia or Sudan or Suriname or Surinam or Swaziland or Syria or Principe or Tajikistan or Tadzhikistan or Tadjikistan or Tadzhik or Tanzania or Thailand or Timor-Leste or Togo or "Togolese Republic" or Tonga or Tunisia or Turkey or Turkmenistan or Turkmen or Tuvalu or Uganda or Ukraine or Uzbekistan or Uzbek or Vanuatu or "New Hebrides" or Vietnam or "Viet Nam" or "West Bank" or

For peer review only

Yemen or Zambia or Zimbabwe or Rhodesia or developing countr* or less developed countr* or under developed countr* or underdeveloped countr* or middle income countr* or low income countr* or deprived countr* or underserved countr* or under served countr* or poor countr* or developing nation* or less developed nation* or under developed nation* or underdeveloped nation* or middle income nation* or low income nation* or deprived nation* or underserved nation* or under served nation* or poor nation* or LMIC or lmics or third world* or lami countr* or transitional countr* or "global health"))

For peer review only

**Preferred Reporting Items for Systematic reviews and Meta-Analyses extension for Scoping Reviews (PRISMA-ScR) Checklist**

| SECTION | ITEM | PRISMA-ScR CHECKLIST ITEM | REPORTED ON PAGE # |
|---|---|---|---|
| **TITLE** | | | |
| Title | 1 | Identify the report as a scoping review. | 3 |
| **ABSTRACT** | | | |
| Structured summary | 2 | Provide a structured summary that includes (as applicable): background, objectives, eligibility criteria, sources of evidence, charting methods, results, and conclusions that relate to the review questions and objectives. | 1 |
| **INTRODUCTION** | | | |
| Rationale | 3 | Describe the rationale for the review in the context of what is already known. Explain why the review questions/objectives lend themselves to a scoping review approach. | 3 |
| Objectives | 4 | Provide an explicit statement of the questions and objectives being addressed with reference to their key elements (e.g., population or participants, concepts, and context) or other relevant key elements used to conceptualize the review questions and/or objectives. | 4 |
| **METHODS** | | | |
| Protocol and registration | 5 | Indicate whether a review protocol exists; state if and where it can be accessed (e.g., a Web address); and if available, provide registration information, including the registration number. | 4 |
| Eligibility criteria | 6 | Specify characteristics of the sources of evidence used as eligibility criteria (e.g., years considered, language, and publication status), and provide a rationale. | 4-5 |
| Information sources* | 7 | Describe all information sources in the search (e.g., databases with dates of coverage and contact with authors to identify additional sources), as well as the date the most recent search was executed. | 4-5 |
| Search | 8 | Present the full electronic search strategy for at least 1 database, including any limits used, such that it could be repeated. | 25-28 |
| Selection of sources of evidence† | 9 | State the process for selecting sources of evidence (i.e., screening and eligibility) included in the scoping review. | 4-6 |
| Data charting process‡ | 10 | Describe the methods of charting data from the included sources of evidence (e.g., calibrated forms or forms that have been tested by the team before their use, and whether data charting was done independently or in duplicate) and any processes for obtaining and confirming data from investigators. | 4-6 |
| Data items | 11 | List and define all variables for which data were sought and any assumptions and simplifications made. | 5 |
| Critical appraisal of individual sources of evidence§ | 12 | If done, provide a rationale for conducting a critical appraisal of included sources of evidence; describe the methods used and how this information was used in any data synthesis (if appropriate). | NA |

| SECTION | ITEM | PRISMA-ScR CHECKLIST ITEM | REPORTED ON PAGE # |
|---|---|---|---|
| Synthesis of results | 13 | Describe the methods of handling and summarizing the data that were charted. | 5 |
| **RESULTS** | | | |
| Selection of sources of evidence | 14 | Give numbers of sources of evidence screened, assessed for eligibility, and included in the review, with reasons for exclusions at each stage, ideally using a flow diagram. | 5-6 |
| Characteristics of sources of evidence | 15 | For each source of evidence, present characteristics for which data were charted and provide the citations. | 20-24 |
| Critical appraisal within sources of evidence | 16 | If done, present data on critical appraisal of included sources of evidence (see item 12). | NA |
| Results of individual sources of evidence | 17 | For each included source of evidence, present the relevant data that were charted that relate to the review questions and objectives. | 7-12, 17-24 |
| Synthesis of results | 18 | Summarize and/or present the charting results as they relate to the review questions and objectives. | 7-12 |
| **DISCUSSION** | | | |
| Summary of evidence | 19 | Summarize the main results (including an overview of concepts, themes, and types of evidence available), link to the review questions and objectives, and consider the relevance to key groups. | 12-13 |
| Limitations | 20 | Discuss the limitations of the scoping review process. | 13 |
| Conclusions | 21 | Provide a general interpretation of the results with respect to the review questions and objectives, as well as potential implications and/or next steps. | 13 |
| **FUNDING** | | | |
| Funding | 22 | Describe sources of funding for the included sources of evidence, as well as sources of funding for the scoping review. Describe the role of the funders of the scoping review. | 13 |

JBI = Joanna Briggs Institute; PRISMA-ScR = Preferred Reporting Items for Systematic reviews and Meta-Analyses extension for Scoping Reviews.

* Where *sources of evidence* (see second footnote) are compiled from, such as bibliographic databases, social media platforms, and Web sites.

† A more inclusive/heterogeneous term used to account for the different types of evidence or data sources (e.g., quantitative and/or qualitative research, expert opinion, and policy documents) that may be eligible in a scoping review as opposed to only studies. This is not to be confused with *information sources* (see first footnote).

‡ The frameworks by Arksey and O'Malley (6) and Levac and colleagues (7) and the JBI guidance (4, 5) refer to the process of data extraction in a scoping review as data charting.

§ The process of systematically examining research evidence to assess its validity, results, and relevance before using it to inform a decision. This term is used for items 12 and 19 instead of "risk of bias" (which is more applicable to systematic reviews of interventions) to include and acknowledge the various sources of evidence that may be used in a scoping review (e.g., quantitative and/or qualitative research, expert opinion, and policy document).

*From:* Tricco AC, Lillie E, Zarin W, O'Brien KK, Colquhoun H, Levac D, et al. PRISMA Extension for Scoping Reviews (PRISMAScR): Checklist and Explanation. Ann Intern Med. 2018;169:467–473. doi: 10.7326/M18-0850.



For peer review only - http://bmjopen.bmj.com/site/about/guidelines.xhtml

BMJ Open

# The use of mHealth tools to register birth outcomes in low- and middle-income countries: a scoping review

| | |
|---|---|
| Journal: | *BMJ Open* |
| Manuscript ID | bmjopen-2022-063886.R1 |
| Article Type: | Original research |
| Date Submitted by the Author: | 11-Aug-2022 |
| Complete List of Authors: | Cansdale, Lottie; University of Oxford Medical Sciences Division,<br>Kelly, Gabriella; University of Oxford Medical Sciences Division<br>Khashan, Ali; University College Cork School of Public Health; INFANT Research Centre, University College Cork, Ireland<br>Malata, Address; Malawi University of Science and Technology<br>Kachale, Fannie; Malawi Ministry of Health<br>Lissauer, David ; Malawi-Liverpool-Wellcome Trust Clinical Research Programme; University of Liverpool, Institute of Life Course and Medical Sciences<br>Yosefe, Simeon; Malawi Ministry of Health, Central Monitoring and Evaluation Division<br>Roberts, James; Magee-Women's Research Institute<br>Woodworth, Simon; University College Cork Business School; INFANT Research Centre, University College Cork, Ireland<br>Mmbaga, Blandina; Kilimanjaro Christian Medical University College, Kilimanjaro Clinical Research Institute<br>Redman, Christopher ; University of Oxford, Nuffield Department of Women’s & Reproductive Health<br>Hirst, Jane Elizabeth; University of Oxford, Nuffield Department of Women's and Reproductive Health |
| <b>Primary Subject Heading</b>: | Global health |
| Secondary Subject Heading: | Obstetrics and gynaecology, Public health |
| Keywords: | OBSTETRICS, Information technology < BIOTECHNOLOGY & BIOINFORMATICS, Health informatics < BIOTECHNOLOGY & BIOINFORMATICS |
| | |

SCHOLARONE™
Manuscripts

*I, the Submitting Author has the right to grant and does grant on behalf of all authors of the Work (as defined in the below author licence), an exclusive licence and/or a non-exclusive licence for contributions from authors who are: i) UK Crown employees; ii) where BMJ has agreed a CC-BY licence shall apply, and/or iii) in accordance with the terms applicable for US Federal Government officers or employees acting as part of their official duties; on a worldwide, perpetual, irrevocable, royalty-free basis to BMJ Publishing Group Ltd ("BMJ") its licensees and where the relevant Journal is co-owned by BMJ to the co-owners of the Journal, to publish the Work in this journal and any other BMJ products and to exploit all rights, as set out in our licence.*

*The Submitting Author accepts and understands that any supply made under these terms is made by BMJ to the Submitting Author unless you are acting as an employee on behalf of your employer or a postgraduate student of an affiliated institution which is paying any applicable article publishing charge ("APC") for Open Access articles. Where the Submitting Author wishes to make the Work available on an Open Access basis (and intends to pay the relevant APC), the terms of reuse of such Open Access shall be governed by a Creative Commons licence – details of these licences and which Creative Commons licence will apply to this Work are set out in our licence referred to above.*

*Other than as permitted in any relevant BMJ Author's Self Archiving Policies, I confirm this Work has not been accepted for publication elsewhere, is not being considered for publication elsewhere and does not duplicate material already published. I confirm all authors consent to publication of this Work and authorise the granting of this licence.*

For peer review only

# The use of mHealth tools to register birth outcomes in low- and middle-income countries: a scoping review

LG Cansdale[1], G Kelly[1], AS Khashan[2,3], A Malata[4], F Kachale[5], D Lissauer[6,7], S Yosefe[5], JM Roberts[8], S Woodworth[3,9], BT Mmbaga[10], C Redman[11], JE Hirst[11]

1. University of Oxford Medical School, UK
2. School of Public Health, University College Cork, Ireland
3. INFANT Research Centre, University College Cork, Ireland
4. Malawi University of Science and Technology, Malawi
5. Ministry of Health, Malawi
6. Malawi-Liverpool Wellcome Research Programme, Blantyre, Malawi
7. Institute of Life Course and Medical Sciences, University of Liverpool, UK
8. Magee Women's Research Institute, Department of Obstetrics and Gynecology, Epidemiology and Clinical and Translational Research, University of Pittsburgh, USA
9. Cork University Business School, University College Cork, Ireland
10. Kilimanjaro Clinical Research Institute, Kilimanjaro Christian Medical Centre and Kilimanjaro Christian Medical University College, Moshi, Tanzania
11. Nuffield Department of Women's & Reproductive Health, University of Oxford, UK

Corresponding author: LG Cansdale, University of Oxford Medical School, UK. Email: lottie.cansdale@st-hildas.ox.ac.uk.

For peer review only

**Objective:** Accurate reporting of birth outcomes in low- and middle-income countries (LMICs) is essential. It has been proposed that conventional paper-based registers could be replaced by mobile (mHealth) tools. mHealth could provide timely data for individual facilities and health departments, as well as capture deliveries occurring outside facilities. This scoping review evaluates which mHealth tools have been reported to birth outcomes in the delivering room in LMICs and documents their reported advantages and drawbacks.

**Design:** A scoping review following PRISMA and JBI guidelines for scoping reviews and the mERA checklist for evaluating mHealth interventions.

**Data Sources:** PubMed, CINAHL and Global Health were searched through 03/02/2022.

**Eligibility criteria:** Studies were included where mHealth tools were used by health workers in LMICs to record birth outcomes. Exclusion criteria included mHealth not being used at the point of delivery, non-peer reviewed literature and studies not written in English.

**Data Extraction and synthesis:** Two independent reviewers screened studies and extracted data. Common themes among studies were identified.

**Results**: 640 records were screened, 21 of which met the inclusion criteria, describing 15 different mHealth tools. We identified six themes: (1) digital tools for labour monitoring (8 studies); (2) digital data collection of specific birth outcomes (3 studies); (3) digital technologies used in community settings (6 studies); (4) attitudes of health workers (10 studies); (5) paper vs electronic data collection (3 studies); and (6) infrastructure, interoperability, and sustainability (8 studies).

**Conclusion**: Several mHealth technologies are reported to have the capability to record birth outcomes at the point of delivery, but none were identified that were designed only for that purpose. Use of digital delivery registers appears feasible and acceptable to health workers, but definitive evaluations are lacking. More research is needed to determine the sustainability of technologies and ability to integrate with existing health information systems.

**Strengths and limitations of this study:**

1. We have used a comprehensive search strategy to explore the literature regarding mHealth and digital birth registers in LMICs.

2. We have reported data items using the mERA checklist, which was developed by the WHO to improve reporting of mHealth technologies.

For peer review only

3. Only peer-reviewed articles have been included, meaning relevant data in the grey literature or technologies not reported in peer-reviewed articles may have been omitted.

For peer review only

# *The use of mHealth tools to register birth outcomes in low- and middle-income countries: a scoping review*

## *Introduction*

Documentation of birth and perinatal events in low- and middle-income countries (LMICs) and obtaining accurate statistics relating to mortality and morbidity can be challenging. Accurate data are needed to monitor health outcomes, to identify problems and to track the impact of interventions, as well as to understand the effects of health system shocks such as COVID-19. Whilst electronic medical records can enable rapid monitoring of trends within units, regions and countries, such hospital-based electronic record systems are expensive, requiring large investments in computer hardware, networks and ongoing technical and security maintenance and support, all of which can be barriers for implementation in LMICs.

Traditionally, births in LMICs are recorded in paper-based delivery registers. These are usually large books, completed by birth attendants soon after the birth. Whilst their content and quality can be variable, they are often the only source of data for collation of perinatal health statistics. Paper-based registers have a short memory and are difficult to store due to their large size. The information in delivery registers is difficult to access when it is needed for patient care at a later date, for comparing data between institutions, or for monitoring trends in care and outcomes over time. Furthermore, non-institutional births may not be recorded at all. LMICs have a high proportion of non-facility births; for example, only 48% of births in Ethiopia, 53% in Bangladesh and 65% in Guatemala take place in healthcare facilities [1]. LMICs are also the countries with the highest maternal and neonatal mortality rates; maternal deaths per 100,000 are estimated at 375 in Uganda, 342 in Kenya and 177 in Indonesia [2]. In other words, those most at risk and in need are those least likely to be documented.

The use of mobile phones is increasing rapidly. One report suggested ownership rates of 80% in 'emerging economies', of which 47% are smartphones [3]. Given the increasing ubiquity of smartphones, their use could



For peer review only - http://bmjopen.bmj.com/site/about/guidelines.xhtml

improve reporting of delivery and birth outcomes. This application is termed mHealth, and is defined by the WHO as 'the use of mobile wireless technologies for health' [4]. mHealth is already being used in many low-income settings to register pregnancy and to send reminders of antenatal appointments, with one systematic review identifying 19 different studies describing 15 mHealth technologies used to document maternal health interventions in LMICs [5]. However, it is unclear at present to what extent mHealth is used to capture birth data at the place of delivery, and whether this approach improves the recording of perinatal outcomes. Hence the purpose of this scoping review is to understand what mobile data collection technologies have been evaluated to record births outcomes at the time and place of delivery in LMICs.

For peer review only

## *Methods*

This review was developed in line with the PRISMA and JBI (Joanna Briggs Institute) guidelines for scoping reviews [6, 7], using the PCC mnemonic – population, concept, context. A scoping review gives an overview of the evidence, maps the literature and identifies and analyses current gaps in knowledge. A review protocol was not published for this study.

### *Population, Concept, Context*

*Population*

The population of this review comprises healthcare workers recording delivery outcomes, namely midwives, community health workers, doctors and other cadres of skilled birth attendants in LMICs. LMICs were defined according to the World Bank categorisation of low, lower-middle and upper -middle in 2021 [8].

*Concept*

Our focus is the use of mHealth to capture data at birth, and specifically those technologies present at the place of delivery. Of these many are expected to have wider applications, such as recording of the outcomes of antenatal



For peer review only - http://bmjopen.bmj.com/site/about/guidelines.xhtml

and postnatal appointments, or progress in labour via an e-partogram. For this scoping review, we have included any technology if outcomes at birth were recorded by the birth attendant or community health worker, at the time of, or shortly after, delivery.

*Context*

The context of the review are the places where births occur in LMICs. This includes home and community deliveries, as well as institutional births in health centres, nursing homes and government or private hospitals.

***Search Strategy***

To identify relevant sources, three databases were searched: PubMed, CINAHL and GLOBAL HEALTH. The search strategy was created with an experienced librarian and further refined by the authors, including key search terms such as ‘childbirth’, ‘mobile applications’ and ‘midwives’. The final search strategies for each database can be found in the appendix. The databases were searched for relevant articles up to March 2022, with a lower date limit not specified as the term ‘mHealth’ was not widely used prior to 2000. The final searches were uploaded into EndNote and duplicates were removed by their duplicate detection software.

***Screening and selection of relevant studies.***

Once the citations had been extracted to EndNote, they were uploaded on to Colandr (colandrapp.com), a software that manages citation and full-text screening for reviews. Texts were first screened by abstract, and those that met the inclusion criteria then went on to the full-text screen. Two reviewers (LC and GK) did both citation and full-text screening of articles, and any disputes between reviewers were resolved by a third author (JH).

References of studies included for full-text screening were hand-searched to idenitfy for further relevant studies. These hand-picked citations were uploaded to EndNote and then included in citation screening. Once imported into EndNote duplicates were removed.

***Inclusion and exclusion Criteria***

For peer review only



For peer review only - http://bmjopen.bmj.com/site/about/guidelines.xhtml

Eligible studies were conducted in LMICs (defined by the World Bank list of LMICs) [8]. Within each study, the mHealth tool needed to be capable of collecting data regarding birth outcomes at the place of delivery. We considered studies where the data was collected by nurses/midwives/birth attendants. We limited the search to English language publications only.

We excluded studies where no birth outcomes were reported in the publication, where data were collected using hospital Electronic Health Record (EHR) systems on desktop computers, where birth registration data were collected after the delivery or for the purpose of vital registration only, and where data were collected by people other than nurses/midwives/birth attendants. As this was intended as a scoping review of published literature, we excluded non-peer-reviewed reports of technologies, such as reports, web pages and media releases.

*Data extraction and synthesis*

An Excel spreadsheet was used for data-extraction, with variables to extract agreed through discussion between the authors. The two reviewers (LC and GK) independently charted data and discussed results.

We abstracted data on article characteristics, including country of origin, funder, form of mHealth, technology user, reported birth outcomes and technology feasibility and acceptability.

We conducted a narrative synthesis based on our findings. We first mapped studies by geographic location, technology type, primary intended use of technology, accessibility outside the program described (i.e., whether the technology was Open Access or not), and intended user group (hospital/clinic staff or in the community). We then identified the main thematic areas that have been evaluated to date, with the purpose of summarising the current state of the academic literature in this field and identifying gaps for further research. We made particular use of mERA (mHealth Evidence Reporting and Assessment), a checklist which was developed by the WHO to standardise mHealth evidence reporting [9]. Given this was a scoping review to map the quantity and domains of evidence on digital birth registers, the quality of studies was not assessed.

*Patient and public involvement*


For peer review only - http://bmjopen.bmj.com/site/about/guidelines.xhtml

Neither patients nor members of the public were involved in any aspect of this review.

## *Results*

### 1. Identification of eligible studies for review

The search strategy is outlined in Figure 1. The initial search was carried out on 31/07/2021 and updated on 03/02/2022, yielding 567 and 640 records respectively. Of the records identified, 578 were from PubMed, 34 from CINAHL, 28 from Global Health.

For peer review only

## 2. Study Characteristics and Themes

The characteristics of each study are described in Table 1, together with the six specific themes we identified. The themes are elaborated in more detail in sections 3-6 below. Table 2 describes the technical aspects and content of interventions.

For peer review only



For peer review only - http://bmjopen.bmj.com/site/about/guidelines.xhtml

Table 1: study characteristics and themes

| Technology<br>Author/s, year/s | Country | Study design[1],<br>N participants | Labour[a] | Outcomes[b] | Comm[c] | Attitudes[d] | Paper vs elect[e] | Sustain[f] |
|---|---|---|---|---|---|---|---|---|
| **1. ePartogram** | | | | | | | | |
| Litwin et al, 2018 [10] | Tanzania | Feasibility and acceptability<br>23 skilled birth attendants | ● (green) | ● (red) | ● (red) | ● (green) | ● (yellow) | ● (yellow) |
| Sanghvi et al, 2019 [11] | Kenya | Mixed method, quasi-experimental<br>110 skilled birth attendants | ● (green) | ● (red) | ● (red) | ● (green) | ● (green) | ● (red) |
| **2. E-partograph** | | | | | | | | |
| Rahman et al, 2019 [12] | Bangladesh | Prospective cross-over study<br>Nurse-midwives (NNS*) | ● (green) | ● (red) | ● (red) | ● (green) | ● (yellow) | ● (yellow) |
| Tadesse et al, 2019 [13] | Ethiopia | Cross-sectional study with questionnaire<br>462 obstetric care providers | ● (green) | ● (red) | ● (red) | ● (green) | ● (yellow) | ● (yellow) |
| **3. mLabour** | | | | | | | | |
| Schweers et al, 2016 [14] | India | Design and preliminary evaluation | ● (green) | ● (red) | ● (red) | ● (green) | ● (yellow) | ● (red) |



For peer review only - http://bmjopen.bmj.com/site/about/guidelines.xhtml

For peer review only

| | | | | | | | | |
|---|---|---|---|---|---|---|---|---|
| | | 12 nurses | | | | | | |
| **4. prasavGraph** | | | | | | | | |
| Singh et al, 2016 [15] | India | Development and pilot<br>Doctors and student nurses (NNS) | green | red | red | red | red | yellow |
| **5. ASMAN** | | | | | | | | |
| Usmanova et al 2020 [16] | India | Qualitative study<br>44 stakeholders | green | red | yellow | green | yellow | green |
| Usmanova et al 2021 [17] | India | Secondary analysis of programme data<br>(NNS) | green | red | red | red | red | red |
| **6. PPH reporting** | | | | | | | | |
| Andreatta et al, 2011[18] | Ghana | Evaluation of technology<br>10 birth attendants | red | green | green | red | red | red |
| **7. Mobile Delivery Timer** | | | | | | | | |
| Somannavar et al 2015 [19] | India | Development and evaluation<br>46 birth attendants | red | green | red | green | red | yellow |
| **8. Birth defects surveillance system** | | | | | | | | |
| Mumpe-Mwanja et al, 2019 [20] | Uganda | Surveillance study<br>Midwives (NNS) | red | green | red | red | red | red |
| **9. RapidSMS-MCH** | | | | | | | | |
| Ngabo et al, 2012 [21] | Rwanda | Design and implementation<br>432 community health workers | red | red | green | red | red | green |
| Musabyimana et al, 2018 [22] | Rwanda | Qualitative study<br>93 participants including community health workers | red | red | green | green | red | green |
| **10. Data collection** | | | | | | | | |
| Little et al, 2013 [23] | Ethiopia | Development and evaluation<br>12 midwives, 20 health extension workers | red | red | green | green | red | green |
| Medhanyie et al, 2015 [24] | Ethiopia | Semi-structured questionnaire<br>23 midwives and health extension workers | red | red | green | green | green | green |

For peer review only

| | | | | | | | | |
|---|---|---|---|---|---|---|---|---|
| Medhanyie et al, 2017[25] | Ethiopia | Comparative cross-sectional study<br>25 midwives and health extension workers | ● (red) | ● (red) | ● (green) | ● (red) | ● (green) | ● (red) |
| **11. Perinatal Monitoring App** | | | | | | | | |
| Stroux et al 2016 [26] | Guatemala | Prototype testing<br>9 healthcare workers | ● (red) | ● (red) | ● (green) | ● (yellow) | ● (red) | ● (green) |
| Martinez et al, 2017[27] | Guatemala | Agile development<br>25 midwives | ● (red) | ● (red) | ● (green) | ● (yellow) | ● (red) | ● (green) |
| Martinez et al, 2018 [28] | Guatemala | Randomised control trial<br>44 traditional birth attendants | ● (red) | ● (red) | ● (green) | ● (red) | ● (red) | ● (red) |
| **12. Hayat** | | | | | | | | |
| Zaidi et al, 2020 [29] | Pakistan and Afghanistan | Qualitative exploratory study<br>54 lady health workers, 42 community health workers | ● (red) | ● (red) | ● (green) | ● (green) | ● (yellow) | ● (green) |
| **13. Maternal referral mobile system** | | | | | | | | |
| Indriani et al, 2020 [30] | Indonesia | Development of technology<br>Midwives<br>(NNS) | ● (red) | ● (red) | ● (green) | ● (yellow) | ● (yellow) | ● (yellow) |

Key:

● (red) = not described ● (green) = described ● (yellow) = partially described

[1]Study design taken from description in paper

[a]Digital tools for labour monitoring
[b]Digital collection for specific birth outcomes
[c]Digital tools for use in the community
[d]Attitudes of health workers towards digital collection of birth outcomes
[e]Comparison of paper and electronic data tools
[f]Infrastructure, interoperability and sustainability

NNS= number not stated

For peer review only

| Name | Platform | Interoperability/ HIS context | Intervention content |
|---|---|---|---|
| 1. **ePartogram** | Android tablet App | Data accessible at referral facilities | Mobile partograph with automatic graphing of data, clinical decision support and auditory alerts |
| 2. **E-partograph** | Android Smartphone or tablet App | Partograph data monitored remotely | Mobile partograph with automatic graphing of labour progress and alerts for abnormalities |
| 3. **mLabour** | Mobile App | NS | Mobile partograph and labour management tool to register patients, record measurements and record delivery |
| 4. **prasavGraph** | Android Smartphone or tablet app | Birth registration with municipal authorities | Mobile partograph with digital record keeping and notifications for abnormalities |
| 5. **ASMAN** | Tablet based | Integrated into government databases | Platform with digital case sheets, dashboards, E-learning content and remote support centre |
| 6. **PPH Monitoring** | SMS based | NS | Numeric SMS protocol to report maternal demographics and birth outcomes |
| 7. **Mobile Delivery Timer** | Android smartphone | NS | Application with voice recording to verbally mark crowning, birth, crying or bag mask ventilation of baby |

For peer review only

| | | | |
|---|---|---|---|
| | App | | |
| **8. Birth defect surveillance** | Android tablet App | NS | Application to document birth defects and record birth outcomes |
| **9. RapidSMS** | Open-source App | Automatic notification to ambulance service | SMS protocols to record pregnancy, maternal and child outcomes, allows communication between CHWs and health facilities |
| **10. Data collection tool** | Smartphone App | Local health authority | Application with maternal health care forms and analytics dashboard |
| **11. Perinatal Monitoring System** | Android smartphone App | Open MRS | Tools to record maternal vital signs, clinical decision support tool, supports communication between birth attendants and medical team |
| **12. Hayat** | Smartphone App | Local health authority | Mobile application to digitalise data entry, and dashboard to visualise health reports |
| **13. Maternal referral Mobile System** | Smartphone App | Data available at referral centre | Application to communicate maternal referrals between primary health care and referral hospital |

Table 2: Technical aspect and intervention content

HIS= Health information system;
NS= Not stated. CHW= Community health worker

We identified 15 different mHealth technologies, across 12 different countries: 7 in Africa, 7 in Asia and 1 in South America. A variety of health workers were end-users of the technologies, including midwives, nurses, traditional and skilled birth attendants (TBAs and SBAs), and community health workers (CHWs).

For peer review only

Six technologies were described in more than one paper. The ePartogram and E-partograph were studied in two different settings: Tanzania and Kenya for the ePartogram [10, 11], and Bangladesh and Ethiopia for the E-partograph [12, 13]. The further studies on the other four technologies (RapidSMS, the data collection tool, perinatal monitoring app and ASMAN) were conducted in the same setting as the first published paper.

A variety of methods were used to assess the technologies, with the majority being early phase development and pilot work. Only one study reported results of a randomised control trial [28], whilst three studies were preclinical [15, 26, 30]. Sample sizes also varied between studies: the largest was 462 [13] and smallest 9 [26]. Five studies did not document the number of health workers using the technology [12, 15, 17, 20, 30].

For peer review only

## 3. Roles for the digital tools

The six themes identified were split into roles for technologies and focus of studies. The three main roles for digital tools at birth were: (1) labour monitoring; (2) recording of a specific birth outcome; (3) tools for use in the community, and studies were mainly concerned with: (1) attitudes of health workers to digital technologies; (2) comparison of paper and digital data collection; and (3) sustainability, interoperability, and infrastructure for mHealth interventions.

### 3.1. Digital tools for labour monitoring

Eight of the studies described tools that could monitor labour, all based on the WHO partograph [10-17] and shown in Table 3. Whilst the primary purpose of the partograph is to monitor labour these technologies had additional capabilities to record birth data, often to assess the impact of labour monitoring on birth outcomes.

Table 3: Digital Tools for Labour Monitoring

| Technology | Studies |
|---|---|
| ePartogram | [10, 11] |
| E-Partograph | [12, 13] |
| mLabour | [14] |
| prasavGraph | [15] |
| ASMAN | [16, 17] |

Several electronic partographs have been described. The ePartogram was developed by John Hopkins University and their NGO arm, Jhpiego. Study of its use in Tanzania showed SBAs were comfortable registering clients and entering data into the application and had a preference for the ePartogram over paper versions[10]. This was replicated in a study in Kenya, where it was also found to improve birth outcomes compared to paper partograph use [11]. Improvements in birth outcomes was also seen in studies investigating the E-Partograph in Bangladesh [12]. However, a separate study of the E-partograph in Ethiopia found only 46% of care providers would be willing to use a mobile-based E-partograph, despite 65.8% of caregivers reporting they wished to use a partograph routinely [13].

Three other technologies were identified that had a digital partograph as part of their workflow. prasavGraph is an app incorporating a partograph with other data collection, including the baby's sex, weight and condition at birth, reported in preclinical phase in New Delhi [15]. mLabour also incorporated a partograph into their labour management tool; nurses using it reported being more punctual with data entry and found the tablet form lighter and easier to record data in compared to multiple paper registers [14]. This feedback was echoed with the ASMAN application, which consists of a digital case sheet for patients, partograph, dashboard for health facilities and E-learning content, and was used in both district hospitals and community health centres [16]. Analysis of application data showed high rates of data collection for delivery in the app, at 93.7% [17].

### 3.2. Digital collection for specific birth outcomes

Three technologies were designed to report a specific outcome, but also recorded delivery details as part of the workflow. A system for community midwives to report postpartum haemorrhage (PPH) was designed in Rwanda, using mobile phone handsets and SMS protocols [18]. As well as PPH, data were collected on maternal and neonatal deaths. Ten TBAs used the technology and successfully reported 425 births and 13 incidents of PPH.

The other two studies collected data collected on tablets. The Mobile Delivery Timer was designed to measure the time between delivery and either the baby crying or initiation of bag mask ventilation, as part of a larger

For peer review only



For peer review only - http://bmjopen.bmj.com/site/about/guidelines.xhtml

'Helping Babies' Breath' study [19]. This recorded the time of delivery as well as the woman's ID and status of the newborn. 90% of birth attendants found recording data in the mobile phone either 'moderately easy to use' or 'very easy to use'. Android tablets were used in a tool to detect birth defect prevalence in Uganda [20]. All births were examined by a midwife as soon as feasible and data collected on forms designed using Open Data Kit (ODK), including both pictures of any major defects as well as quantitative data documenting maternal age, birth outcome, newborn sex and mode of delivery. The system was able to detect a birth defect prevalence of 66 per 10,000 births, but the authors noted there was no population-based data for comparison.

### 3.3. Digital tools for use in the community

Six of the tools were designed specifically to be used in the community, either by trained midwives or lay or volunteer health workers.

Two technologies used mobile phones and SMS protocols to capture community data. The PPH reporting system is one example [18], as is the RapidSMS-MCH system in Rwanda [21]. In Rwanda, volunteer community health workers were given mobile phones with eight SMS forms for reporting data, including one for delivery. The SMS was sent to a central server and could alert an ambulance in case of emergency. The technology was scaled up nationwide in 2013, and a further study found it was well accepted by both health workers and the local community [22].

A further three technologies used smartphones to record data collected by community health workers. An app was designed for data collection by midwives and health extension workers (HEWs) in Ethiopia, containing eight data collection forms for different perinatal events [23]. It also contained a scorecard and analytics dashboard to allow data monitoring by local health bureaus. The app was adapted for use in the community by using the local calendar and local language. Analysis of the records submitted found that most delivery protocols were submitted by midwives rather than HEWs, reflecting local guidance that HEWs should not assist with deliveries [24]. A similar format was seen with the Hayat app in Afghanistan and Pakistan; it was designed to digitalise data entry, and also contained a dashboard to visualise reports generated by patient encounters [29]. Community health workers were able to successfully use the app but highlighted issues such as the app not using the local language. The perinatal

For peer review only

monitoring tool used in Guatemala was adapted for community use by having audio and visual instructions and data collection through audio recordings, to be inclusive of illiterate and technology-naïve end-users [26, 27]. Similarly, to the other technologies, it collects data based on antenatal, postnatal and intrapartum encounters, combined with recording of vital signs. Once assessment is complete, the data is uploaded to the patient's OpenMRS electronic medical record. An initial feasibility study showed use was feasible among traditional birth attendants, and later randomised control testing showed that use of the app was associated with improved detection of pregnancy complications and a higher rate of referrals to medical facilities [28].

Several of the tools were designed to aid communication between health professionals. A mobile referral system was designed in Indonesia, allowing midwives to relay information about patient condition and neonatal outcomes to the referral hospital [30]. This was a feature of other platforms: the perinatal monitoring system alerted the local health facility if a complication was detected [28], and RapidSMS could automatically forward ambulance requests in an emergency [21]. ASMAN also facilitated communication, through access to a remote support centre staffed by health personnel [16].

### 4. Attitudes of health workers towards digital collection of birth outcomes

Ten studies explored the attitudes of health workers towards digital tools. Seven used focus groups or in-depth interviews [10, 11, 14, 16, 22, 23, 29] and three used questionnaires [13, 19, 24]. All but one of the studies reported that the technology was well received and preferred to conventional methods of data collection.

Four studies were specifically designed to explore the preferences of health workers [13, 16, 22, 24]. Generally they showed positive attitudes towards digital data collection; one study in Ethiopia found 87% of health workers perceived it to be useful [24], and ownership of the mobile phones was a motivating factor for using the technology for 91.3%. The RapidSMS project also gave mobiles to health workers, and whilst the technology was perceived to improve birth outcomes, the increased reporting requirement reduced the motivation of the volunteer CHWs, leaving some wanting financial compensation for the increased workload [22]. Only one study used a validated

For peer review only

tool to study health worker preferences; the Technology Acceptance Model, which explores perceived ease of use and usefulness of a technology, was used to evaluate the ASMAN application [16]. The application was reported to be easy to use and less error prone compared to paper, and improved clinical documentation by prompting users to ask for additional information as necessary.

Only one study showed a negative attitude towards a digital technology [13]. Analysis of attitudes towards the E-Partograph showed that whilst 99.6% of workers owned a mobile phone, only 46% were willing to use a mobile-based E-Partograph; however, this did not specify whether they were unwilling to use the app on their personal phone or unwilling to use the E-Partograph in general. 58.9% of those surveyed had a favourable attitude towards the partograph, and this was associated with willingness to use the E-Partograph, so the authors concluded that the results may be due to negative attitudes towards the partograph in general rather than an electronic tool specifically.

In six studies the time taken to use digital technologies was mentioned by health workers. In three, digital data collection was perceived to be a timesaver [10, 16, 29]. However, those using the ePartogram were concerned about achieving timely data entry in high volume sites or with multiple clients [11], and 78.3% of those using the Ethiopian data collection tool cited time taken as a reason for not using forms all the time [24]. With ASMAN, whilst facilities using the application alone to collect data found it reduced reporting time, some facilities required data collection electronically and on paper for verification, audit or in case of paper issues, which increased workload temporarily [16].

### 5. Comparisons of paper and electronic tools

mHealth tools were formally compared to the current standard of reporting, paper records, in three of the studies [11, 12, 25]. This was either comparing preferences for paper or electronic data collection or comparing completeness of records.

The current WHO partograph is a paper version, so studies using an electronic version often used this as a comparison. One cross-over study comparing the E-partograph to the paper version found higher user rates during

For peer review only

the electronic phase in both sites [12]. Another study found that all measurements except foetal heart rate and contractions were recorded more frequently in the ePartogram compared to paper [11]. However, SBAs found that it was more difficult to correct errors in data entry using the ePartogram, which was not an issue with the paper charts.

Similar findings were seen with the  data collection tool in Ethiopia [25]. Comparing the completeness and accuracy of patient data collection on electronic forms compared to paper, they found overall completeness was 8% higher in electronic records compared to paper. Whilst there was no difference for parameters such as patient name or age, for newborn weight recording was 20.5% higher in electronic forms. The authors speculated this could be because the electronic forms required a response to be accepted. However, they also found some workers lacked the apparatus to measure some parameters, such as scales for recording newborn weight, and had thus been discouraged from using electronic forms to record data due to mandatory reporting requirements, meaning they were more likely to use paper and reduce the completeness of paper records. The study reported that missing data on paper forms was also due lack of physical space on paper sheets.

### 6. Infrastructure, interoperability and sustainability

Few studies included information about the infrastructure necessary for mHealth technology. Some described the hardware given to study participants – for example, in Ethiopia workers were given second-hand HTC Hero smartphones, solar lamp and phone chargers [23]. They reported that of the 23 health posts in the area, only 48.9% had GPRS at the time, so only chose health posts with connectivity for the study. Network connectivity varied across the studies, with some reporting 3G being available in all facilities [10] , whereas those using the Hayat app cited a lack of connectivity as a barrier to uploading data [29]. The Hayat app had also GPS capabilities, so that health workers could be tracked during outreach visits; however, workers had to travel to the central offices once per week to upload data to the central server, which was also the case with the perinatal monitoring system in Guatemala [26].

Four studies reported capability to communicate with pre-existing health information systems (HIS) or upload data to electronic health/medical records (EHR/EMR) [16, 23, 26, 29]. The perinatal monitoring app in Guatemala



For peer review only - http://bmjopen.bmj.com/site/about/guidelines.xhtml

has been used in the community and is able to upload data to the OpenMRS EMR once assessment is complete [26]. However, in the randomised control trial of the application, this feature was not mentioned [28]. The data collection project in Ethiopia discussed uploading their data to EMRs but stated that improvements in patient identification would be needed for this to be successful [23]. However, the Ethiopian data collection app, the Hayat app [29], and the ASMAN package [16] all created a dashboard from data collection reports submitted, which allowed local health bureaus to view the data. Beside connecting to a HIS, prasavGraph reported that their data could be used to record births with municipal authorities, but this has not progressed beyond prototype testing [15].

Table 4 contains a summary of user feedback, contextual adaptability and limitations for delivery at scale. Not all the studies considered sustainability of the intervention, or the feasibility of scaling technology up. Scaling-up was discussed with regards to ASMAN, detailing a transition package to move past the pilot phase involving transferring the application to the states' servers and integrating ASMAN data fields into government bases [16]. However at the time of writing, only RapidSMS-MCH had been scaled up, to involve all CHWs across Rwanda [22].

For peer review only

Table 4: Limitations and contextual adaptability

| Name | User feedback | Limitations for delivery at scale | Contextual adaptability |
|---|---|---|---|
| **1. ePartogram** | | | |
| | • Improved decision making<br>• Data entry simple and user-friendly | • Concerns about time for data entry in high-volume labour wards | Feedback session with SBAs done as part of development process |
| **2. E-partograph** | | | |
| | • 44% were willing to use<br>• 58.9% had favourable attitudes towards the partograph | • Obstetric care providers less willing to use | NS |
| **3. mLabour** | | | |
| | • Users preferred tablet format to paper<br>• Requests for additional functionality, e.g. tracking medications | NS | Some features of app customised for context of specific hospital, not wider use |
| **4. prasavGraph** | | | |
| | NS | NS | Designed for use in settings with poor network connection |
| **5. ASMAN** | | | |
| | • Technology easy to use<br>• Perceived improvement in job performance | • Technology and internet issues<br>• High caseloads prevented all fields being completed | E-learning written in English and Hindi with audio, visual and readable formats |
| **6. PPH Monitoring** | | | |
| | NS | • Poor network and power outages<br>• Lack of remuneration | Training given in English and native language |
| **7. Mobile Delivery Timer** | | | |
| | • 90% found application easy to use<br>• 70% felt app was beneficial | NS | Android operating system chosen as functions on low-cost hardware |
| **8. Birth defect surveillance** | | | |

For peer review only

| | | |
|---|---|---|
| NS | • System does not capture babies born outside of urban facilities | NS |
| **9. RapidSMS** | | |
| • Workers reported being more pro-active in finding pregnant women<br>• Requests for financial compensation | • High initial cost<br>• Lack of equipment prevented all data being collected | Mobile phones provided to CHWs to reduce their costs |
| **10. Data collection tool** | | |
| • 52.2% said forms were comprehensive<br>• 87% found electronic forms helpful<br>• Preferred electronic forms to paper | • High cost of covering phone airtime | Provided with solar lamp to charge smartphone |
| **11. Perinatal Monitoring System** | | |
| • System was easy to operate with potential benefit | • Unfamiliarity with technology<br>• Low quality ultrasound recordings | Audio and visual instructions used to adapt user interface for illiterate users |
| **12. Hayat** | | |
| • App use feasible and improved efficiency<br>• Frustrated at inability to edit entries | • CHWs needed to travel long distances each week to sync app data with central server | NS |
| **13. Maternal referral Mobile System** | | |
| NS | • Bugs in application | Focus group interviews conducted prior to development |

NS= Not stated

Most of the technologies used Android hardware, which is widely available in LMICs and is relatively low cost; one study cited its use as a way to increase sustainability [19]. The sustainability of the perinatal monitoring system in Guatemala was assessed in relation to its cost which was less than $50 for the equipment, while its software was open source [27]. The US engineers involved in the development of the app also spent time training engineers in Guatemala to be able to deal with software and hardware issues relating to the technology [27]. Issues in scaling up mHealth use were highlighted in the Ethiopian study where workers were given cell phones; they



For peer review only - http://bmjopen.bmj.com/site/about/guidelines.xhtml

said that the cost of airtime was high owing to workers using phones for personal and study purposes. They suggested that the intervention would be more sustainable if a mechanism could be found either to provide free airtime for uploading protocols or to restrict airtime to study protocols [24].

## *Discussion*

We identified 21 studies describing 15 mHealth technologies that could collect data in the delivery room. Studies showed that data collection at delivery is both feasible and acceptable to healthcare providers. Of the six themes we identified, three were the primary purposes technologies were designed for: labour monitoring, collection of a specific birth outcome and data collection in the community. Whilst no technology was specifically designed to record birth outcomes in the delivering room, our findings suggest that use of such a tool would be feasible and acceptable.

All but one of the studies found that digital data collection was preferred to paper methods. Digital technologies were reported to be easy to use [16, 19], user-friendly [11] and perceived to improve worker efficiency [29] and birth outcomes [22]. Health workers also preferred the digital format compared to paper methods, noting them to be less bulky to carry and easier to access [14, 29]. It was also reported that digital collection could improve the number of data items reported, by incorporating mandatory data entry fields [16, 22].

We found that the time taken to input data can be a barrier. In some studies, this study design requiring paper and electronic entry rather than issues with the tool itself [16, 24]. However, we also found evidence that health workers were concerned about time to input data [23], particularly in facilities caring for large numbers of patients [11]. Digital tools can mandate reporting of more items, and whilst this is useful for data collection and research, it can be time-consuming for health workers and reduce motivation to record data [22]. An mHealth application that did not meet criteria for inclusion, mClinic, sought end-user feedback on items for data reporting, finding that users only wanted items that would be included in reports and did not want free-text for reporting of subjective data [31]. Clearly, end-user involvement in designing data collection protocols is important to ensure that each data item is perceived to be useful for health worker's practice.

For peer review only


For peer review only - http://bmjopen.bmj.com/site/about/guidelines.xhtml

We found that there are several gaps in reporting of interventions in the published literature. One of the items in the mERA checklist, developed by the WHO, is ‘Interoperability/Health information systems (HIS) context’, which involves ‘describing how mHealth intervention can integrate into existing health information systems’ [9]. Our review found few studies describing how the mHealth tool would integrate with the existing HIS; only the perinatal monitoring app appeared to upload data directly to patient’s medical records app [26]. Another item in the mERA checklist is ‘Cost Assessment’, and again only the perinatal monitoring app mentioned the cost of the hardware required to run the technology [26]. Consideration of both cost and interoperability are crucial for the sustainability [32], so the lack of reporting in the literature highlights the need for both standardised reporting of interventions and for authors to consider these factors when designing and testing new technology.

Few tools were identified with the capability to record births in both facilities and in the community. Whilst several of the community-based technologies could communicate data to facilities, only the Ethiopian data collection tool was used to collect data by both health workers in the community and midwives in health centres [23]. Since the proportion of non-facility births in many LMICs is high [1], a digital birth register would need to be designed for use in both the community and facilities to ensure maximum coverage of births.

To our knowledge, no other review has considered the use of mHealth specifically in the delivering room, or its potential to replace paper-based delivery registers. However, our review is in agreement with other reviews that there is a lack of high-quality evidence and robust study design to determine the efficacy and effectiveness of mHealth interventions in maternal health care [5, 33, 34]. Of the studies we identified, only one was a randomised-control trial, and of the 15 technologies only six were evaluated by more than one study. With regards to the purpose of evaluation, only two studies formally assessed the accuracy of digital data collection compared to paper methods [11, 25], and only six studies explored the time needed for digital input relative to paper recording [10, 11, 16, 24, 29] . The lack of quality evidence creates a barrier to scaling up technologies and emphasises the need for pilot studies to be followed-up with studies that investigate scalability [32].



For peer review only - http://bmjopen.bmj.com/site/about/guidelines.xhtml

Our own review has its strengths and limitations. Whilst we used a comprehensive search strategy, we only included peer-reviewed articles, omitting potentially relevant data published in the grey literature. We were also limited to studies published in English. Publication bias was also a limitation – whilst we included all available studies that were captured in our search, there may be other relevant technologies that have not been described in peer-reviewed articles.

In conclusion, this review identified several mHealth technologies capable of recording birth outcomes in the delivering room. Whilst none of the technologies were specifically designed for this purpose, the evidence suggests use of digital delivery registers would be both feasible and acceptable to health workers. Few of the tools identified had adequate reporting of interoperability and sustainability, and more research is needed to determine the efficacy and effectiveness of their use.

***ACKNOWLEDGEMENTS***

We are grateful to librarian N. Thomas for helping build the initial search strategy.

***CONTRIBUTORSHIP STATEMENT***

JEH, CR, AK, AM, DL, FC conceived the review idea. LGC carried out the search and LGC and GK screened and selected articles. LGC wrote the manuscript with input from all authors. All authors approved the manuscript prior to submission.

***DATA SHARING STATEMENT***

All data are included in the manuscript.

***FUNDING***

There was no specific funding for this project. JEH is funded by a UKRI Future Leaders Fellowship.

***ETHICS APPROVAL***

This study did not involve human or animal subjects. Ethics approval is not applicable for this article.

For peer review only

## *COMPETING INTERESTS STATMEENT*

The authors declare that there were no conflicts of interest.

## *AUTHOR REFLEXIVITY STATEMENT*

The original concept for this systematic review was co-developed equally by the authors from high and low-income country settings to address an identified priority from the authors from Tanzania and Malawi. The protocol was jointly developed and the search strategy and data extraction were performed by HIC researchers, with support from LIC researchers. The LIC researchers contributed to interpretation of themes and ensuring contextual relevance. All authors read and approved the final version of the manuscript. The first (LGC) and last author (JEH) are both HIC authors, as the first author is a student who has been supervised by the senior author.

## *APPENDIX*

The search strategies can be found in the appendix.

## *REFERENCES*


1. UNICEF, *Maternal and Newborn Health Coverage Database* UNICEF, Editor. 2021.
2. WHO, UNFPA, World Bank Group and UNPD *Trends in estimates of maternal mortality ratio (MMR; maternal deaths per 100,000 live births) maternal deaths and lifetime risk of maternal death, 2000-2017* 2021: https://data.unicef.org/topic/maternal-health/maternal-mortality/.
3. Pew Research Centre, *Smartphone Ownership Is Growing Rapidly Around the World, but Not Always Equally* 2019.
4. WHO, *mHealth: Use of appropriate digital technologies for public health* 2018.
5. Colaci, D., S. Chaudhri, and A. Vasan, *mHealth Interventions in Low-Income Countries to Address Maternal Health: A Systematic Review.* Ann Glob Health, 2016. **82**(5): p. 922-935.
6. Tricco, A.C.e.a., *PRISMA Extension for Scoping Reviews (PRISMA-ScR): Checklist and Explanation.* Annals of Internal Medicine, 2018. **169**(7): p. 467-473.
7. Peters, M.G., C; et al *Chapter 11: Scoping Reviews* in *JBI Manual for Evidence Synthesis* E.M. Aromataris, Z, Editor. 2020.
8. The World Bank. *Low & middle income* 2021 [cited 2021 21/12/2021]; Available from: https://data.worldbank.org/income-level/low-and-middle-income.


For peer review only

9. Agarwal, S., et al., *Guidelines for reporting of health interventions using mobile phones: mobile health (mHealth) evidence reporting and assessment (mERA) checklist.* BMJ, 2016. **352**: p. i1174.
10. Litwin, L.E., et al., *Use of an electronic Partograph: feasibility and acceptability study in Zanzibar, Tanzania.* BMC Pregnancy Childbirth, 2018. **18**(1): p. 147.
11. Sanghvi, H., et al., *Effectiveness of an Electronic Partogram: A Mixed-Method, Quasi-Experimental Study Among Skilled Birth Attendants in Kenya.* Global Health: Science and Practice, 2019. **7**(4): p. 521-539.
12. Rahman, A., et al., *Feasibility and effectiveness of electronic vs. paper partograph on improving birth outcomes: A prospective crossover study design.* PLoS One, 2019. **14**(10): p. e0222314.
13. Tadesse, Y., et al., *Willingness to Use Mobile based e-Partograph and Associated Factors among Care Providers in North Gondar Zone, Northwest Ethiopia.* Online journal of public health informatics, 2019. **11**(2): p. e10-e10.
14. Schweers, J., et al., *mLabour: Design and Evaluation of a Mobile Partograph and Labor Ward Management Application.* Procedia engineering, 2016. **159**: p. 35-43.
15. Singh, S., et al. *prasavGraph: An Android-based e-Partograph.* in *2016 International Conference on Systems in Medicine and Biology (ICSMB).* 2016.
16. Usmanova, G., et al., *Acceptability and Barriers to Use of the ASMAN Provider-Facing Electronic Platform for Peripartum Care in Public Facilities in Madhya Pradesh and Rajasthan, India: A Qualitative Study Using the Technology Acceptance Model-3.* International journal of environmental research and public health, 2020. **17**(22): p. 8333.
17. Usmanova, G., et al., *The role of digital clinical decision support tool in improving quality of intrapartum and postpartum care: experiences from two states of India.* BMC Pregnancy & Childbirth, 2021. **21**(1): p. 1-12.
18. Andreatta, P., et al., *Using cell phones to collect postpartum hemorrhage outcome data in rural Ghana.* Int J Gynaecol Obstet, 2011. **113**(2): p. 148-51.
19. Somannavar, M.S., et al., *Evaluating time between birth to cry or bag and mask ventilation using mobile delivery room timers in India: the NICHD Global Network's Helping Babies Breathe Trial.* BMC Pediatrics, 2015. **15**(93).
20. Mumpe-Mwanja, D., et al., *A hospital-based birth defects surveillance system in Kampala, Uganda.* BMC Pregnancy Childbirth, 2019. **19**(1): p. 372.
21. Ngabo, F., et al., *Designing and Implementing an Innovative SMS-based alert system (RapidSMS-MCH) to monitor pregnancy and reduce maternal and child deaths in Rwanda.* Pan Afr Med J, 2012. **13**: p. 31.
22. Musabyimana, A., et al., *Assessing the perspectives of users and beneficiaries of a community health worker mHealth tracking system for mothers and children in Rwanda.* PLoS One, 2018. **13**(6): p. e0198725.
23. Little, A., et al., *Meeting Community Health Worker Needs for Maternal Health Care Service Delivery Using Appropriate Mobile Technologies in Ethiopia.* PLOS ONE, 2013. **8**(10): p. e77563.
24. Medhanyie, A.A., et al., *Health workers' experiences, barriers, preferences and motivating factors in using mHealth forms in Ethiopia.* Human Resources for Health, 2015. **13**(1): p. 2.


For peer review only



For peer review only - http://bmjopen.bmj.com/site/about/guidelines.xhtml

25. Medhanyie, A.A., et al., *Quality of routine health data collected by health workers using smartphone at primary health care in Ethiopia.* International Journal of Medical Informatics, 2017. **101**: p. 9-14.

26. Stroux, L., et al., *An mHealth monitoring system for traditional birth attendant-led antenatal risk assessment in rural Guatemala.* Journal of medical engineering & technology, 2016. **40**(7-8): p. 356-371.

27. Martinez, B., et al., *Agile Development of a Smartphone App for Perinatal Monitoring in a Resource-Constrained Setting.* J Health Inform Dev Ctries, 2017. **11**(1).

28. Martinez, B., et al., *mHealth intervention to improve the continuum of maternal and perinatal care in rural Guatemala: a pragmatic, randomized controlled feasibility trial.* Reprod Health, 2018. **15**(1): p. 120.

29. Zaidi, S., et al., *Operability, Usefulness, and Task-Technology Fit of an mHealth App for Delivering Primary Health Care Services by Community Health Workers in Underserved Areas of Pakistan and Afghanistan: Qualitative Study.* J Med Internet Res, 2020. **22**(9): p. e18414.

30. Indriani, D., et al., *The maternal referral mobile application system for minimizing the risk of childbirth.* J Public Health Res, 2020. **9**(2): p. 1813.

31. Velez, O., et al., *A usability study of a mobile health application for rural Ghanaian midwives.* J Midwifery Womens Health, 2014. **59**(2): p. 184-91.

32. Tomlinson, M.R.-B., MJ; Swartz, L; Tsai, AC; , *Scaling Up mHealth: Where Is the Evidence?* . PLoS Medicine 2013. **10(2)**.

33. Agarwal, S.P., HB; Long, LA; Labrique, AB *Evidence on feasibility and effective use of mHealth strategies by frontline health workers in developing countries: systematic review.* Tropical Medicine and International Health, 2015. **20**(8): p. 1003-1014.

34. Balikuddembe, M.S., et al., *Computerized Childbirth Monitoring Tools for Health Care Providers Managing Labor: A Scoping Review.* JMIR Med Inform, 2017. **5**(2): p. e14.


For peer review only

FIGURE LEGEND:

Figure 1: MEDLINE, CINAHL and Global Health were searched for relevant references. These were first screened by citation then full text. Articles selected for inclusion were then hand-searched for relevant references.

FIGURE CAPTION:

Figure 1: *Figure 1: screening and selection of articles*

For peer review only - http://bmjopen.bmj.com/site/about/guidelines.xhtml

1. UNICEF, *Maternal and Newborn Health Coverage Database* UNICEF, Editor. 2021.
2. WHO, U., UNFPA, World Bank Group and UNPD *Trends in estimates of maternal mortality ratio (MMR; maternal deaths per 100,000 live births) maternal deaths and lifetime risk of maternal death, 2000-2017* 2021: https://data.unicef.org/topic/maternal-health/maternal-mortality/.
3. Centre, P.R., *Smartphone Ownership Is Growing Rapidly Around the World, but Not Always Equally* 2019.
4. WHO, *mHealth: Use of appropriate digital technologies for public health* 2018.
5. Colaci, D., S. Chaudhri, and A. Vasan, *mHealth Interventions in Low-Income Countries to Address Maternal Health: A Systematic Review.* Ann Glob Health, 2016. **82**(5): p. 922-935.
6. Tricco, A.C.e.a., *PRISMA Extension for Scoping Reviews (PRISMA-ScR): Checklist and Explanation.* Annals of Internal Medicine, 2018. **169**(7): p. 467-473.
7. Peters, M.G., C; et al *Chapter 11: Scoping Reviews* in *JBI Manual for Evidence Synthesis* E.M. Aromataris, Z, Editor. 2020.
8. Bank, T.W. *Low & middle income* 2021 [cited 2021 21/12/2021]; Available from: https://data.worldbank.org/income-level/low-and-middle-income.
9. Agarwal, S., et al., *Guidelines for reporting of health interventions using mobile phones: mobile health (mHealth) evidence reporting and assessment (mERA) checklist.* BMJ, 2016. **352**: p. i1174.
10. Litwin, L.E., et al., *Use of an electronic Partograph: feasibility and acceptability study in Zanzibar, Tanzania.* BMC Pregnancy Childbirth, 2018. **18**(1): p. 147.
11. Sanghvi, H., et al., *Effectiveness of an Electronic Partogram: A Mixed-Method, Quasi-Experimental Study Among Skilled Birth Attendants in Kenya.* Global Health: Science and Practice, 2019. **7**(4): p. 521-539.
12. Rahman, A., et al., *Feasibility and effectiveness of electronic vs. paper partograph on improving birth outcomes: A prospective crossover study design.* PLoS One, 2019. **14**(10): p. e0222314.
13. Tadesse, Y., et al., *Willingness to Use Mobile based e-Partograph and Associated Factors among Care Providers in North Gondar Zone, Northwest Ethiopia.* Online journal of public health informatics, 2019. **11**(2): p. e10-e10.
14. Schweers, J., et al., *mLabour: Design and Evaluation of a Mobile Partograph and Labor Ward Management Application.* Procedia engineering, 2016. **159**: p. 35-43.
15. Singh, S., et al. *prasavGraph: An Android-based e-Partograph.* in *2016 International Conference on Systems in Medicine and Biology (ICSMB).* 2016.
16. Usmanova, G., et al., *Acceptability and Barriers to Use of the ASMAN Provider-Facing Electronic Platform for Peripartum Care in Public Facilities in Madhya Pradesh and Rajasthan, India: A Qualitative Study Using the Technology Acceptance Model-3.* International journal of environmental research and public health, 2020. **17**(22): p. 8333.
17. Usmanova, G., et al., *The role of digital clinical decision support tool in improving quality of intrapartum and postpartum care: experiences from two states of India.* BMC Pregnancy & Childbirth, 2021. **21**(1): p. 1-12.
18. Andreatta, P., et al., *Using cell phones to collect postpartum hemorrhage outcome data in rural Ghana.* Int J Gynaecol Obstet, 2011. **113**(2): p. 148-51.
19. Somannavar, M.S., et al., *Evaluating time between birth to cry or bag and mask ventilation using mobile delivery room timers in India: the NICHD Global Network's Helping Babies Breathe Trial.* BMC Pediatrics, 2015. **15**(93).
20. Mumpe-Mwanja, D., et al., *A hospital-based birth defects surveillance system in Kampala, Uganda.* BMC Pregnancy Childbirth, 2019. **19**(1): p. 372.
21. Ngabo, F., et al., *Designing and Implementing an Innovative SMS-based alert system (RapidSMS-MCH) to monitor pregnancy and reduce maternal and child deaths in Rwanda.* Pan Afr Med J, 2012. **13**: p. 31.


For peer review only

22. Musabyimana, A., et al., *Assessing the perspectives of users and beneficiaries of a community health worker mHealth tracking system for mothers and children in Rwanda.* PLoS One, 2018. **13**(6): p. e0198725.
23. Little, A., et al., *Meeting Community Health Worker Needs for Maternal Health Care Service Delivery Using Appropriate Mobile Technologies in Ethiopia.* PLOS ONE, 2013. **8**(10): p. e77563.
24. Medhanyie, A.A., et al., *Health workers' experiences, barriers, preferences and motivating factors in using mHealth forms in Ethiopia.* Human Resources for Health, 2015. **13**(1): p. 2.
25. Medhanyie, A.A., et al., *Quality of routine health data collected by health workers using smartphone at primary health care in Ethiopia.* International Journal of Medical Informatics, 2017. **101**: p. 9-14.
26. Stroux, L., et al., *An mHealth monitoring system for traditional birth attendant-led antenatal risk assessment in rural Guatemala.* Journal of medical engineering & technology, 2016. **40**(7-8): p. 356-371.
27. Martinez, B., et al., *Agile Development of a Smartphone App for Perinatal Monitoring in a Resource-Constrained Setting.* J Health Inform Dev Ctries, 2017. **11**(1).
28. Martinez, B., et al., *mHealth intervention to improve the continuum of maternal and perinatal care in rural Guatemala: a pragmatic, randomized controlled feasibility trial.* Reprod Health, 2018. **15**(1): p. 120.
29. Zaidi, S., et al., *Operability, Usefulness, and Task-Technology Fit of an mHealth App for Delivering Primary Health Care Services by Community Health Workers in Underserved Areas of Pakistan and Afghanistan: Qualitative Study.* J Med Internet Res, 2020. **22**(9): p. e18414.
30. Indriani, D., et al., *The maternal referral mobile application system for minimizing the risk of childbirth.* J Public Health Res, 2020. **9**(2): p. 1813.
31. Velez, O., et al., *A usability study of a mobile health application for rural Ghanaian midwives.* J Midwifery Womens Health, 2014. **59**(2): p. 184-91.
32. Tomlinson, M.R.-B., MJ; Swartz, L; Tsai, AC; , *Scaling Up mHealth: Where Is the Evidence? .* PLoS Medicine 2013. **10(2).**
33. Agarwal, S.P., HB; Long, LA; Labrique, AB *Evidence on feasibility and effective use of mHealth strategies by frontline health workers in developing countries: systematic review.* Tropical Medicine and International Health, 2015. **20**(8): p. 1003-1014.
34. Balikuddembe, M.S., et al., *Computerized Childbirth Monitoring Tools for Health Care Providers Managing Labor: A Scoping Review.* JMIR Med Inform, 2017. **5**(2): p. e14.

For peer review only

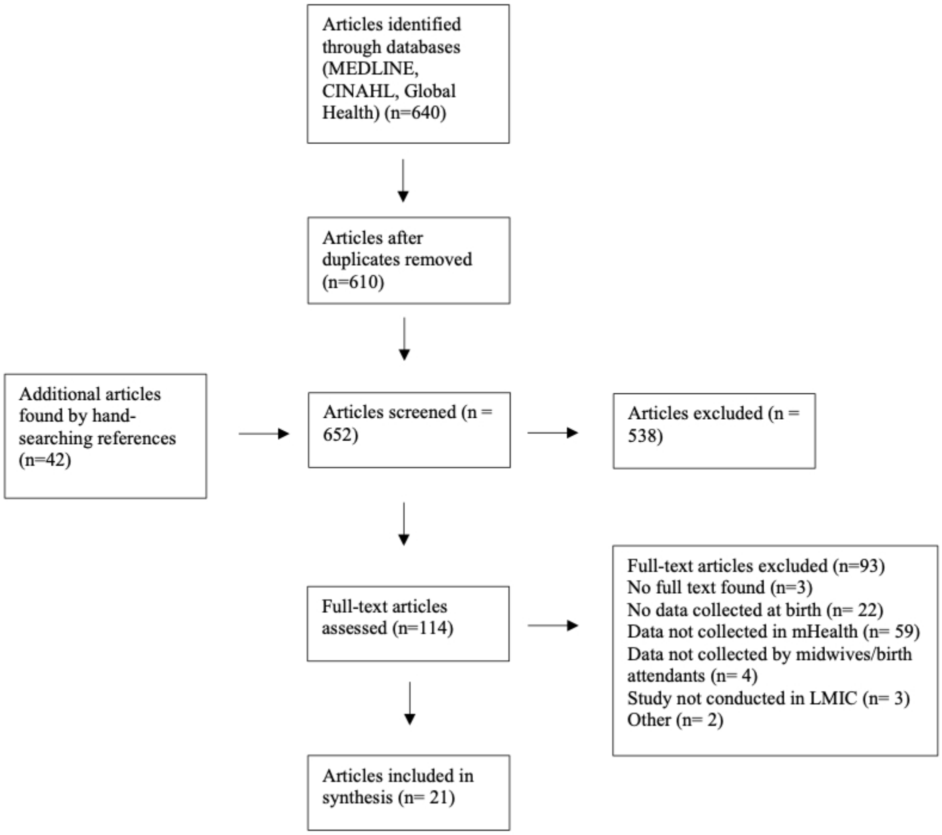


FIGURE LEGEND:
Figure 1: MEDLINE, CINAHL and Global Health were searched for relevant references. These were first screened by citation then full text. Articles selected for inclusion were then hand-searched for relevant references.

FIGURE CAPTION:
Figure 1: Figure 1: screening and selection of articles

295x248mm (72 x 72 DPI)

***APPENDIX***

Search strategies:

PubMed:

((parturition* OR childbirth* OR labour*) OR ("Parturition"[Mesh])) AND ((((("Electronic Health Records"[Mesh]) OR ( "Mobile Applications"[Mesh] OR "Cell Phone"[Mesh] )) OR ( "Cell Phone Use"[Mesh] OR "Computers, Handheld"[Mesh] )) OR (mobile phone* or mobile* or mhealth* or electronic* or smartphone* or iPhone* or iPad* or tablet computer* or cellphone* or m-health* or ehealth* or e-health* or cell phone* or handheld computer*)) AND ((Africa or Caribbean or "West Indies" or "South America" or "Latin America" or "Central America" or Afghanistan or Albania or Algeria or Angola or "American Samoa" or Armenia or Armenian or Azerbaijan or Bangladesh or Benin or Byelarus or Byelorussian or Belarus or Belorussian or Belorussia or Belize or Bhutan or Bolivia or Bosnia or Herzegovina or Hercegovina or Botswana or Brazil or Brasil or Bulgaria or "Burkina Faso" or "Burkina Fasso" or "Upper Volta" or Burundi or Urundi or Cambodia or "Khmer Republic" or Kampuchea or Cameroon or Cameroons or Cameron or Camerons or "Cape Verde" or "Central African Republic" or Chad or China or Colombia or Comoros or "Comoro Islands" or Comores or Mayotte or Congo or Zaire or "Costa Rica" or "Cote d'Ivoire" or "Ivory Coast" or Cuba or Djibouti or "French Somaliland" or Dominica or "Dominican Republic" or "East Timor" or "East Timur" or "Timor Leste" or Ecuador or Egypt or "United Arab Republic" or "El Salvador" or Eritrea or Ethiopia or Fiji or Gabon or "Gabonese Republic" or Gambia or Gaza or "Georgia Republic" or "Georgian Republic" or Ghana or "Gold Coast" or Grenada or Guatemala or Guinea or Guinea-Bissau or Guam or Guiana or Guyana or Haiti or Honduras or India or Maldives or Indonesia or Iran or Iraq or Jamaica or Jordan or Kazakhstan or Kazakh or Kenya or Kiribati or Korea or Kosovo or Kyrgyzstan or Kirghizia or "Kyrgyz Republic" or Kirghiz or Kirgizstan or "Lao PDR" or Laos or Lebanon or Lesotho or Basutoland or Liberia or Libya or Macedonia or Madagascar or "Malagasy Republic" or Malaysia or Malaya or Malay or Sabah or Sarawak or Malawi or Nyasaland or Mali or "Marshall Islands" or Mauritania or Mauritius or "Agalega Islands" or Mexico or Micronesia or "Middle East" or Moldova or Moldovia or Moldovian or Mongolia or Montenegro or Morocco or Ifni or Mozambique or Myanmar or Myanma or Burma or Namibia or Nepal or "Netherlands Antilles" or Nicaragua or Niger or Nigeria or Pakistan or Palau or Palestine or Panama or "Papua New Guinea" or Paraguay or Peru or Philippines or Philipines or Phillipines or Phillippines or Romania or Rumania or Roumania or Rwanda or Ruanda or "Saint Lucia" or "St Lucia" or "Saint Vincent" or "St Vincent" or Grenadines or Samoa or "Samoan Islands" or "Navigator Island" or "Navigator Islands" or "Sao Tome" or Senegal or Serbia or "Sierra Leone" or Sri Lanka or Ceylon or "Solomon Islands" or Somalia or Sudan or Suriname or Surinam or Swaziland or Syria or Principe or Tajikistan or Tadzhikistan or Tadjikistan or Tadzhik or Tanzania or Thailand or Timor-Leste or Togo or "Togolese Republic" or Tonga or Tunisia or Turkey or Turkmenistan or Turkmen or Tuvalu or Uganda or Ukraine or Uzbekistan or Uzbek or Vanuatu or "New Hebrides" or Vietnam or "Viet Nam" or "West Bank" or Yemen or Zambia or Zimbabwe or Rhodesia or developing countr* or less developed countr* or under developed countr* or underdeveloped countr* or middle income countr* or low income countr* or deprived countr* or underserved countr* or under served countr* or poor countr* or developing nation* or less developed nation* or under developed nation* or underdeveloped nation* or middle income

For peer review only

nation* or low income nation* or deprived nation* or underserved nation* or under served nation* or poor nation* or LMIC or lmics or third world* or lami countr* or transitional countr* or "global health") OR ("Developing Countries"[Mesh]))) AND (("Midwifery"[Mesh]) OR (midwife* or midwives or birth attendant* or doula* or childbirth assistant*)))

Global Health:

((mobile phone* or mobile* or mhealth* or electronic* or smartphone* or iPhone* or iPad* or tablet computer* or cellphone* or m-health* or ehealth* or e-health* or cell phone* or handheld computer).mp. [mp=abstract, title, original title, broad terms, heading words, identifiers, cabicodes] OR (mobile telephones/ OR mobile equipment/)) AND ((midwife* or midwives or birth attendant* or doula* or childbirth assistant*).mp. [mp=abstract, title, original title, broad terms, heading words, identifiers, cabicodes] OR ((traditional birth attendants or midwives).sh)) AND ((childbirth/) OR ((childbirth* or labour*).mp. [mp=abstract, title, original title, broad terms, heading words, identifiers, cabicodes]) AND ((Developing Countries/) OR (Africa or Caribbean or "West Indies" or "South America" or "Latin America" or "Central America" or Afghanistan or Albania or Algeria or Angola or "American Samoa" or Armenia or Armenian or Azerbaijan or Bangladesh or Benin or Byelarus or Byelorussian or Belarus or Belorussian or Belorussia or Belize or Bhutan or Bolivia or Bosnia or Herzegovina or Hercegovina or Botswana or Brazil or Brasil or Bulgaria or "Burkina Faso" or "Burkina Fasso" or "Upper Volta" or Burundi or Urundi or Cambodia or "Khmer Republic" or Kampuchea or Cameroon or Cameroons or Cameron or Camerons or "Cape Verde" or "Central African Republic" or Chad or China or Colombia or Comoros or "Comoro Islands" or Comores or Mayotte or Congo or Zaire or "Costa Rica" or "Cote d'Ivoire" or "Ivory Coast" or Cuba or Djibouti or "French Somaliland" or Dominica or "Dominican Republic" or "East Timor" or "East Timur" or "Timor Leste" or Ecuador or Egypt or "United Arab Republic" or "El Salvador" or Eritrea or Ethiopia or Fiji or Gabon or "Gabonese Republic" or Gambia or Gaza or "Georgia Republic" or "Georgian Republic" or Ghana or "Gold Coast" or Grenada or Guatemala or Guinea or Guinea-Bissau or Guam or Guiana or Guyana or Haiti or Honduras or India or Maldives or Indonesia or Iran or Iraq or Jamaica or Jordan or Kazakhstan or Kazakh or Kenya or Kiribati or Korea or Kosovo or Kyrgyzstan or Kirghizia or "Kyrgyz Republic" or Kirghiz or Kirgizstan or "Lao PDR" or Laos or Lebanon or Lesotho or Basutoland or Liberia or Libya or Macedonia or Madagascar or "Malagasy Republic" or Malaysia or Malaya or Malay or Sabah or Sarawak or Malawi or Nyasaland or Mali or "Marshall Islands" or Mauritania or Mauritius or "Agalega Islands" or Mexico or Micronesia or "Middle East" or Moldova or Moldovia or Moldovian or Mongolia or Montenegro or Morocco or Ifni or Mozambique or Myanmar or Myanma or Burma or Namibia or Nepal or "Netherlands Antilles" or Nicaragua or Niger or Nigeria or Pakistan or Palau or Palestine or Panama or "Papua New Guinea" or Paraguay or Peru or Philippines or Philipines or Phillipines or Phillippines or Romania or Rumania or Roumania or Rwanda or Ruanda or "Saint Lucia" or "St Lucia" or "Saint Vincent" or "St Vincent" or Grenadines or Samoa or "Samoan Islands" or "Navigator Island" or "Navigator Islands" or "Sao Tome" or Senegal or Serbia or "Sierra Leone" or Sri Lanka or Ceylon or "Solomon Islands" or Somalia or Sudan or Suriname or Surinam or Swaziland or Syria or Principe or Tajikistan or Tadzhikistan or Tadjikistan or Tadzhik or Tanzania or Thailand or Timor-Leste or Togo or "Togolese Republic" or Tonga or Tunisia or Turkey or Turkmenistan or Turkmen or Tuvalu or Uganda or Ukraine or Uzbekistan or Uzbek or Vanuatu or "New Hebrides" or Vietnam or "Viet Nam" or "West Bank" or Yemen or Zambia or

For peer review only

Zimbabwe or Rhodesia or developing countr* or less developed countr* or under developed countr* or underdeveloped countr* or middle income countr* or low income countr* or deprived countr* or underserved countr* or under served countr* or poor countr* or developing nation* or less developed nation* or under developed nation* or underdeveloped nation* or middle income nation* or low income nation* or deprived nation* or underserved nation* or under served nation* or poor nation* or LMIC or lmics or third world* or lami countr* or transitional countr* or "global health").mp. [mp=abstract, title, original title, broad terms, heading words, identifiers, cabicodes])

CINAHL:

( mh:("Midwifery") OR (midwife* or midwives or birth attendant* or doula* or childbirth assistant*)) AND (mh:("Labour") OR (childbirth* OR labour*)) AND (mh:("Cellular Phone" OR "Mobile Applications" OR "Electronic Health Records" OR "Computers, Hand-Held" OR "Telemedicine") OR (mobile phone* or mobile* or mhealth* or electronic* or smartphone* or iPhone* or iPad* or tablet computer* or cellphone* or m-health* or ehealth* or e-health* or cell phone* or handheld computer*)) AND (mh:("Developing Countries") OR (Africa or Caribbean or "West Indies" or "South America" or "Latin America" or "Central America" or Afghanistan or Albania or Algeria or Angola or "American Samoa" or Armenia or Armenian or Azerbaijan or Bangladesh or Benin or Byelarus or Byelorussian or Belarus or Belorussian or Belorussia or Belize or Bhutan or Bolivia or Bosnia or Herzegovina or Hercegovina or Botswana or Brazil or Brasil or Bulgaria or "Burkina Faso" or "Burkina Fasso" or "Upper Volta" or Burundi or Urundi or Cambodia or "Khmer Republic" or Kampuchea or Cameroon or Cameroons or Cameron or Camerons or "Cape Verde" or "Central African Republic" or Chad or China or Colombia or Comoros or "Comoro Islands" or Comores or Mayotte or Congo or Zaire or "Costa Rica" or "Cote d'Ivoire" or "Ivory Coast" or Cuba or Djibouti or "French Somaliland" or Dominica or "Dominican Republic" or "East Timor" or "East Timur" or "Timor Leste" or Ecuador or Egypt or "United Arab Republic" or "El Salvador" or Eritrea or Ethiopia or Fiji or Gabon or "Gabonese Republic" or Gambia or Gaza or "Georgia Republic" or "Georgian Republic" or Ghana or "Gold Coast" or Grenada or Guatemala or Guinea or Guinea-Bissau or Guam or Guiana or Guyana or Haiti or Honduras or India or Maldives or Indonesia or Iran or Iraq or Jamaica or Jordan or Kazakhstan or Kazakh or Kenya or Kiribati or Korea or Kosovo or Kyrgyzstan or Kirghizia or "Kyrgyz Republic" or Kirghiz or Kirgizstan or "Lao PDR" or Laos or Lebanon or Lesotho or Basutoland or Liberia or Libya or Macedonia or Madagascar or "Malagasy Republic" or Malaysia or Malaya or Malay or Sabah or Sarawak or Malawi or Nyasaland or Mali or "Marshall Islands" or Mauritania or Mauritius or "Agalega Islands" or Mexico or Micronesia or "Middle East" or Moldova or Moldovia or Moldovian or Mongolia or Montenegro or Morocco or Ifni or Mozambique or Myanmar or Myanma or Burma or Namibia or Nepal or "Netherlands Antilles" or Nicaragua or Niger or Nigeria or Pakistan or Palau or Palestine or Panama or "Papua New Guinea" or Paraguay or Peru or Philippines or Philipines or Phillipines or Phillippines or Romania or Rumania or Roumania or Rwanda or Ruanda or "Saint Lucia" or "St Lucia" or "Saint Vincent" or "St Vincent" or Grenadines or Samoa or "Samoan Islands" or "Navigator Island" or "Navigator Islands" or "Sao Tome" or Senegal or Serbia or "Sierra Leone" or Sri Lanka or Ceylon or "Solomon Islands" or Somalia or Sudan or Suriname or Surinam or Swaziland or Syria or Principe or Tajikistan or Tadzhikistan or Tadjikistan or Tadzhik or Tanzania or Thailand or Timor-Leste or Togo or "Togolese Republic" or Tonga or Tunisia or Turkey or Turkmenistan or Turkmen or Tuvalu or Uganda or Ukraine or Uzbekistan or Uzbek or Vanuatu or "New Hebrides" or Vietnam or "Viet Nam" or "West Bank" or

For peer review only

Yemen or Zambia or Zimbabwe or Rhodesia or developing countr* or less developed countr* or under developed countr* or underdeveloped countr* or middle income countr* or low income countr* or deprived countr* or underserved countr* or under served countr* or poor countr* or developing nation* or less developed nation* or under developed nation* or underdeveloped nation* or middle income nation* or low income nation* or deprived nation* or underserved nation* or under served nation* or poor nation* or LMIC or lmics or third world* or lami countr* or transitional countr* or "global health"))

For peer review only

## Preferred Reporting Items for Systematic reviews and Meta-Analyses extension for Scoping Reviews (PRISMA-ScR) Checklist

| SECTION | ITEM | PRISMA-ScR CHECKLIST ITEM | REPORTED ON PAGE # |
|---|---|---|---|
| **TITLE** | | | |
| Title | 1 | Identify the report as a scoping review. | 3 |
| **ABSTRACT** | | | |
| Structured summary | 2 | Provide a structured summary that includes (as applicable): background, objectives, eligibility criteria, sources of evidence, charting methods, results, and conclusions that relate to the review questions and objectives. | 1 |
| **INTRODUCTION** | | | |
| Rationale | 3 | Describe the rationale for the review in the context of what is already known. Explain why the review questions/objectives lend themselves to a scoping review approach. | 3 |
| Objectives | 4 | Provide an explicit statement of the questions and objectives being addressed with reference to their key elements (e.g., population or participants, concepts, and context) or other relevant key elements used to conceptualize the review questions and/or objectives. | 4 |
| **METHODS** | | | |
| Protocol and registration | 5 | Indicate whether a review protocol exists; state if and where it can be accessed (e.g., a Web address); and if available, provide registration information, including the registration number. | 4 |
| Eligibility criteria | 6 | Specify characteristics of the sources of evidence used as eligibility criteria (e.g., years considered, language, and publication status), and provide a rationale. | 4-5 |
| Information sources* | 7 | Describe all information sources in the search (e.g., databases with dates of coverage and contact with authors to identify additional sources), as well as the date the most recent search was executed. | 4-5 |
| Search | 8 | Present the full electronic search strategy for at least 1 database, including any limits used, such that it could be repeated. | 25-28 |
| Selection of sources of evidence† | 9 | State the process for selecting sources of evidence (i.e., screening and eligibility) included in the scoping review. | 4-6 |
| Data charting process‡ | 10 | Describe the methods of charting data from the included sources of evidence (e.g., calibrated forms or forms that have been tested by the team before their use, and whether data charting was done independently or in duplicate) and any processes for obtaining and confirming data from investigators. | 4-6 |
| Data items | 11 | List and define all variables for which data were sought and any assumptions and simplifications made. | 5 |
| Critical appraisal of individual sources of evidence§ | 12 | If done, provide a rationale for conducting a critical appraisal of included sources of evidence; describe the methods used and how this information was used in any data synthesis (if appropriate). | NA |


For peer review only - http://bmjopen.bmj.com/site/about/guidelines.xhtml

| SECTION | ITEM | PRISMA-ScR CHECKLIST ITEM | REPORTED ON PAGE # |
|---|---|---|---|
| Synthesis of results | 13 | Describe the methods of handling and summarizing the data that were charted. | 5 |
| **RESULTS** | | | |
| Selection of sources of evidence | 14 | Give numbers of sources of evidence screened, assessed for eligibility, and included in the review, with reasons for exclusions at each stage, ideally using a flow diagram. | 5-6 |
| Characteristics of sources of evidence | 15 | For each source of evidence, present characteristics for which data were charted and provide the citations. | 20-24 |
| Critical appraisal within sources of evidence | 16 | If done, present data on critical appraisal of included sources of evidence (see item 12). | NA |
| Results of individual sources of evidence | 17 | For each included source of evidence, present the relevant data that were charted that relate to the review questions and objectives. | 7-12, 17-24 |
| Synthesis of results | 18 | Summarize and/or present the charting results as they relate to the review questions and objectives. | 7-12 |
| **DISCUSSION** | | | |
| Summary of evidence | 19 | Summarize the main results (including an overview of concepts, themes, and types of evidence available), link to the review questions and objectives, and consider the relevance to key groups. | 12-13 |
| Limitations | 20 | Discuss the limitations of the scoping review process. | 13 |
| Conclusions | 21 | Provide a general interpretation of the results with respect to the review questions and objectives, as well as potential implications and/or next steps. | 13 |
| **FUNDING** | | | |
| Funding | 22 | Describe sources of funding for the included sources of evidence, as well as sources of funding for the scoping review. Describe the role of the funders of the scoping review. | 13 |

JBI = Joanna Briggs Institute; PRISMA-ScR = Preferred Reporting Items for Systematic reviews and Meta-Analyses extension for Scoping Reviews.

* Where *sources of evidence* (see second footnote) are compiled from, such as bibliographic databases, social media platforms, and Web sites.

† A more inclusive/heterogeneous term used to account for the different types of evidence or data sources (e.g., quantitative and/or qualitative research, expert opinion, and policy documents) that may be eligible in a scoping review as opposed to only studies. This is not to be confused with *information sources* (see first footnote).

‡ The frameworks by Arksey and O'Malley (6) and Levac and colleagues (7) and the JBI guidance (4, 5) refer to the process of data extraction in a scoping review as data charting.

§ The process of systematically examining research evidence to assess its validity, results, and relevance before using it to inform a decision. This term is used for items 12 and 19 instead of "risk of bias" (which is more applicable to systematic reviews of interventions) to include and acknowledge the various sources of evidence that may be used in a scoping review (e.g., quantitative and/or qualitative research, expert opinion, and policy document).

*From:* Tricco AC, Lillie E, Zarin W, O'Brien KK, Colquhoun H, Levac D, et al. PRISMA Extension for Scoping Reviews (PRISMAScR): Checklist and Explanation. Ann Intern Med. 2018;169:467–473. doi: 10.7326/M18-0850.


For peer review only - http://bmjopen.bmj.com/site/about/guidelines.xhtml

BMJ Open

# The use of mHealth tools to register birth outcomes in low- and middle-income countries: a scoping review



| | |
|---|---|
| Journal: | *BMJ Open* |
| Manuscript ID | bmjopen-2022-063886.R2 |
| Article Type: | Original research |
| Date Submitted by the Author: | 28-Sep-2022 |
| Complete List of Authors: | Cansdale, Lottie; University of Oxford Medical Sciences Division, Kelly, Gabriella; University of Oxford Medical Sciences Division Khashan, Ali; University College Cork School of Public Health; INFANT Research Centre, University College Cork, Ireland Malata, Address; Malawi University of Science and Technology Kachale, Fannie; Malawi Ministry of Health Lissauer, David ; Malawi-Liverpool-Wellcome Trust Clinical Research Programme; University of Liverpool, Institute of Life Course and Medical Sciences Yosefe, Simeon; Malawi Ministry of Health, Central Monitoring and Evaluation Division Roberts, James; Magee-Women's Research Institute Woodworth, Simon; University College Cork Business School; INFANT Research Centre, University College Cork, Ireland Mmbaga, Blandina; Kilimanjaro Christian Medical University College, Kilimanjaro Clinical Research Institute Redman, Christopher ; University of Oxford, Nuffield Department of Women’s & Reproductive Health Hirst, Jane Elizabeth; University of Oxford, Nuffield Department of Women's and Reproductive Health |
| <b>Primary Subject Heading</b>: | Global health |
| Secondary Subject Heading: | Obstetrics and gynaecology, Public health |
| Keywords: | OBSTETRICS, Information technology < BIOTECHNOLOGY & BIOINFORMATICS, Health informatics < BIOTECHNOLOGY & BIOINFORMATICS |
| | |



SCHOLARONE™
Manuscripts

*I, the Submitting Author has the right to grant and does grant on behalf of all authors of the Work (as defined in the below author licence), an exclusive licence and/or a non-exclusive licence for contributions from authors who are: i) UK Crown employees; ii) where BMJ has agreed a CC-BY licence shall apply, and/or iii) in accordance with the terms applicable for US Federal Government officers or employees acting as part of their official duties; on a worldwide, perpetual, irrevocable, royalty-free basis to BMJ Publishing Group Ltd ("BMJ") its licensees and where the relevant Journal is co-owned by BMJ to the co-owners of the Journal, to publish the Work in this journal and any other BMJ products and to exploit all rights, as set out in our licence.*

*The Submitting Author accepts and understands that any supply made under these terms is made by BMJ to the Submitting Author unless you are acting as an employee on behalf of your employer or a postgraduate student of an affiliated institution which is paying any applicable article publishing charge ("APC") for Open Access articles. Where the Submitting Author wishes to make the Work available on an Open Access basis (and intends to pay the relevant APC), the terms of reuse of such Open Access shall be governed by a Creative Commons licence – details of these licences and which Creative Commons licence will apply to this Work are set out in our licence referred to above.*

*Other than as permitted in any relevant BMJ Author's Self Archiving Policies, I confirm this Work has not been accepted for publication elsewhere, is not being considered for publication elsewhere and does not duplicate material already published. I confirm all authors consent to publication of this Work and authorise the granting of this licence.*

For peer review only

# The use of mHealth tools to register birth outcomes in low- and middle-income countries: a scoping review

LG Cansdale[1], G Kelly[1], AS Khashan[2,3], A Malata[4], F Kachale[5], D Lissauer[6,7], S Yosefe[5], JM Roberts[8], S Woodworth[3,9], BT Mmbaga[10], C Redman[11], JE Hirst[11]

1. University of Oxford Medical School, UK
2. School of Public Health, University College Cork, Ireland
3. INFANT Research Centre, University College Cork, Ireland
4. Malawi University of Science and Technology, Malawi
5. Ministry of Health, Malawi
6. Malawi-Liverpool Wellcome Research Programme, Blantyre, Malawi
7. Institute of Life Course and Medical Sciences, University of Liverpool, UK
8. Magee Women's Research Institute, Department of Obstetrics and Gynecology, Epidemiology and Clinical and Translational Research, University of Pittsburgh, USA
9. Cork University Business School, University College Cork, Ireland
10. Kilimanjaro Clinical Research Institute, Kilimanjaro Christian Medical Centre and Kilimanjaro Christian Medical University College, Moshi, Tanzania
11. Nuffield Department of Women's & Reproductive Health, University of Oxford, UK

Correspondence to:
LG Cansdale, University of Oxford Medical School, UK
Email: lottie.cansdale@st-hildas.ox.ac.uk

## Abstract

**Objective:** Accurate reporting of birth outcomes in low- and middle-income countries (LMICs) is essential. Mobile health (mHealth) tools have been proposed as a replacement for conventional paper-based registers. mHealth could provide timely data for individual facilities and health departments, as well as capture deliveries outside facilities. This scoping review evaluates which mHealth tools have been reported to birth outcomes in the delivering room in LMICs and documents their reported advantages and drawbacks.

**Design:** A scoping review following PRISMA and JBI guidelines for scoping reviews and the mERA checklist for evaluating mHealth interventions.

**Data sources:** PubMed, CINAHL and Global Health were searched for records until 03/02/2022 with no earliest date limit.

**Eligibility criteria:** Studies were included where health workers used mHealth tools in LMICs to record birth outcomes. Exclusion criteria included mHealth not being used at the point of delivery, non-peer reviewed literature and studies not written in English.

**Data extraction and synthesis:** Two independent reviewers screened studies and extracted data. Common themes among studies were identified.

**Results**: 640 records were screened, 21 of which met the inclusion criteria, describing 15 different mHealth tools. We identified six themes: (1) digital tools for labour monitoring (8 studies); (2) digital data collection of specific birth outcomes (3 studies); (3) digital technologies used in community settings (6 studies); (4) attitudes of health workers (10 studies); (5) paper vs electronic data collection (3 studies); and (6) infrastructure, interoperability, and sustainability (8 studies).

**Conclusion**: Several mHealth technologies are reported to have the capability to record birth outcomes at delivery, but none were identified that were designed solely for that purpose. Use of digital delivery registers appears feasible and acceptable to health workers, but definitive evaluations are lacking. Further assessment of the sustainability of technologies and their ability to integrate with existing health information systems is needed.


## Strengths and limitations of this study

- We have used a comprehensive search strategy to explore the literature regarding mHealth and digital birth registers in LMICs.
- We have reported data items using the mERA checklist, which was developed by the WHO to improve reporting of mHealth technologies.

For peer review only

- Only peer-reviewed articles have been included, meaning relevant data in the grey literature or technologies not reported in peer-reviewed articles may have been omitted.

For peer review only



For peer review only - http://bmjopen.bmj.com/site/about/guidelines.xhtml

## INTRODUCTION

Documentation of birth and perinatal events in low- and middle-income countries (LMICs) and obtaining accurate statistics relating to mortality and morbidity can be challenging. Accurate data are needed to monitor health outcomes, to identify problems and to track the impact of interventions, as well as to understand the effects of health system shocks such as COVID-19. Whilst electronic medical records can enable rapid monitoring of trends within units, regions and countries, such hospital-based electronic record systems are expensive, requiring large investments in computer hardware, networks and ongoing technical and security maintenance and support, all of which can be barriers for implementation in LMICs.

Traditionally, births in LMICs are recorded in paper-based delivery registers. These are usually large books, completed by birth attendants soon after the birth. Whilst their content and quality can be variable, they are often the only source of data for collation of perinatal health statistics. Paper-based registers have a short memory and are difficult to store due to their large size. The information in delivery registers is difficult to access when it is needed for patient care at a later date, for comparing data between institutions, or for monitoring trends in care and outcomes over time. Furthermore, non-institutional births may not be recorded at all. LMICs have a high proportion of non-facility births; for example, only 50% of births in Ethiopia, 59% in Bangladesh and 70% in Guatemala take place in healthcare facilities[1]. LMICs are also the countries with the highest maternal and neonatal mortality rates; maternal deaths per 100,000 are estimated at 375 in Uganda, 342 in Kenya and 177 in Indonesia[2]. In other words, those most at risk and in need are those least likely to be documented.

The use of mobile phones is increasing rapidly. One report suggested ownership rates of 80% in 'emerging economies', of which 47% are smartphones[3]. Given the increasing ubiquity of smartphones, their use could improve reporting of delivery and birth outcomes. This application is termed mHealth, and is defined by the WHO as 'the use of mobile wireless technologies for health'[4]. mHealth is already being used in the field of maternal health, with one systematic review identifying 19 different studies describing 15 mHealth interventions that addressed maternal health[5]. This review found mHealth being used for a variety of purposes, including appointment reminders, health promotion, provider-to-provider communication and data collection. However, at



For peer review only - http://bmjopen.bmj.com/site/about/guidelines.xhtml

present it is unclear to what extent mHealth is used to capture birth data at the point of delivery, whether this approach is acceptable to health workers, and whether it improves recording of perinatal outcomes. Hence, the purpose of this scoping review is to understand what mHealth technologies have been evaluated to record birth outcomes at the time and place of delivery in LMICs.

## METHODS

This review was developed in line with the PRISMA and JBI (Joanna Briggs Institute) guidelines for scoping reviews[6, 7], using the PCC mnemonic – population, concept, context. A scoping review gives an overview of the evidence, maps the literature and identifies and analyses current gaps in knowledge. A review protocol was not published for this study.

For peer review only

### *Population, concept, context*

*Population*

The population of this review comprises healthcare workers recording delivery outcomes, namely midwives, community health workers, doctors and other cadres of skilled birth attendants in LMICs. LMICs were defined according to the World Bank categorisation of low, lower-middle and upper -middle in 2021[8].

*Concept*

Our focus is the use of mHealth to capture data at birth, and specifically those technologies present at the place of delivery. Of these many are expected to have wider applications, such as recording of the outcomes of antenatal and postnatal appointments, or progress in labour via an e-partogram. For this scoping review, we have included any technology if outcomes at birth were recorded by the birth attendant or community health worker, at the time of, or shortly after, delivery.

*Context*

The context of the review are the places where births occur in LMICs. This includes home and community deliveries, as well as institutional births in health centres, nursing homes and government or private hospitals.

*Search strategy*

To identify relevant sources, three databases were searched: PubMed, CINAHL and GLOBAL HEALTH. The search strategy was created with an experienced librarian and further refined by the authors, including key search terms such as 'childbirth', 'mobile applications' and 'midwives'. The final search strategies for each database can be found in the appendix. The databases were searched for relevant articles up to 03/02/2022, with a lower date limit not specified as the term 'mHealth' was not widely used prior to 2000. The final searches were uploaded into EndNote and duplicates were removed by their duplicate detection software.

*Screening and selection of relevant studies.*

Once the citations had been extracted to EndNote, they were uploaded on to Colandr (colandrapp.com), a software that manages citation and full-text screening for reviews. Texts were first screened by abstract, and those that met the inclusion criteria then went on to the full-text screen. Two reviewers (LC and GK) did both citation and full-text screening of articles, and any disputes between reviewers were resolved by a third author (JH).

References of studies included for full-text screening were hand-searched to identify for further relevant studies. These hand-picked citations were uploaded to EndNote and then included in citation screening. Once imported into EndNote duplicates were removed.

*Inclusion and exclusion criteria*

Eligible studies were conducted in LMICs (defined by the World Bank list of LMICs)[8]. Within each study, the mHealth tool needed to be capable of collecting data regarding birth outcomes at the place of delivery. We considered studies where the data was collected by nurses/midwives/birth attendants. We limited the search to English language publications only.

We excluded studies where no birth outcomes were reported in the publication, where data were collected using hospital Electronic Health Record (EHR) systems on desktop computers, where birth registration data were collected after the delivery or for the purpose of vital registration only, and where data were collected by people

For peer review only


For peer review only - http://bmjopen.bmj.com/site/about/guidelines.xhtml

other than nurses/midwives/birth attendants. As this was intended as a scoping review of published literature, we excluded non-peer-reviewed reports of technologies, such as reports, web pages and media releases.

### *Data extraction and synthesis*

An Excel spreadsheet was used for data-extraction, with variables to extract agreed through discussion between the authors. The two reviewers (LC and GK) independently charted data and discussed results.

We abstracted data on article characteristics, including country of origin, funder, form of mHealth, technology user, reported birth outcomes and technology feasibility and acceptability.

We conducted a narrative synthesis based on our findings. We first mapped studies by geographic location, technology type, primary intended use of technology, accessibility outside the program described (i.e., whether the technology was Open Access or not), and intended user group (hospital/clinic staff or in the community). We then identified the main thematic areas that have been evaluated to date, with the purpose of summarising the current state of the academic literature in this field and identifying gaps for further research. We made particular use of mERA (mHealth Evidence Reporting and Assessment), a checklist which was developed by the WHO to standardise mHealth evidence reporting[9]. Given this was a scoping review to map the quantity and domains of evidence on digital birth registers, the quality of studies was not assessed.

### *Patient and public involvement*

None.

## RESULTS

### *Identification of eligible studies for review*

The search strategy is outlined in Figure 1. The initial search was carried out on 31/07/2021 and updated on 03/02/2022, yielding 567 and 640 records respectively. Of the records identified, 578 were from PubMed, 34 from CINAHL, 28 from Global Health.

For peer review only

*Study characteristics and themes*

The characteristics of each study are described in Table 1, together with the six specific themes we identified. The themes are elaborated in more detail in sections 3-6 below. Table 2 describes the technical aspects and content of interventions.

**Table 1: Study characteristics and themes**

| **Technology**<br>Author/s, year/s | **Country** | **Study design[1], N participants** | **Labour[a]** | **Outcomes[b]** | **Comm[c]** | **Attitudes[d]** | **Paper vs elect[e]** | **Sustain[f]** |
|---|---|---|---|---|---|---|---|---|
| **1. ePartogram** | | | | | | | | |
| Litwin et al, 2018[10] | Tanzania | Feasibility and acceptability<br>23 skilled birth attendants | 🟢 | 🔴 | 🔴 | 🟢 | 🟡 | 🟡 |
| Sanghvi et al, 2019[11] | Kenya | Mixed method, quasi-experimental<br>110 skilled birth attendants | 🟢 | 🔴 | 🔴 | 🟢 | 🟢 | 🔴 |
| **2. E-partograph** | | | | | | | | |
| Rahman et al, 2019[12] | Bangladesh | Prospective cross-over study<br>Nurse-midwives (NNS*) | 🟢 | 🔴 | 🔴 | 🟢 | 🟡 | 🟡 |
| Tadesse et al, 2019[13] | Ethiopia | Cross-sectional study with questionnaire<br>462 obstetric care providers | 🟢 | 🔴 | 🔴 | 🟢 | 🟡 | 🟡 |
| **3. mLabour** | | | | | | | | |
| Schweers et al, 2016[14] | India | Design and preliminary evaluation<br>12 nurses | 🟢 | 🔴 | 🔴 | 🟢 | 🟡 | 🔴 |
| **4. prasavGraph** | | | | | | | | |
| Singh et al, 2016[15] | India | Development and pilot<br>Doctors and student nurses (NNS) | 🟢 | 🔴 | 🔴 | 🔴 | 🔴 | 🟡 |
| **5. ASMAN** | | | | | | | | |
| Usmanova et al 2020[16] | India | Qualitative study<br>44 stakeholders | 🟢 | 🔴 | 🟡 | 🟢 | 🟡 | 🟢 |
| Usmanova et al 2021[17] | India | Secondary analysis of programme data | 🟢 | 🔴 | 🔴 | 🔴 | 🔴 | 🔴 |

For peer review only

| | | | | | | | | |
|---|---|---|---|---|---|---|---|---|
| | | (NNS) | | | | | | |
| **6. PPH reporting** | | | | | | | | |
| Andreatta et al, 2011[18] | Ghana | Evaluation of technology<br>10 birth attendants | 🔴 | 🟢 | 🟢 | 🔴 | 🔴 | 🔴 |
| **7. Mobile Delivery Timer** | | | | | | | | |
| Somannavar et al 2015[19] | India | Development and evaluation<br>46 birth attendants | 🔴 | 🟢 | 🔴 | 🟢 | 🔴 | 🟡 |
| **8. Birth defects surveillance system** | | | | | | | | |
| Mumpe-Mwanja et al, 2019[20] | Uganda | Surveillance study<br>Midwives (NNS) | 🔴 | 🟢 | 🔴 | 🔴 | 🔴 | 🔴 |
| **9. RapidSMS-MCH** | | | | | | | | |
| Ngabo et al, 2012[21] | Rwanda | Design and implementation<br>432 community health workers | 🔴 | 🔴 | 🟢 | 🔴 | 🔴 | 🟢 |
| Musabyimana et al, 2018[22] | Rwanda | Qualitative study<br>93 participants including community health workers | 🔴 | 🔴 | 🟢 | 🟢 | 🔴 | 🟢 |
| **10. Data collection** | | | | | | | | |
| Little et al, 2013[23] | Ethiopia | Development and evaluation<br>12 midwives, 20 health extension workers | 🔴 | 🔴 | 🟢 | 🟢 | 🔴 | 🟢 |
| Medhanyie et al, 2015[24] | Ethiopia | Semi-structured questionnaire<br>23 midwives and health extension workers | 🔴 | 🔴 | 🟢 | 🟢 | 🟢 | 🟢 |
| Medhanyie et al, 2017[25] | Ethiopia | Comparative cross-sectional study<br>25 midwives and health extension workers | 🔴 | 🔴 | 🟢 | 🔴 | 🟢 | 🔴 |
| **11. Perinatal Monitoring App** | | | | | | | | |
| Stroux et al 2016[26] | Guatemala | Prototype testing<br>9 healthcare workers | 🔴 | 🔴 | 🟢 | 🟡 | 🔴 | 🟢 |
| Martinez et al, 2017[27] | Guatemala | Agile development<br>25 midwives | 🔴 | 🔴 | 🟢 | 🟡 | 🔴 | 🟢 |

For peer review only

| | | | | | | | | |
|---|---|---|---|---|---|---|---|---|
| Martinez et al, 2018[28] | Guatemala | Randomised control trial<br>44 traditional birth attendants | ● (red) | ● (red) | ● (green) | ● (red) | ● (red) | ● (red) |
| **12. Hayat** | | | | | | | | |
| Zaidi et al, 2020[29] | Pakistan and Afghanistan | Qualitative exploratory study<br>54 lady health workers, 42 community health workers | ● (red) | ● (red) | ● (green) | ● (green) | ● (yellow) | ● (green) |
| **13. Maternal referral mobile system** | | | | | | | | |
| Indriani et al, 2020[30] | Indonesia | Development of technology<br>Midwives<br>(NNS) | ● (red) | ● (red) | ● (green) | ● (yellow) | ● (yellow) | ● (yellow) |

Key:

● (red) = not described ● (green) = described ● (yellow) = partially described

[1]Study design taken from description in paper

[a]Digital tools for labour monitoring
[b]Digital collection for specific birth outcomes
[c]Digital tools for use in the community
[d]Attitudes of health workers towards digital collection of birth outcomes
[e]Comparison of paper and electronic data tools
[f]Infrastructure, interoperability and sustainability
NNS= number not stated.

**Table 2: Technical aspect and intervention content**

| Name | Platform | Interoperability/ HIS context | Intervention content |
|---|---|---|---|
| **1. ePartogram** | Android tablet App | Data accessible at referral facilities | Mobile partograph with automatic graphing of data, clinical decision support and auditory alerts |
| **2. E-partograph** | Android Smartphone or tablet App | Partograph data monitored remotely | Mobile partograph with automatic graphing of labour progress and alerts for abnormalities |
| **3. mLabour** | Mobile App | NS | Mobile partograph and labour management tool to register patients, record measurements and record delivery |

For peer review only

| | | | |
|---|---|---|---|
| **4. prasavGraph** | Android Smartphone or tablet app | Birth registration with municipal authorities | Mobile partograph with digital record keeping and notifications for abnormalities |
| **5. ASMAN** | Tablet based | Integrated into government databases | Platform with digital case sheets, dashboards, E-learning content and remote support centre |
| **6. PPH Monitoring** | SMS based | NS | Numeric SMS protocol to report maternal demographics and birth outcomes |
| **7. Mobile Delivery Timer** | Android smartphone App | NS | Application with voice recording to verbally mark crowning, birth, crying or bag mask ventilation of baby |
| **8. Birth defect surveillance** | Android tablet App | NS | Application to document birth defects and record birth outcomes |
| **9. RapidSMS** | Open-source App | Automatic notification to ambulance service | SMS protocols to record pregnancy, maternal and child outcomes, allows communication between CHWs and health facilities |
| **10. Data collection tool** | Smartphone App | Local health authority | Application with maternal health care forms and analytics dashboard |
| **11. Perinatal Monitoring System** | Android smartphone App | Open MRS | Tools to record maternal vital signs, clinical decision support tool, supports communication between birth attendants and medical team |
| **12. Hayat** | Smartphone App | Local health authority | Mobile application to digitalise data entry, and dashboard to visualise health reports |
| **13. Maternal referral Mobile System** | Smartphone App | Data available at referral centre | Application to communicate maternal referrals between primary health care and referral hospital |

HIS= Health information system. NS= Not stated. CHW= Community health worker.

For peer review only

We identified 15 different mHealth technologies, across 12 different countries: 7 in Africa, 7 in Asia and 1 in South America. A variety of health workers were end-users of the technologies, including midwives, nurses, traditional and skilled birth attendants (TBAs and SBAs), and community health workers (CHWs).

Six technologies were described in more than one paper. The ePartogram and E-partograph were studied in two different settings: Tanzania and Kenya for the ePartogram[10, 11], and Bangladesh and Ethiopia for the E-partograph[12, 13]. The further studies on the other four technologies (RapidSMS, the data collection tool, perinatal monitoring app and ASMAN) were conducted in the same setting as the first published paper.

A variety of methods were used to assess the technologies, with the majority being early phase development and pilot work. Only one study reported results of a randomised control trial[28], whilst three studies were preclinical[15, 26, 30]. Sample sizes also varied between studies: the largest was 462[13] and smallest 9[26]. Five studies did not document the number of health workers using the technology[12, 15, 17, 20, 30].

### *Roles for the digital tools*

The six themes identified were split into roles for technologies and focus of studies. The three main roles for digital tools at birth were: (1) labour monitoring; (2) recording of a specific birth outcome; (3) tools for use in the community, and studies were mainly concerned with: (1) attitudes of health workers to digital technologies; (2) comparison of paper and digital data collection; and (3) sustainability, interoperability, and infrastructure for mHealth interventions.

### *Digital tools for labour monitoring*

Eight of the studies described tools that could monitor labour, all based on the WHO partograph[10-17] and shown in Table 3. Whilst the primary purpose of the partograph is to monitor labour these technologies had additional capabilities to record birth data, often to assess the impact of labour monitoring on birth outcomes.

**Table 3: Digital tools for labour monitoring**

For peer review only

| Technology | Studies |
|---|---|
| ePartogram | [10, 11] |
| E-Partograph | [12, 13] |
| mLabour | [14] |
| prasavGraph | [15] |
| ASMAN | [16, 17] |

Several electronic partographs have been described. The ePartogram was developed by John Hopkins University and their NGO arm, Jhpiego. Study of its use in Tanzania showed SBAs were comfortable registering clients and entering data into the application and had a preference for the ePartogram over paper versions[10]. This was replicated in a study in Kenya, where it was also found to improve birth outcomes compared to paper partograph use[11]. Improvements in birth outcomes was also seen in studies investigating the E-Partograph in Bangladesh[12]. However, a separate study of the E-partograph in Ethiopia found only 46% of care providers would be willing to use a mobile-based E-partograph, despite 65.8% of caregivers reporting they wished to use a partograph routinely[13].

Three other technologies were identified that had a digital partograph as part of their workflow. prasavGraph is an app incorporating a partograph with other data collection, including the baby's sex, weight and condition at birth, reported in preclinical phase in New Delhi[15]. mLabour also incorporated a partograph into their labour management tool; nurses using it reported being more punctual with data entry and found the tablet form lighter and easier to record data in compared to multiple paper registers[14]. This feedback was echoed with the ASMAN application, which consists of a digital case sheet for patients, partograph, dashboard for health facilities and E-learning content, and was used in both district hospitals and community health centres[16]. Analysis of application data showed high rates of data collection for delivery in the app, at 93.7%[17].

*Digital collection for specific birth outcomes*

Three technologies were designed to report a specific outcome, but also recorded delivery details as part of the workflow. A system for community midwives to report postpartum haemorrhage (PPH) was designed in Rwanda,

For peer review only

using mobile phone handsets and SMS protocols[18]. As well as PPH, data were collected on maternal and neonatal deaths. Ten TBAs used the technology and successfully reported 425 births and 13 incidents of PPH.

The other two studies collected data collected on tablets. The Mobile Delivery Timer was designed to measure the time between delivery and either the baby crying or initiation of bag mask ventilation, as part of a larger 'Helping Babies' Breath' study[19]. This recorded the time of delivery as well as the woman's ID and status of the newborn. 90% of birth attendants found recording data in the mobile phone either 'moderately easy to use' or 'very easy to use'. Android tablets were used in a tool to detect birth defect prevalence in Uganda[20]. All births were examined by a midwife as soon as feasible and data collected on forms designed using Open Data Kit (ODK), including both pictures of any major defects as well as quantitative data documenting maternal age, birth outcome, newborn sex and mode of delivery. The system was able to detect a birth defect prevalence of 66 per 10,000 births, but the authors noted there was no population-based data for comparison.

***Digital tools for use in the community***

Six of the tools were designed specifically to be used in the community, either by trained midwives or lay or volunteer health workers.

Two technologies used mobile phones and SMS protocols to capture community data. The PPH reporting system is one example[18], as is the RapidSMS-MCH system in Rwanda[21]. In Rwanda, volunteer community health workers were given mobile phones with eight SMS forms for reporting data, including one for delivery. The SMS was sent to a central server and could alert an ambulance in case of emergency. The technology was scaled up nationwide in 2013, and a further study found it was well accepted by both health workers and the local community[22].

A further three technologies used smartphones to record data collected by community health workers. An app was designed for data collection by midwives and health extension workers (HEWs) in Ethiopia, containing eight data collection forms for different perinatal events[23]. It also contained a scorecard and analytics dashboard to allow data monitoring by local health bureaus. The app was adapted for use in the community by using the local calendar


For peer review only - http://bmjopen.bmj.com/site/about/guidelines.xhtml

and local language. Analysis of the records submitted found that most delivery protocols were submitted by midwives rather than HEWs, reflecting local guidance that HEWs should not assist with deliveries[24]. A similar format was seen with the Hayat app in Afghanistan and Pakistan; it was designed to digitalise data entry, and also contained a dashboard to visualise reports generated by patient encounters[29]. Community health workers were able to successfully use the app but highlighted issues such as the app not using the local language. The perinatal monitoring tool used in Guatemala was adapted for community use by having audio and visual instructions and data collection through audio recordings, to be inclusive of illiterate and technology-naïve end-users[26, 27]. Similarly, to the other technologies, it collects data based on antenatal, postnatal and intrapartum encounters, combined with recording of vital signs. Once assessment is complete, the data is uploaded to the patient's OpenMRS electronic medical record. An initial feasibility study showed use was feasible among traditional birth attendants, and later randomised control testing showed that use of the app was associated with improved detection of pregnancy complications and a higher rate of referrals to medical facilities[28].

Several of the tools were designed to aid communication between health professionals. A mobile referral system was designed in Indonesia, allowing midwives to relay information about patient condition and neonatal outcomes to the referral hospital[30]. This was a feature of other platforms: the perinatal monitoring system alerted the local health facility if a complication was detected[28], and RapidSMS could automatically forward ambulance requests in an emergency[21]. ASMAN also facilitated communication, through access to a remote support centre staffed by health personnel[16].

*Attitudes of health workers towards digital collection of birth outcomes*

Ten studies explored the attitudes of health workers towards digital tools. Seven used focus groups or in-depth interviews[10, 11, 14, 16, 22, 23, 29] and three used questionnaires[13, 19, 24]. All but one of the studies reported that the technology was well received and preferred to conventional methods of data collection.

Four studies were specifically designed to explore the preferences of health workers[13, 16, 22, 24]. Generally they showed positive attitudes towards digital data collection; one study in Ethiopia found 87% of health workers perceived it to be useful[24], and ownership of the mobile phones was a motivating factor for using the technology

For peer review only

for 91.3%. The RapidSMS project also gave mobiles to health workers, and whilst the technology was perceived to improve birth outcomes, the increased reporting requirement reduced the motivation of the volunteer CHWs, leaving some wanting financial compensation for the increased workload[22]. Only one study used a validated tool to study health worker preferences; the Technology Acceptance Model, which explores perceived ease of use and usefulness of a technology, was used to evaluate the ASMAN application[16]. The application was reported to be easy to use and less error prone compared to paper, and improved clinical documentation by prompting users to ask for additional information as necessary.

Only one study showed a negative attitude towards a digital technology[13]. Analysis of attitudes towards the E-Partograph showed that whilst 99.6% of workers owned a mobile phone, only 46% were willing to use a mobile-based E-Partograph; however, this did not specify whether they were unwilling to use the app on their personal phone or unwilling to use the E-Partograph in general. 58.9% of those surveyed had a favourable attitude towards the partograph, and this was associated with willingness to use the E-Partograph, so the authors concluded that the results may be due to negative attitudes towards the partograph in general rather than an electronic tool specifically.

In six studies the time taken to use digital technologies was mentioned by health workers. In three, digital data collection was perceived to be a timesaver[10, 16, 29]. However, those using the ePartogram were concerned about achieving timely data entry in high volume sites or with multiple clients[11], and 78.3% of those using the Ethiopian data collection tool cited time taken as a reason for not using forms all the time[24]. With ASMAN, whilst facilities using the application alone to collect data found it reduced reporting time, some facilities required data collection electronically and on paper for verification, audit or in case of paper issues, which increased workload temporarily[16].

*Comparisons of paper and electronic tools*

mHealth tools were formally compared to the current standard of reporting, paper records, in three of the studies[11, 12, 25]. This was either comparing preferences for paper or electronic data collection or comparing completeness of records.

For peer review only


For peer review only - http://bmjopen.bmj.com/site/about/guidelines.xhtml

The current WHO partograph is a paper version, so studies using an electronic version often used this as a comparison. One cross-over study comparing the E-partograph to the paper version found higher user rates during the electronic phase in both sites[12]. Another study found that all measurements except foetal heart rate and contractions were recorded more frequently in the ePartogram compared to paper[11]. However, SBAs found that it was more difficult to correct errors in data entry using the ePartogram, which was not an issue with the paper charts.

Similar findings were seen with the data collection tool in Ethiopia[25]. Comparing the completeness and accuracy of patient data collection on electronic forms compared to paper, they found overall completeness was 8% higher in electronic records compared to paper. Whilst there was no difference for parameters such as patient name or age, for newborn weight recording was 20.5% higher in electronic forms. The authors speculated this could be because the electronic forms required a response to be accepted. However, they also found some workers lacked the apparatus to measure some parameters, such as scales for recording newborn weight, and had thus been discouraged from using electronic forms to record data due to mandatory reporting requirements, meaning they were more likely to use paper and reduce the completeness of paper records. The study reported that missing data on paper forms was also due lack of physical space on paper sheets.

***Infrastructure, interoperability and sustainability***

Few studies included information about the infrastructure necessary for mHealth technology. Some described the hardware given to study participants – for example, in Ethiopia workers were given second-hand HTC Hero smartphones, solar lamp and phone chargers[23]. They reported that of the 23 health posts in the area, only 48.9% had GPRS at the time, so only chose health posts with connectivity for the study. Network connectivity varied across the studies, with some reporting 3G being available in all facilities[10], whereas those using the Hayat app cited a lack of connectivity as a barrier to uploading data[29]. The Hayat app had also GPS capabilities, so that health workers could be tracked during outreach visits; however, workers had to travel to the central offices once per week to upload data to the central server, which was also the case with the perinatal monitoring system in Guatemala[26].

For peer review only

Four studies reported capability to communicate with pre-existing health information systems (HIS) or upload data to electronic health/medical records (EHR/EMR)[16, 23, 26, 29]. The perinatal monitoring app in Guatemala has been used in the community and is able to upload data to the OpenMRS EMR once assessment is complete[26]. However, in the randomised control trial of the application, this feature was not mentioned[28]. The data collection project in Ethiopia discussed uploading their data to EMRs but stated that improvements in patient identification would be needed for this to be successful[23]. However, the Ethiopian data collection app, the Hayat app[29], and the ASMAN package[16] all created a dashboard from data collection reports submitted, which allowed local health bureaus to view the data. Beside connecting to a HIS, prasavGraph reported that their data could be used to record births with municipal authorities, but this has not progressed beyond prototype testing[15].

Table 4 contains a summary of user feedback, contextual adaptability and limitations for delivery at scale. Not all the studies considered sustainability of the intervention, or the feasibility of scaling technology up. Scaling-up was discussed with regards to ASMAN, detailing a transition package to move past the pilot phase involving transferring the application to the states' servers and integrating ASMAN data fields into government bases[16]. However at the time of writing, only RapidSMS-MCH had been scaled up, to involve all CHWs across Rwanda[22].

**Table 4: Limitations and contextual adaptability**

| Name | User feedback | Limitations for delivery at scale | Contextual adaptability |
|---|---|---|---|
| **1. ePartogram** | | | |
| | • Improved decision making<br>• Data entry simple and user-friendly | • Concerns about time for data entry in high-volume labour wards | Feedback session with SBAs done as part of development process |
| **2. E-partograph** | | | |
| | • 44% were willing to use<br>• 58.9% had favourable attitudes towards the partograph | • Obstetric care providers less willing to use | NS |

For peer review only

| | | |
|---|---|---|
| **3. mLabour** | | |
| • Users preferred tablet format to paper<br>• Requests for additional functionality, e.g. tracking medications | NS | Some features of app customised for context of specific hospital, not wider use |
| **4. prasavGraph** | | |
| NS | NS | Designed for use in settings with poor network connection |
| **5. ASMAN** | | |
| • Technology easy to use<br>• Perceived improvement in job performance | • Technology and internet issues<br>• High caseloads prevented all fields being completed | E-learning written in English and Hindi with audio, visual and readable formats |
| **6. PPH Monitoring** | | |
| NS | • Poor network and power outages<br>• Lack of remuneration | Training given in English and native language |
| **7. Mobile Delivery Timer** | | |
| • 90% found application easy to use<br>• 70% felt app was beneficial | NS | Android operating system chosen as functions on low-cost hardware |
| **8. Birth defect surveillance** | | |
| NS | • System does not capture babies born outside of urban facilities | NS |
| **9. RapidSMS** | | |
| • Workers reported being more pro-active in finding pregnant women<br>• Requests for financial compensation | • High initial cost<br>• Lack of equipment prevented all data being collected | Mobile phones provided to CHWs to reduce their costs |
| **10. Data collection tool** | | |
| • 52.2% said forms were comprehensive<br>• 87% found electronic forms helpful<br>• Preferred electronic forms to paper | • High cost of covering phone airtime | Provided with solar lamp to charge smartphone |
| **11. Perinatal Monitoring System** | | |
| • System was easy to operate with potential benefit | • Unfamiliarity with technology<br>• Low quality ultrasound recordings | Audio and visual instructions used to adapt user interface for illiterate users |
| **12. Hayat** | | |

For peer review only

| | | |
|---|---|---|
| • App use feasible and improved efficiency<br>• Frustrated at inability to edit entries | • CHWs needed to travel long distances each week to sync app data with central server | NS |
| **13. Maternal referral Mobile System** | | |
| NS | • Bugs in application | Focus group interviews conducted prior to development |

NS= Not stated.

Most of the technologies used Android hardware, which is widely available in LMICs and is relatively low cost; one study cited its use as a way to increase sustainability[19]. The sustainability of the perinatal monitoring system in Guatemala was assessed in relation to its cost which was less than $50 for the equipment, while its software was open source[27]. The US engineers involved in the development of the app also spent time training engineers in Guatemala to be able to deal with software and hardware issues relating to the technology[27]. Issues in scaling up mHealth use were highlighted in the Ethiopian study where workers were given cell phones; they said that the cost of airtime was high owing to workers using phones for personal and study purposes. They suggested that the intervention would be more sustainable if a mechanism could be found either to provide free airtime for uploading protocols or to restrict airtime to study protocols[24].

## DISCUSSION

We identified 21 studies describing 15 mHealth technologies that could collect data in the delivery room. Studies showed that data collection at delivery is both feasible and acceptable to healthcare providers. Of the six themes we identified, three were the primary purposes technologies were designed for: labour monitoring, collection of a specific birth outcome and data collection in the community. Whilst no technology was specifically designed to record birth outcomes in the delivering room, our findings suggest that use of such a tool would be feasible and acceptable.

For peer review only

All but one of the studies found that digital data collection was preferred to paper methods. Digital technologies were reported to be easy to use[16, 19], user-friendly[11] and perceived to improve worker efficiency[29] and birth outcomes[22]. Health workers also preferred the digital format compared to paper methods, noting them to be less bulky to carry and easier to access[14, 29]. It was also reported that digital collection could improve the number of data items reported, by incorporating mandatory data entry fields[16, 22].

We found that the time taken to input data can be a barrier. In some studies, this study design requiring paper and electronic entry rather than issues with the tool itself[16, 24]. However, we also found evidence that health workers were concerned about time to input data[23], particularly in facilities caring for large numbers of patients[11]. Digital tools can mandate reporting of more items, and whilst this is useful for data collection and research, it can be time-consuming for health workers and reduce motivation to record data[22]. An mHealth application that did not meet criteria for inclusion, mClinic, sought end-user feedback on items for data reporting, finding that users only wanted items that would be included in reports and did not want free-text for reporting of subjective data[31]. Clearly, end-user involvement in designing data collection protocols is important to ensure that each data item is perceived to be useful for health worker's practice.

We found that there are several gaps in reporting of interventions in the published literature. One of the items in the mERA checklist, developed by the WHO, is 'Interoperability/Health information systems (HIS) context', which involves 'describing how mHealth intervention can integrate into existing health information systems'[9]. Our review found few studies describing how the mHealth tool would integrate with the existing HIS; only the perinatal monitoring app appeared to upload data directly to patient's medical records app[26]. Another item in the mERA checklist is 'Cost Assessment', and again only the perinatal monitoring app mentioned the cost of the hardware required to run the technology[26]. Consideration of both cost and interoperability are crucial for the sustainability[32], so the lack of reporting in the literature highlights the need for both standardised reporting of interventions and for authors to consider these factors when designing and testing new technology.

Few tools were identified with the capability to record births in both facilities and in the community. Whilst several of the community-based technologies could communicate data to facilities, only the Ethiopian data


For peer review only - http://bmjopen.bmj.com/site/about/guidelines.xhtml

collection tool was used to collect data by both health workers in the community and midwives in health centres[23]. Since the proportion of non-facility births in many LMICs is high[1], a digital birth register would need to be designed for use in both the community and facilities to ensure maximum coverage of births.

To our knowledge, no other review has considered the use of mHealth specifically in the delivering room, or its potential to replace paper-based delivery registers. However, our review is in agreement with other reviews that there is a lack of high-quality evidence and robust study design to determine the efficacy and effectiveness of mHealth interventions in maternal health care[5, 33, 34]. Of the studies we identified, only one was a randomised-control trial, and of the 15 technologies only six were evaluated by more than one study. With regards to the purpose of evaluation, only two studies formally assessed the accuracy of digital data collection compared to paper methods[11, 25], and only six studies explored the time needed for digital input relative to paper recording[10, 11, 16, 24, 29] . The lack of quality evidence creates a barrier to scaling up technologies and emphasises the need for pilot studies to be followed-up with studies that investigate scalability[32].

For peer review only

Our own review has its strengths and limitations. Whilst we used a comprehensive search strategy, we only included peer-reviewed articles, omitting potentially relevant data published in the grey literature. We were also limited to studies published in English. Publication bias was also a limitation – whilst we included all available studies that were captured in our search, there may be other relevant technologies that have not been described in peer-reviewed articles.

In conclusion, this review identified several mHealth technologies capable of recording birth outcomes in the delivering room. Whilst none of the technologies were specifically designed for this purpose, the evidence suggests use of digital delivery registers would be both feasible and acceptable to health workers. Few of the tools identified had adequate reporting of interoperability and sustainability, and more research is needed to determine the efficacy and effectiveness of their use.



For peer review only - http://bmjopen.bmj.com/site/about/guidelines.xhtml

## ACKNOWLEDGEMENTS

We are grateful to librarian N. Thomas for helping build the initial search strategy.


## CONTRIBUTORS

JEH, CR, ASK, AM, DL, FK conceived the review idea. LGC carried out the search and LGC and GK screened and selected articles. LGC wrote the manuscript with input from GK, ASK, AM, FK, DL, SY, JMR, SW, BTM, CR, JEH. All authors approved the manuscript prior to submission.

For peer review only

## DATA AVAILABILITY STATEMENT

All data are included in the manuscript.


## FUNDING

There was no specific funding for this project. JEH is funded by a UKRI Future Leaders Fellowship.


## ETHICS APPROVAL

Ethics approval is not applicable for this article as it is based on existing published work.

## COMPETING INTERESTS

The authors declare that there were no conflicts of interest.

## AUTHOR REFLEXIVITY STATEMENT

The original concept for this systematic review was co-developed equally by the authors from high and low-income country settings to address an identified priority from the authors from Tanzania and Malawi. The protocol was jointly developed and the search strategy and data extraction were performed by HIC researchers, with support from LIC researchers. The LIC researchers contributed to interpretation of themes and ensuring contextual relevance. All authors read and approved the final version of the manuscript. The first (LGC) and last author (JEH) are both HIC authors, as the first author is a student who has been supervised by the senior author.

## *APPENDIX*

The search strategies can be found in the appendix.

## REFERENCES


1. [dataset] UNICEF. Data from: Maternal and Newborn Health Coverage Database. UNICEF May 2022 https://data.unicef.org/topic/maternal-health/antenatal-care/. Date Accessed September 2022

2. [dataset] WHO, UNICEF, UNFPA, World Bank Groupand UNPD. Data from: Trends in estimates of maternal mortality ratio (MMR; maternal deaths per 100,000 live births) maternal deaths and lifetime risk of maternal death, 2000-2017. WHO, Geneva September 2019 https://data.unicef.org/topic/maternal-health/maternal-mortality/. Date Accessed December 2021

3. Smartphone Ownership Is Growing Rapidly Around the World, but Not Always Equally https://www.pewresearch.org/global/2019/02/05/smartphone-ownership-is-growing-rapidly-around-the-world-but-not-always-equally/. Date Accessed February 2022

4. World Health Assembly. mHealth: use of appropriate digital technologies for public health: report by the Director-General. World Health Organization March 2018 https://apps.who.int/iris/handle/10665/276430

5. Colaci D, Chaudhri S, Vasan A. mHealth Interventions in Low-Income Countries to Address Maternal Health: A Systematic Review. *Ann Glob Health* 2016;82(5):922-35. doi: 10.1016/j.aogh.2016.09.001

6. Tricco AC, Lillie E, Zarin W, et al. PRISMA Extension for Scoping Reviews (PRISMA-ScR): Checklist and Explanation. *Ann Intern Med* 2018;169(7):467-73. doi: 10.7326/M18-0850 [published Online First: 4 September 2018]

7. Peters MDJ, Godfrey C, McInerney P et al. Chapter 11: Scoping Reviews. In: Aromataris E, Munn Z eds. JBI Manual for Evidence Synthesis. JBI, 2017

8. [dataset] The World Bank. Data from: Low and middle income. The World Bank. https://data.worldbank.org/income-level/low-and-middle-income. Date Accessed December 2021

9. Agarwal S, LeFevre AE, Lee J, et al. Guidelines for reporting of health interventions using mobile phones: mobile health (mHealth) evidence reporting and assessment (mERA) checklist. *BMJ* 2016;352:i1174. doi: 10.1136/bmj.i1174 [published Online First: 17 March 2016]


For peer review only



For peer review only - http://bmjopen.bmj.com/site/about/guidelines.xhtml

10. Litwin LE, Maly C, Khamis AR, et al. Use of an electronic Partograph: feasibility and acceptability study in Zanzibar, Tanzania. *BMC Pregnancy Childbirth* 2018;18(1):147. doi: 10.1186/s12884-018-1760-y [published Online First: 9 May 2018]

11. Sanghvi H, Mohan D, Litwin L, et al. Effectiveness of an Electronic Partogram: A Mixed-Method, Quasi-Experimental Study Among Skilled Birth Attendants in Kenya. *Glob Health Sci Pract* 2019;7(4):521-39. doi: 10.9745/GHSP-D-19-00195 [published Online First: 23 December 2019]

12. Rahman A, Begum T, Ashraf F, et al. Feasibility and effectiveness of electronic vs. paper partograph on improving birth outcomes: A prospective crossover study design. *PLoS One* 2019;14(10):e0222314. doi: 10.1371/journal.pone.0222314 [published Online First: 7 October 2019]

13. Tadesse Y, Gelagay AA, Tilahun B, et al. Willingness to Use Mobile based e-Partograph and Associated Factors among Care Providers in North Gondar Zone, Northwest Ethiopia. *Online J Public Health Inform* 2019;11(2):e10. doi: 10.5210/ojphi.v11i2.9468 [published Online First: 19 September 2019]

14. Schweers J, Khalid M, Underwood H, et al. mLabour: Design and Evaluation of a Mobile Partograph and Labor Ward Management Application. *Procedia Engineering* 2016;159:35-43. doi: https://doi.org/10.1016/j.proeng.2016.08.061

15. Singh S, Khalid AM, Paul K, et al. prasavGraph: An Android-based e-Partograph. *2016 International Conference on Systems in Medicine and Biology (ICSMB)* 2016:106-09. doi: 10.1109/ICSMB.2016.7915099

16. Usmanova G, Gresh A, Cohen MA, et al. Acceptability and Barriers to Use of the ASMAN Provider-Facing Electronic Platform for Peripartum Care in Public Facilities in Madhya Pradesh and Rajasthan, India: A Qualitative Study Using the Technology Acceptance Model-3. *Int J Environ Res Public Health* 2020;17(22) doi: 10.3390/ijerph17228333 [published Online First: 11 November 2020]

17. Usmanova G, Lalchandani K, Srivastava A, et al. The role of digital clinical decision support tool in improving quality of intrapartum and postpartum care: experiences from two states of India. *BMC Pregnancy Childbirth* 2021;21(1):278. doi: 10.1186/s12884-021-03710-y [published Online First: 7 April 2021]

18. Andreatta P, Debpuur D, Danquah A, et al. Using cell phones to collect postpartum hemorrhage outcome data in rural Ghana. *Int J Gynaecol Obstet* 2011;113(2):148-51. doi: 10.1016/j.ijgo.2010.11.020 [published Online First: 21 March 2011]


For peer review only - http://bmjopen.bmj.com/site/about/guidelines.xhtml

19. Somannavar MS, Goudar SS, Revankar AP, et al. Evaluating time between birth to cry or bag and mask ventilation using mobile delivery room timers in India: the NICHD Global Network's Helping Babies Breathe Trial. *BMC Pediatr* 2015;15:93. doi: 10.1186/s12887-015-0408-6 [published Online First: 6 August 2015]

20. Mumpe-Mwanja D, Barlow-Mosha L, Williamson D, et al. A hospital-based birth defects surveillance system in Kampala, Uganda. *BMC Pregnancy Childbirth* 2019;19(1):372. doi: 10.1186/s12884-019-2542-x [published Online First: 22 October 2019]

21. Ngabo F, Nguimfack J, Nwaigwe F, et al. Designing and Implementing an Innovative SMS-based alert system (RapidSMS-MCH) to monitor pregnancy and reduce maternal and child deaths in Rwanda. *Pan Afr Med J* 2012;13:31. [published Online First: 14 October 2012]

22. Musabyimana A, Ruton H, Gaju E, et al. Assessing the perspectives of users and beneficiaries of a community health worker mHealth tracking system for mothers and children in Rwanda. *PLoS One* 2018;13(6):e0198725. doi: 10.1371/journal.pone.0198725 [published Online First: 7 June 2018]

23. Little A, Medhanyie A, Yebyo H, et al. Meeting community health worker needs for maternal health care service delivery using appropriate mobile technologies in Ethiopia. *PLoS One* 2013;8(10):e77563. doi: 10.1371/journal.pone.0077563 [published Online First: 29 October 2013]

24. Medhanyie AA, Little A, Yebyo H, et al. Health workers' experiences, barriers, preferences and motivating factors in using mHealth forms in Ethiopia. *Hum Resour Health* 2015;13(1):2. doi: 10.1186/1478-4491-13-2 [published Online First: 15 January 2015]

25. Medhanyie AA, Spigt M, Yebyo H, et al. Quality of routine health data collected by health workers using smartphone at primary health care in Ethiopia. *Int J Med Inform* 2017;101:9-14. doi: 10.1016/j.ijmedinf.2017.01.016 [published Online First: 24 January 2017]

26. Stroux L, Martinez B, Coyote Ixen E, et al. An mHealth monitoring system for traditional birth attendant-led antenatal risk assessment in rural Guatemala. *J Med Eng Technol* 2016;40(7-8):356-71. doi: 10.1080/03091902.2016.1223196 [published Online First: 4 October 2016]

27. Martinez B, Hall-Clifford R, Coyote E, et al. Agile Development of a Smartphone App for Perinatal Monitoring in a Resource-Constrained Setting. *J Health Inform Dev Ctries* 2017;11(1)

28. Martinez B, Ixen EC, Hall-Clifford R, et al. mHealth intervention to improve the continuum of maternal and perinatal care in rural Guatemala: a pragmatic, randomized controlled feasibility trial. *Reprod Health* 2018;15(1):120. doi: 10.1186/s12978-018-0554-z [published Online First: 4 July 2018]




For peer review only - http://bmjopen.bmj.com/site/about/guidelines.xhtml

29. Zaidi S, Kazi AM, Riaz A, et al. Operability, Usefulness, and Task-Technology Fit of an mHealth App for Delivering Primary Health Care Services by Community Health Workers in Underserved Areas of Pakistan and Afghanistan: Qualitative Study. *J Med Internet Res* 2020;22(9):e18414. doi: 10.2196/18414 [published Online First: 17 September 2020]

30. Indriani D, Damayanti NA, Teguh D, et al. The maternal referral mobile application system for minimizing the risk of childbirth. *J Public Health Res* 2020;9(2):1813. doi: 10.4081/jphr.2020.1813 [published Online First: 2 July 2020]

31. Velez O, Okyere PB, Kanter AS, et al. A usability study of a mobile health application for rural Ghanaian midwives. *J Midwifery Womens Health* 2014;59(2):184-91. doi: 10.1111/jmwh.12071 [published Online First: 8 January 2014]

32. Tomlinson M, Rotheram-Borus MJ, Swartz L, et al. Scaling up mHealth: where is the evidence? *PLoS Med* 2013;10(2):e1001382. doi: 10.1371/journal.pmed.1001382 [published Online First: 12 February 2013]

33. Agarwal S, Perry HB, Long LA, et al. Evidence on feasibility and effective use of mHealth strategies by frontline health workers in developing countries: systematic review. *Trop Med Int Health* 2015;20(8):1003-14. doi: 10.1111/tmi.12525 [published Online First: 14 May 2015]

34. Balikuddembe MS, Tumwesigye NM, Wakholi PK, et al. Computerized Childbirth Monitoring Tools for Health Care Providers Managing Labor: A Scoping Review. *JMIR Med Inform* 2017;5(2):e14. doi: 10.2196/medinform.6959 [published Online First: 15 June 2017]

FIGURE TITLE/LEGEND:

**Figure 1: screening and selection of articles**

MEDLINE, CINAHL and Global Health were searched for relevant references. These were first screened by citation then full text. Articles selected for inclusion were then hand-searched for relevant references.

For peer review only

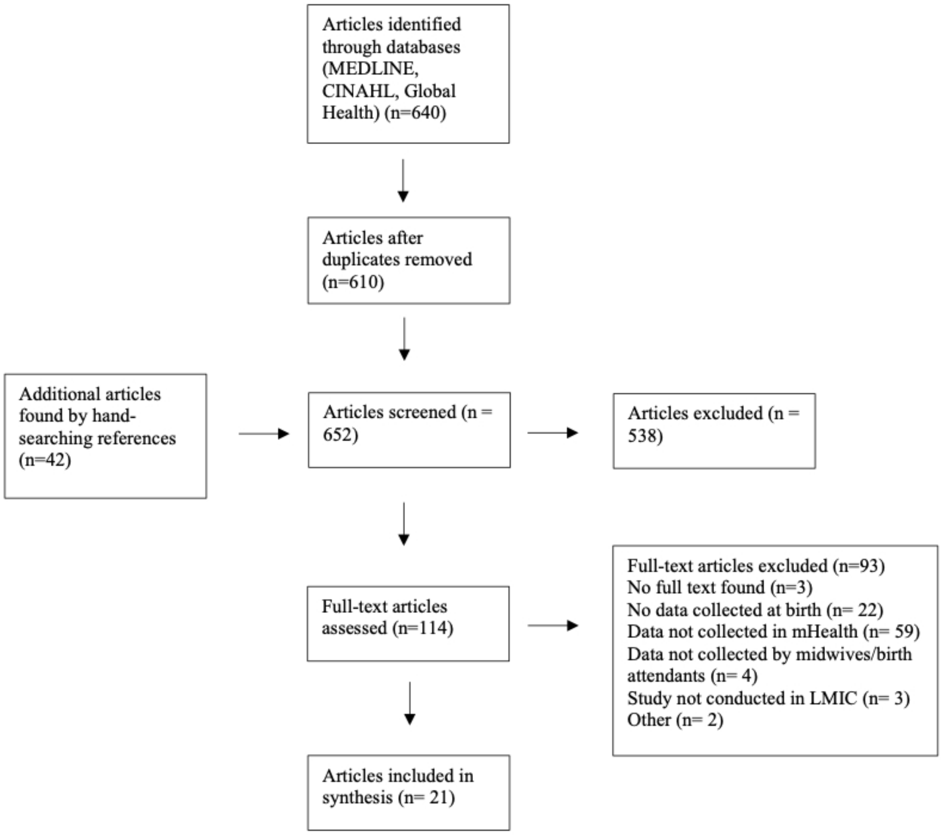


FIGURE LEGEND:
Figure 1: MEDLINE, CINAHL and Global Health were searched for relevant references. These were first screened by citation then full text. Articles selected for inclusion were then hand-searched for relevant references.

FIGURE CAPTION:
Figure 1: Figure 1: screening and selection of articles

295x248mm (72 x 72 DPI)

## *APPENDIX*

Search strategies:

PubMed:

((parturition* OR childbirth* OR labour*) OR ("Parturition"[Mesh])) AND (((((("Electronic Health Records"[Mesh]) OR ( "Mobile Applications"[Mesh] OR "Cell Phone"[Mesh] )) OR ( "Cell Phone Use"[Mesh] OR "Computers, Handheld"[Mesh] )) OR (mobile phone* or mobile* or mhealth* or electronic* or smartphone* or iPhone* or iPad* or tablet computer* or cellphone* or m-health* or ehealth* or e-health* or cell phone* or handheld computer*)) AND ((Africa or Caribbean or "West Indies" or "South America" or "Latin America" or "Central America" or Afghanistan or Albania or Algeria or Angola or "American Samoa" or Armenia or Armenian or Azerbaijan or Bangladesh or Benin or Byelarus or Byelorussian or Belarus or Belorussian or Belorussia or Belize or Bhutan or Bolivia or Bosnia or Herzegovina or Hercegovina or Botswana or Brazil or Brasil or Bulgaria or "Burkina Faso" or "Burkina Fasso" or "Upper Volta" or Burundi or Urundi or Cambodia or "Khmer Republic" or Kampuchea or Cameroon or Cameroons or Cameron or Camerons or "Cape Verde" or "Central African Republic" or Chad or China or Colombia or Comoros or "Comoro Islands" or Comores or Mayotte or Congo or Zaire or "Costa Rica" or "Cote d'Ivoire" or "Ivory Coast" or Cuba or Djibouti or "French Somaliland" or Dominica or "Dominican Republic" or "East Timor" or "East Timur" or "Timor Leste" or Ecuador or Egypt or "United Arab Republic" or "El Salvador" or Eritrea or Ethiopia or Fiji or Gabon or "Gabonese Republic" or Gambia or Gaza or "Georgia Republic" or "Georgian Republic" or Ghana or "Gold Coast" or Grenada or Guatemala or Guinea or Guinea-Bissau or Guam or Guiana or Guyana or Haiti or Honduras or India or Maldives or Indonesia or Iran or Iraq or Jamaica or Jordan or Kazakhstan or Kazakh or Kenya or Kiribati or Korea or Kosovo or Kyrgyzstan or Kirghizia or "Kyrgyz Republic" or Kirghiz or Kirgizstan or "Lao PDR" or Laos or Lebanon or Lesotho or Basutoland or Liberia or Libya or Macedonia or Madagascar or "Malagasy Republic" or Malaysia or Malaya or Malay or Sabah or Sarawak or Malawi or Nyasaland or Mali or "Marshall Islands" or Mauritania or Mauritius or "Agalega Islands" or Mexico or Micronesia or "Middle East" or Moldova or Moldovia or Moldovian or Mongolia or Montenegro or Morocco or Ifni or Mozambique or Myanmar or Myanma or Burma or Namibia or Nepal or "Netherlands Antilles" or Nicaragua or Niger or Nigeria or Pakistan or Palau or Palestine or Panama or "Papua New Guinea" or Paraguay or Peru or Philippines or Philipines or Phillipines or Phillippines or Romania or Rumania or Roumania or Rwanda or Ruanda or "Saint Lucia" or "St Lucia" or "Saint Vincent" or "St Vincent" or Grenadines or Samoa or "Samoan Islands" or "Navigator Island" or "Navigator Islands" or "Sao Tome" or Senegal or Serbia or "Sierra Leone" or Sri Lanka or Ceylon or "Solomon Islands" or Somalia or Sudan or Suriname or Surinam or Swaziland or Syria or Principe or Tajikistan or Tadzhikistan or Tadjikistan or Tadzhik or Tanzania or Thailand or Timor-Leste or Togo or "Togolese Republic" or Tonga or Tunisia or Turkey or Turkmenistan or Turkmen or Tuvalu or Uganda or Ukraine or Uzbekistan or Uzbek or Vanuatu or "New Hebrides" or Vietnam or "Viet Nam" or "West Bank" or Yemen or Zambia or Zimbabwe or Rhodesia or developing countr* or less developed countr* or under developed countr* or underdeveloped countr* or middle income countr* or low income countr* or deprived countr* or underserved countr* or under served countr* or poor countr* or developing nation* or less developed nation* or under developed nation* or underdeveloped nation* or middle income

For peer review only

nation* or low income nation* or deprived nation* or underserved nation* or under served nation* or poor nation* or LMIC or lmics or third world* or lami countr* or transitional countr* or "global health") OR ("Developing Countries"[Mesh]))) AND (("Midwifery"[Mesh]) OR (midwife* or midwives or birth attendant* or doula* or childbirth assistant*)))

Global Health:

((mobile phone* or mobile* or mhealth* or electronic* or smartphone* or iPhone* or iPad* or tablet computer* or cellphone* or m-health* or ehealth* or e-health* or cell phone* or handheld computer).mp. [mp=abstract, title, original title, broad terms, heading words, identifiers, cabicodes] OR (mobile telephones/ OR mobile equipment/)) AND ((midwife* or midwives or birth attendant* or doula* or childbirth assistant*).mp. [mp=abstract, title, original title, broad terms, heading words, identifiers, cabicodes] OR ((traditional birth attendants or midwives).sh)) AND ((childbirth/) OR ((childbirth* or labour*).mp. [mp=abstract, title, original title, broad terms, heading words, identifiers, cabicodes]) AND ((Developing Countries/) OR (Africa or Caribbean or "West Indies" or "South America" or "Latin America" or "Central America" or Afghanistan or Albania or Algeria or Angola or "American Samoa" or Armenia or Armenian or Azerbaijan or Bangladesh or Benin or Byelarus or Byelorussian or Belarus or Belorussian or Belorussia or Belize or Bhutan or Bolivia or Bosnia or Herzegovina or Hercegovina or Botswana or Brazil or Brasil or Bulgaria or "Burkina Faso" or "Burkina Fasso" or "Upper Volta" or Burundi or Urundi or Cambodia or "Khmer Republic" or Kampuchea or Cameroon or Cameroons or Cameron or Camerons or "Cape Verde" or "Central African Republic" or Chad or China or Colombia or Comoros or "Comoro Islands" or Comores or Mayotte or Congo or Zaire or "Costa Rica" or "Cote d'Ivoire" or "Ivory Coast" or Cuba or Djibouti or "French Somaliland" or Dominica or "Dominican Republic" or "East Timor" or "East Timur" or "Timor Leste" or Ecuador or Egypt or "United Arab Republic" or "El Salvador" or Eritrea or Ethiopia or Fiji or Gabon or "Gabonese Republic" or Gambia or Gaza or "Georgia Republic" or "Georgian Republic" or Ghana or "Gold Coast" or Grenada or Guatemala or Guinea or Guinea-Bissau or Guam or Guiana or Guyana or Haiti or Honduras or India or Maldives or Indonesia or Iran or Iraq or Jamaica or Jordan or Kazakhstan or Kazakh or Kenya or Kiribati or Korea or Kosovo or Kyrgyzstan or Kirghizia or "Kyrgyz Republic" or Kirghiz or Kirgizstan or "Lao PDR" or Laos or Lebanon or Lesotho or Basutoland or Liberia or Libya or Macedonia or Madagascar or "Malagasy Republic" or Malaysia or Malaya or Malay or Sabah or Sarawak or Malawi or Nyasaland or Mali or "Marshall Islands" or Mauritania or Mauritius or "Agalega Islands" or Mexico or Micronesia or "Middle East" or Moldova or Moldovia or Moldovian or Mongolia or Montenegro or Morocco or Ifni or Mozambique or Myanmar or Myanma or Burma or Namibia or Nepal or "Netherlands Antilles" or Nicaragua or Niger or Nigeria or Pakistan or Palau or Palestine or Panama or "Papua New Guinea" or Paraguay or Peru or Philippines or Philipines or Phillipines or Phillippines or Romania or Rumania or Roumania or Rwanda or Ruanda or "Saint Lucia" or "St Lucia" or "Saint Vincent" or "St Vincent" or Grenadines or Samoa or "Samoan Islands" or "Navigator Island" or "Navigator Islands" or "Sao Tome" or Senegal or Serbia or "Sierra Leone" or Sri Lanka or Ceylon or "Solomon Islands" or Somalia or Sudan or Suriname or Surinam or Swaziland or Syria or Principe or Tajikistan or Tadzhikistan or Tadjikistan or Tadzhik or Tanzania or Thailand or Timor-Leste or Togo or "Togolese Republic" or Tonga or Tunisia or Turkey or Turkmenistan or Turkmen or Tuvalu or Uganda or Ukraine or Uzbekistan or Uzbek or Vanuatu or "New Hebrides" or Vietnam or "Viet Nam" or "West Bank" or Yemen or Zambia or

For peer review only

Zimbabwe or Rhodesia or developing countr* or less developed countr* or under developed countr* or underdeveloped countr* or middle income countr* or low income countr* or deprived countr* or underserved countr* or under served countr* or poor countr* or developing nation* or less developed nation* or under developed nation* or underdeveloped nation* or middle income nation* or low income nation* or deprived nation* or underserved nation* or under served nation* or poor nation* or LMIC or lmics or third world* or lami countr* or transitional countr* or "global health").mp. [mp=abstract, title, original title, broad terms, heading words, identifiers, cabicodes])

CINAHL:

( mh:("Midwifery") OR (midwife* or midwives or birth attendant* or doula* or childbirth assistant*)) AND (mh:(“Labour”) OR (childbirth* OR labour*)) AND (mh:(“Cellular Phone” OR “Mobile Applications” OR “Electronic Health Records” OR “Computers, Hand-Held” OR “Telemedicine”) OR (mobile phone* or mobile* or mhealth* or electronic* or smartphone* or iPhone* or iPad* or tablet computer* or cellphone* or m-health* or ehealth* or e-health* or cell phone* or handheld computer*)) AND (mh:(“Developing Countries”) OR (Africa or Caribbean or "West Indies" or "South America" or "Latin America" or "Central America" or Afghanistan or Albania or Algeria or Angola or "American Samoa" or Armenia or Armenian or Azerbaijan or Bangladesh or Benin or Byelarus or Byelorussian or Belarus or Belorussian or Belorussia or Belize or Bhutan or Bolivia or Bosnia or Herzegovina or Hercegovina or Botswana or Brazil or Brasil or Bulgaria or "Burkina Faso" or "Burkina Fasso" or "Upper Volta" or Burundi or Urundi or Cambodia or "Khmer Republic" or Kampuchea or Cameroon or Cameroons or Cameron or Camerons or "Cape Verde" or "Central African Republic" or Chad or China or Colombia or Comoros or "Comoro Islands" or Comores or Mayotte or Congo or Zaire or "Costa Rica" or "Cote d'Ivoire" or "Ivory Coast" or Cuba or Djibouti or "French Somaliland" or Dominica or "Dominican Republic" or "East Timor" or "East Timur" or "Timor Leste" or Ecuador or Egypt or "United Arab Republic" or "El Salvador" or Eritrea or Ethiopia or Fiji or Gabon or "Gabonese Republic" or Gambia or Gaza or "Georgia Republic" or "Georgian Republic" or Ghana or "Gold Coast" or Grenada or Guatemala or Guinea or Guinea-Bissau or Guam or Guiana or Guyana or Haiti or Honduras or India or Maldives or Indonesia or Iran or Iraq or Jamaica or Jordan or Kazakhstan or Kazakh or Kenya or Kiribati or Korea or Kosovo or Kyrgyzstan or Kirghizia or "Kyrgyz Republic" or Kirghiz or Kirgizstan or "Lao PDR" or Laos or Lebanon or Lesotho or Basutoland or Liberia or Libya or Macedonia or Madagascar or "Malagasy Republic" or Malaysia or Malaya or Malay or Sabah or Sarawak or Malawi or Nyasaland or Mali or "Marshall Islands" or Mauritania or Mauritius or "Agalega Islands" or Mexico or Micronesia or "Middle East" or Moldova or Moldovia or Moldovian or Mongolia or Montenegro or Morocco or Ifni or Mozambique or Myanmar or Myanma or Burma or Namibia or Nepal or "Netherlands Antilles" or Nicaragua or Niger or Nigeria or Pakistan or Palau or Palestine or Panama or "Papua New Guinea" or Paraguay or Peru or Philippines or Philipines or Phillipines or Phillippines or Romania or Rumania or Roumania or Rwanda or Ruanda or "Saint Lucia" or "St Lucia" or "Saint Vincent" or "St Vincent" or Grenadines or Samoa or "Samoan Islands" or "Navigator Island" or "Navigator Islands" or "Sao Tome" or Senegal or Serbia or "Sierra Leone" or Sri Lanka or Ceylon or "Solomon Islands" or Somalia or Sudan or Suriname or Surinam or Swaziland or Syria or Principe or Tajikistan or Tadzhikistan or Tadjikistan or Tadzhik or Tanzania or Thailand or Timor-Leste or Togo or "Togolese Republic" or Tonga or Tunisia or Turkey or Turkmenistan or Turkmen or Tuvalu or Uganda or Ukraine or Uzbekistan or Uzbek or Vanuatu or "New Hebrides" or Vietnam or "Viet Nam" or "West Bank" or

For peer review only

Yemen or Zambia or Zimbabwe or Rhodesia or developing countr* or less developed countr* or under developed countr* or underdeveloped countr* or middle income countr* or low income countr* or deprived countr* or underserved countr* or under served countr* or poor countr* or developing nation* or less developed nation* or under developed nation* or underdeveloped nation* or middle income nation* or low income nation* or deprived nation* or underserved nation* or under served nation* or poor nation* or LMIC or lmics or third world* or lami countr* or transitional countr* or "global health"))

For peer review only

## Preferred Reporting Items for Systematic reviews and Meta-Analyses extension for Scoping Reviews (PRISMA-ScR) Checklist

| SECTION | ITEM | PRISMA-ScR CHECKLIST ITEM | REPORTED ON PAGE # |
|---|---|---|---|
| **TITLE** | | | |
| Title | 1 | Identify the report as a scoping review. | 3 |
| **ABSTRACT** | | | |
| Structured summary | 2 | Provide a structured summary that includes (as applicable): background, objectives, eligibility criteria, sources of evidence, charting methods, results, and conclusions that relate to the review questions and objectives. | 1 |
| **INTRODUCTION** | | | |
| Rationale | 3 | Describe the rationale for the review in the context of what is already known. Explain why the review questions/objectives lend themselves to a scoping review approach. | 3 |
| Objectives | 4 | Provide an explicit statement of the questions and objectives being addressed with reference to their key elements (e.g., population or participants, concepts, and context) or other relevant key elements used to conceptualize the review questions and/or objectives. | 4 |
| **METHODS** | | | |
| Protocol and registration | 5 | Indicate whether a review protocol exists; state if and where it can be accessed (e.g., a Web address); and if available, provide registration information, including the registration number. | 4 |
| Eligibility criteria | 6 | Specify characteristics of the sources of evidence used as eligibility criteria (e.g., years considered, language, and publication status), and provide a rationale. | 4-5 |
| Information sources* | 7 | Describe all information sources in the search (e.g., databases with dates of coverage and contact with authors to identify additional sources), as well as the date the most recent search was executed. | 4-5 |
| Search | 8 | Present the full electronic search strategy for at least 1 database, including any limits used, such that it could be repeated. | 25-28 |
| Selection of sources of evidence† | 9 | State the process for selecting sources of evidence (i.e., screening and eligibility) included in the scoping review. | 4-6 |
| Data charting process‡ | 10 | Describe the methods of charting data from the included sources of evidence (e.g., calibrated forms or forms that have been tested by the team before their use, and whether data charting was done independently or in duplicate) and any processes for obtaining and confirming data from investigators. | 4-6 |
| Data items | 11 | List and define all variables for which data were sought and any assumptions and simplifications made. | 5 |
| Critical appraisal of individual sources of evidence§ | 12 | If done, provide a rationale for conducting a critical appraisal of included sources of evidence; describe the methods used and how this information was used in any data synthesis (if appropriate). | NA |


For peer review only - http://bmjopen.bmj.com/site/about/guidelines.xhtml

| SECTION | ITEM | PRISMA-ScR CHECKLIST ITEM | REPORTED ON PAGE # |
|---|---|---|---|
| Synthesis of results | 13 | Describe the methods of handling and summarizing the data that were charted. | 5 |
| **RESULTS** | | | |
| Selection of sources of evidence | 14 | Give numbers of sources of evidence screened, assessed for eligibility, and included in the review, with reasons for exclusions at each stage, ideally using a flow diagram. | 5-6 |
| Characteristics of sources of evidence | 15 | For each source of evidence, present characteristics for which data were charted and provide the citations. | 20-24 |
| Critical appraisal within sources of evidence | 16 | If done, present data on critical appraisal of included sources of evidence (see item 12). | NA |
| Results of individual sources of evidence | 17 | For each included source of evidence, present the relevant data that were charted that relate to the review questions and objectives. | 7-12, 17-24 |
| Synthesis of results | 18 | Summarize and/or present the charting results as they relate to the review questions and objectives. | 7-12 |
| **DISCUSSION** | | | |
| Summary of evidence | 19 | Summarize the main results (including an overview of concepts, themes, and types of evidence available), link to the review questions and objectives, and consider the relevance to key groups. | 12-13 |
| Limitations | 20 | Discuss the limitations of the scoping review process. | 13 |
| Conclusions | 21 | Provide a general interpretation of the results with respect to the review questions and objectives, as well as potential implications and/or next steps. | 13 |
| **FUNDING** | | | |
| Funding | 22 | Describe sources of funding for the included sources of evidence, as well as sources of funding for the scoping review. Describe the role of the funders of the scoping review. | 13 |

JBI = Joanna Briggs Institute; PRISMA-ScR = Preferred Reporting Items for Systematic reviews and Meta-Analyses extension for Scoping Reviews.

* Where *sources of evidence* (see second footnote) are compiled from, such as bibliographic databases, social media platforms, and Web sites.

† A more inclusive/heterogeneous term used to account for the different types of evidence or data sources (e.g., quantitative and/or qualitative research, expert opinion, and policy documents) that may be eligible in a scoping review as opposed to only studies. This is not to be confused with *information sources* (see first footnote).

‡ The frameworks by Arksey and O'Malley (6) and Levac and colleagues (7) and the JBI guidance (4, 5) refer to the process of data extraction in a scoping review as data charting.

§ The process of systematically examining research evidence to assess its validity, results, and relevance before using it to inform a decision. This term is used for items 12 and 19 instead of "risk of bias" (which is more applicable to systematic reviews of interventions) to include and acknowledge the various sources of evidence that may be used in a scoping review (e.g., quantitative and/or qualitative research, expert opinion, and policy document).

*From:* Tricco AC, Lillie E, Zarin W, O'Brien KK, Colquhoun H, Levac D, et al. PRISMA Extension for Scoping Reviews (PRISMAScR): Checklist and Explanation. Ann Intern Med. 2018;169:467–473. doi: 10.7326/M18-0850.



For peer review only - http://bmjopen.bmj.com/site/about/guidelines.xhtml
