# Supplementary material for: Use of mHealth tools to register birth outcomes in low-income and middle-income countries: a scoping review
Source: BMJ Open. 2022 Oct 12;12(10):e063886. doi: 10.1136/bmjopen-2022-063886 (PMC9562304; doi:10.1136/bmjopen-2022-063886)
Supplement: Reviewer comments [file bmjopen-2022-063886.reviewer_comments.pdf]

PEER REVIEW HISTORY

BMJ Open publishes all reviews undertaken for accepted manuscripts. Reviewers are asked to complete a checklist review form (**http://bmjopen.bmj.com/site/about/resources/checklist.pdf**) and are provided with free text boxes to elaborate on their assessment. These free text comments are reproduced below.

ARTICLE DETAILS

**TITLE (PROVISIONAL)**	The use of mHealth tools to register birth outcomes in low- and middle-income countries: a scoping review
**AUTHORS**	Cansdale, Lottie; Kelly, Gabriella; Khashan, Ali; Malata, Address; Kachale, Fannie; Lissauer, David; Yosefe, Simeon; Roberts, James; Woodworth, Simon; Mmbaga, Blandina; Redman, Christopher; Hirst, Jane Elizabeth

VERSION 1 – REVIEW

**REVIEWER**	Rahman, Md. Obaidur<br>National Institute of Infectious Diseases, Center for Surveillance, Immunization, and Epidemiologic Research
**REVIEW RETURNED**	27-Jun-2022

**GENERAL COMMENTS**	This scoping review tried to capture the potential use and evidence of digital birth registries using mobile technology in low- and middle-income countries (LMICs). Such kinds of technologies can be useful not only to birth surveillance in a cost-effective and timely manner in resource-limited settings, but also to take effective actions among clinicians or policymakers to improve adverse birth outcomes in the places identified. Although the authors tried to address the issue highlighted, there are several generals, intellectual, and methodological issues which urgently need to be addressed. I would like to make the following comments and suggestions which would improve the quality and readability of the manuscript.<br><br>1) The title of the study did not align directly with the review objective or its findings. It would be better to revise the title based on the study focused like “The use and evidence of mHealth tools to register birth outcomes in low- and middle-income countries: a scoping review”.<br><br>2) In objective section of the abstract, author should separate introduction and objective of the review or follow the journal guideline. mHealth generally refers to mobile health. Although author define it in the main text in the similar way but it didn’t reflect in abstract. Author should also mention database searching date and data synthesis approach briefly in the method section of the abstract. Author should report studies, not papers (the whole manuscript needs to be revised accordingly).<br><br>3) In the results section of the abstract, author should report number of studies for each relevant case. For example- author identified six themes, they need to report how many studies reported each theme. Author should report mostly quantitative way as much as possible in the result sections.

	4) In the conclusion section of the abstract, author reported that none are purposed specifically to record birth outcomes at delivery, which contradicts with the statement of the results section of the abstract. Author needs further explanation either in results section or in conclusion. The conclusion should be based on the review findings, that was partly reflected. The whole manuscript needs to be revised accordingly.<br><br>5) Introduction: Author fails to provide a strong rationale of the review. For example- this review focused on LMICs but in the first paragraph of the introduction section, they highlight the context of high-income countries but did not relate to LMICs. Author should follow a recent systematic review and meta-analysis focused on mHealth interventions and skilled delivery care utilization in LMICs (https://www.jmir.org/2022/4/e34061; Effects of mHealth Interventions on Improving Antenatal Care Visits and Skilled Delivery Care in Low- and Middle-Income Countries: Systematic Review and Meta-analysis) that can be useful to them and can be cited as well to explain their rationale. In line 116-117, author should have few recent citations.<br><br>6) Methods: Did author register this scoping review in OSF (https://osf.io/)? As they did not publish review protocol, it is suggested to be register their protocol on OSF. Furthermore, PCC should be under a heading/sub-heading like review protocol registration and guideline. Also, the first sentence of the line 134 is irrelevant here.<br><br>7) In the line 149-155, author should include search concepts including key search terms and also provide the search history as appendix, although they provide search strategy in appendix there. Furthermore, author provide search strategy for PubMed database but mentioned MEDLINE here. Although PubMed includes MEDLINE, the reporting should be consistent and unique.<br><br>8) In the line 179-192, author should mention about their plan of study quality assessment.<br><br>9) The statement of the line 199 should be mentioned in the method section and Figure 1 as they updated their search strategy and should report how many new studies they identified between these periods.<br><br>10) In the line 256, author should mention what are the 15 different mHealth technologies they identified in their review and which countries with their number of studies.<br><br>11) In the line 271, author mentioned 3 main roles for digital tools at birth but in abstract they classified 6 themes, which should be consistent.<br><br>12) Author should precisely describe their study findings with their proper citation in the result section. This section needs a significant improvement.<br><br>13) In discussion section (line 465), author reported 20 papers identified but it would be 21 studies as reported in Figure 1 and result sections.

	14) The tables were not rightly placed. Author should check their reference styles, few references seem to be inconsistent. In addition, author should follow the later version of PRISMA flow chart. Author should follow the journal guidelines as well.<br><br>15) The whole manuscript has several typo-error, and academic English editing is also required.

**REVIEWER**	Takahashi, K<br>Teikyo University Graduate School of Public Health
**REVIEW RETURNED**	28-Jul-2022

**GENERAL COMMENTS**	This article reads like a perfect literature for scoping review.<br>I have a few comments for amendment.<br><br>1. Did the authors conduct hand search?<br>If not, please explain the reason in the method section.<br><br>Because of budget limitation, some academic documents of LMICs are disclosed in PDF styles.<br><br>Also, please explain the reason why the authors did not include preprint.<br><br>2.Conclusion of abstract.<br>One of the role of scoping review is identification of research gaps in existing theme. This authors can change the text “Further research is needed,,,”. to “<br>More research is needed” because it is very confusing.

VERSION 1 – AUTHOR RESPONSE

Reviewer: 1
Dr. Md. Obaidur Rahman, National Institute of Infectious Diseases
Comments to the Author:
This scoping review tried to capture the potential use and evidence of digital birth registries using mobile technology in low- and middle-income countries (LMICs). Such kinds of technologies can be useful not only to birth surveillance in a cost-effective and timely manner in resource-limited settings, but also to take effective actions among clinicians or policymakers to improve adverse birth outcomes in the places identified. Although the authors tried to address the issue highlighted, there are several generals, intellectual, and methodological issues which urgently need to be addressed. I would like to make the following comments and suggestions which would improve the quality and readability of the manuscript.

1) The title of the study did not align directly with the review objective or its findings. It would be better to revise the title based on the study focused like “The use and evidence of mHealth tools to register birth outcomes in low- and middle-income countries: a scoping review”.

We agree with this suggestion, and have updated the title to ‘The use of mHealth tools to register birth outcomes in low- and middle-income countries: a scoping review’.

2) In objective section of the abstract, author should separate introduction and objective of the review or follow the journal guideline. mHealth generally refers to mobile health. Although author define it in the main text in the similar way but it didn’t reflect in abstract. Author should also mention database searching date and data synthesis approach briefly in the method section of the abstract. Author should report studies, not papers (the whole manuscript needs to be revised accordingly).

The abstract has been updated to meet journal guidelines, which now includes database searching and data synthesis, but does not allow for separate introduction and objective section. We have also revised the language to report studies, rather than papers.

3) In the results section of the abstract, author should report number of studies for each relevant case. For example- author identified six themes, they need to report how many studies reported each theme. Author should report mostly quantitative way as much as possible in the result sections.

The abstract now includes the number of studies reporting each theme.

4) In the conclusion section of the abstract, author reported that none are purposed specifically to record birth outcomes at delivery, which contradicts with the statement of the results section of the abstract. Author needs further explanation either in results section or in conclusion. The conclusion should be based on the review findings, that was partly reflected. The whole manuscript needs to be revised accordingly.

We have changed the language to make it clear that whilst many technologies are capable of recording birth outcomes at the point of delivery, we identified no technologies that had been designed specifically for that purpose.

5) Introduction: Author fails to provide a strong rationale of the review. For example- this review focused on LMICs but in the first paragraph of the introduction section, they highlight the context of high-income countries but did not relate to LMICs. Author should follow a recent systematic review and meta-analysis focused on mHealth interventions and skilled delivery care utilization in LMICs (https://www.jmir.org/2022/4/e34061; Effects of mHealth Interventions on Improving Antenatal Care Visits and Skilled Delivery Care in Low- and Middle-Income Countries: Systematic Review and Meta-analysis) that can be useful to them and can be cited as well to explain their rationale. In line 116-117, author should have few recent citations.

The opening paragraph of the introduction now includes more reference to the LMIC context, line 180. We have cited reviews that focus on the use of mHealth in LMICs, line 199.

6) Methods: Did author register this scoping review in OSF (https://osf.io/)? As they did not publish review protocol, it is suggested to be register their protocol on OSF. Furthermore, PCC should be under a heading/sub-heading like review protocol registration and guideline. Also, the first sentence of the line 134 is irrelevant here.

We did not publish a review protocol, stated in line 232. PCC has been put under the subheading 'Population, Concept, Context'.

7) In the line 149-155, author should include search concepts including key search terms and also provide the search history as appendix, although they provide search strategy in appendix there. Furthermore, author provide search strategy for PubMed database but mentioned MEDLINE here. Although PubMed includes MEDLINE, the reporting should be consistent and unique.

Line 254 now includes some key terms from the search strategy. PubMed database was searched, and we have changed this in the manuscript.

8) In the line 179-192, author should mention about their plan of study quality assessment.

Lines 312-313 explain our rationale for not assessing the quality of studies.

9) The statement of the line 199 should be mentioned in the method section and Figure 1 as they updated their search strategy and should report how many new studies they identified between these periods.

Line 321 reports how many new studies were identified between the two periods.

10) In the line 256, author should mention what are the 15 different mHealth technologies they identified in their review and which countries with their number of studies.

Thank you for this suggestion. We considered including the names and countries in the text, and in one of our draft manuscripts, but it became difficult to read, and the information contained in the table is far easier to read. If the reviewer would prefer that we list them in the text, we would be able to do so.

11) In the line 271, author mentioned 3 main roles for digital tools at birth but in abstract they classified 6 themes, which should be consistent.

We have updated the language in lines 549-553 to reflect that the six themes were split into 3 main tools and 3 main objectives of studies.

12) Author should precisely describe their study findings with their proper citation in the result section. This section needs a significant improvement.

We are not sure if we understood this comment correctly, but citations have been checked for the results section and are correct. We are happy to address this comment further if needed.

13) In discussion section (line 465), author reported 20 papers identified but it would be 21 studies as reported in Figure 1 and result sections.

Thank you for spotting this error, which has been corrected.

14) The tables were not rightly placed. Author should check their reference styles, few references seem to be inconsistent. In addition, author should follow the later version of PRISMA flow chart. Author should follow the journal guidelines as well.

We have followed both journal guidelines and the PRISMA flowchart in preparing the manuscript. The tables have been re-formatted and are correctly placed as requested. References have been checked to ensure consistency.

15) The whole manuscript has several typo-error, and academic English editing is also required.

The manuscript has been prepared with the use of an automatic spell-check and now hand-checked by the authors for both typo-errors and academic English use.

Reviewer: 2
Dr. K Takahashi, Teikyo University Graduate School of Public Health
Comments to the Author:
This article reads like a perfect literature for scoping review.
I have a few comments for amendment.

1. Did the authors conduct hand search?
If not, please explain the reason in the method section.

Because of budget limitation, some academic documents of LMICs are disclosed in PDF styles.

Also, please explain the reason why the authors did not include preprint.

We hand-searched references of included articles, as stated in line 265, but did not search grey literature. Our inclusion criteria was studies being peer-reviewed, as stated in line 296. We have now added a statement in the Discussion to acknowledge this limitation.

2.Conclusion of abstract.
One of the role of scoping review is identification of research gaps in existing theme. This authors can change the text “Further research is needed,,,”. to “
More research is needed” because it is very confusing.

This language has been changed as suggested.

Thank you so much for your time and effort in reviewing our manuscript.

VERSION 2 – REVIEW

**REVIEWER**	Rahman, Md. Obaidur<br>National Institute of Infectious Diseases, Center for Surveillance, Immunization, and Epidemiologic Research
**REVIEW RETURNED**	26-Sep-2022

**GENERAL COMMENTS**	The author tried to address reviewers' comments. There are still remained some concerns that needs to be addressed.<br><br>1) In abstract, you mentioned that conventional paper-based registers could be replaced by mobile (mHealth) tools. Mobile tools and mHealth tools are not same. It is better to mention mobile health (mHealth) tools. Also, you should mention exact search period in data sources section like "…. from January 2000 to March 2022". In results section, you did not summarize the study findings, therefore, it is unclear the capability of mHealth tools to record birth outcomes. It is better to include the findings of the studies in the results section.<br><br>2) Introduction: Author still could not provide a strong rationale of the review. Author should add prior systematic review findings in the last paragraph, and then identify the research gap. Also, you should mention what are the 15 mHealth tools identified by the prior systematic review.<br><br>3) Author mixed up sentence case with capitalize each word in the heading of the paragraph. Also, there has an inconsistency in the reference style. Please ensure their consistency.

VERSION 2 – AUTHOR RESPONSE

Reviewer: 1
Dr. Md. Obaidur Rahman, National Institute of Infectious Diseases

1) In abstract, you mentioned that conventional paper-based registers could be replaced by mobile (mHealth) tools. Mobile tools and mHealth tools are not same. It is better to mention mobile health (mHealth) tools. Also, you should mention exact search period in data sources section like "…. from January 2000 to March 2022" [NOTE FROM THE EDITORS: As there was no formal start date on the search, this should be stated in the abstract, to avoid confusion]. In results section, you did not summarize the study findings, therefore, it is unclear the capability of mHealth tools to record birth outcomes. It is better to include the findings of the studies in the results section.

Thank you for this suggestion. We have updated the language regarding mHealth accordingly. Regarding the abstract search period, we have changed the language in the abstract to reflect there being no formal start date.

2) Introduction: Author still could not provide a strong rationale of the review. Author should add prior systematic review findings in the last paragraph, and then identify the research

gap. Also, you should mention what are the 15 mHealth tools identified by the prior systematic review.

We have amended the final paragraph of the introduction to include the prior systematic review findings and the research gap that our review fills.

3) Author mixed up sentence case with capitalize each word in the heading of the paragraph. Also, there has an inconsistency in the reference style. Please ensure their consistency.

Thank you for directing us to this. The references have been updated according to the journal guidelines, and the paragraph headings have been amended.
